# Accurate Identification of Degraded Products of Aflatoxin B_1_ Under UV Irradiation Based on UPLC-Q-TOF-MS/MS and NMR Analysis

**DOI:** 10.3389/fchem.2021.789249

**Published:** 2021-11-24

**Authors:** Yan-Duo Wang, Cheng-Gang Song, Jian Yang, Tao Zhou, Yu-Yang Zhao, Jian-Chun Qin, Lan-Ping Guo, Gang Ding

**Affiliations:** ^1^ Key Laboratory of Bioactive Substances and Resources Utilization of Chinese Herbal Medicine, Ministry of Education, Institute of Medicinal Plant Development, Chinese Academy of Medical Sciences and Peking Union Medical College, Beijing, China; ^2^ College of Plant Sciences, Jilin University, Changchun, China; ^3^ State Key Laboratory Breeding Base of Dao-di Herbs, National Resource Center for Chinese Materia Medica, China Academy of Chinese Medical Sciences, Beijing, China; ^4^ Guizhou University of Traditional Chinese Medicine, Guiyang, China

**Keywords:** aflatoxin B1, UPLC-Q-TOF-MS/MS, degraded products, purification, NMR

## Abstract

Analysis, purification, and characterization of AFB_1_ degraded products are vital steps for elucidation of the photocatalytic mechanism. In this report, the UPLC-Q-TOF-MS/MS technique was first coupled with purification and NMR spectral approaches to analyze and characterize degraded products of AFB_1_ photocatalyzed under UV irradiation. A total of seventeen degraded products were characterized based on the UPLC-Q-TOF-MS/MS analysis, in which seven ones (1–7) including four (stereo) isomers (1,2, 5, and 6) were purified and elucidated by NMR experiments. According to the structural features of AFB_1_ and degraded products (1–7), the possible photocatalytic mechanisms were suggested. Furthermore, AFB_1_ and degraded products (1–7) were evaluated against different cell lines. The results indicated that the UPLC-Q-TOF-MS/MS technique combined with purification, NMR spectral experiments, and biological tests was an applicable integrated approach for analysis, characterization, and toxic evaluation of degraded products of AFB_1_, which could be used to evaluate other mycotoxin degradation processes.

## Introduction

Aflatoxins (AFBs), a group of mycotoxins (including AFB_1_, AFB_2_, AFBG_1_, AFG_2_, and other derivatives) with highly toxic, mutagenic, and carcinogenic activities, are mainly produced by *Aspergillus flavus* and *A. parasiticus* (Massey et al., 1995; Rustom, 1997). These two fungi could infect plants, grains, food, and animals which could lead to significant food safety problems and economic losses. The core skeleton of AFBs is dihydrofuro [2,3-b]furan combined with a coumarin ring, in which the double bond on the furan ring is the key toxic group. The double bond (C-8/C-9) could be transformed to AFB-8,9-epoxide in the human body, which then quickly combines with DNA, glutathione *S*-transferase, or N7 guanine to form highly toxic adducts (Garner et al., 1971; Essigmann et al., 1977; Lin et al., 1977; Croy et al., 1978).

Aflatoxin B_1_ is the most notorious type with potential teratogenic, mutagenic, and hepatocarcinogenic toxicity, and it is classified as a group I carcinogen by the International Agency for Research in Cancer (IARC) (Cancer, 1993). Thus, degradation or reduction of AFB_1_ becomes a hot spot worldwide. Diverse approaches including physical, chemical, and biological methods are used to degrade or reduce AFBs (Alberts et al., 2009; Mendez-Albores et al., 2009; Liu et al., 2010; Liu et al., 2011; Luo et al., 2014; Kumar et al., 2017; Peng et al., 2018). Physical methods mainly include high temperature, irradiation, adsorption, and ultrasonic methods, among which UV irradiation is often employed as an effective method to degrade or reduce AFBs based on the photosensitive characteristics (Calado et al., 2014). Liu investigated the photodegradation of AFB_1_ in water/acetonitrile solution and characterized three degraded products based on UPLC-Q-TOF MS data (Liu et al., 2010). Later, they analyzed AFB_1_ photodegradation in peanut oil under UV irradiation and concluded that the mutagenic effects of UV-treated samples were completely lost compared with those of untreated samples (Liu et al., 2011). Mao analyzed the degraded products of AFB_1_ in peanut oil using the UPLC-Q-TOF-MS/MS technique (Mao et al., 2016). Wang investigated the degraded products using the LC-MS/MS approach and postulated toxicity of AFB_1_ in methanol–water solution irradiated with Co^60^ gamma-rays (Wang et al., 2011). Recently, Li’s group investigated the photodegraded inactivation mechanism of the hypertoxic site in aflatoxin B_1_ by HPLC-MS (Mao et al., 2019).

Obtaining pure AFB_1_-degraded products and elucidating their structures are very important to establish the photodegradation mechanism and toxic evaluation. Usually, due to limited amounts, purification of AFB_1_-degraded products was significantly difficult. Thus, most mycotoxin-degraded products were mainly characterized by LC-MS/MS techniques without further separation. The LC-MS/MS technique is a high-efficient and sensitive approach for analysis and structural characterization of different metabolites in mixtures, which is often used to dereplicate or detect new compounds from extracts or characterize mycotoxin-degraded products. Yet, this technique could not differentiate (stereo) isomers easily. The nuclear magnetic resonance (NMR) spectral technique is a standard and universal approach for structural elucidation (Wang et al., 2018; Song et al., 2019; Li et al., 2020; Wang et al., 2020). In this study, UPLC-Q-TOF-MS/MS analysis combined with purification and NMR spectral experiments was used to characterize AFB_1_-degraded products under UV irradiation. The possible photocatalytic mechanism was elucidated, and toxicities of AFB_1_ and degraded products (**1**–**7**) were evaluated, which provided a thought for other mycotoxin degradation processes.

## Experimental

### Chemicals and Reagents

Aflatoxin B_1_ was purchased from Pribolab (Qingdao, China). Chromatographic-grade methanol and acetone were obtained from Tianjin Saifu Rui Technology Company (Tianjin, China). Analytical-grade methanol, acetone, and DMSO were obtained from Chron Chemicals (Chengdu, China). For NMR analysis, all deuterium reagents were purchased from Sigma (St. Louis, MO, USA).

Standard solutions of AFB_1_ were placed in a 2-ml sealed centrifugal tube, prepared in methanol–DMSO (9:1 v/v), and fully dissolved in methanol using an ultrasound device from Beijing Tianlin Hengtai Technology Company (Beijing, China), and then, it was submitted to be degraded.

### UV Irradiation

To investigate the degradation of AFB_1_, a UV lamp (20 W, 72 μws/cm^2^, GGZ250-1, Shanghai Jiming Special Lighting Appliance Factory) at 365 nm wavelength was used to perform the irradiation experiments. 18 mg of pure AFB_1_ was added to acetone solvent, and 10 mg of pure AFB_1_ was added to methanol solvent, and both of them were placed in a sealed centrifugal tube and illuminated at room temperature for 45 h (Liu et al., 2010).

### HPLC Operation

The degraded products were analyzed and isolated by semipreparative HPLC on SEP LC-52 with an MWD UV detector (Separation (Beijing) Technology Co. Ltd., Beijing, China) using a 250 mm × 10 mm i. d., 5 μm, ODS-A column (YMC, Kyoto, Japan). The mixture in methanol was purified by semipreparative HPLC (55–60% CH_3_OH in H_2_O, v/v, 2 ml/min, 30 min) and yielded **4** (0.6 mg, *t*_R_ = 18.6 min), **3** (0.5 mg, *t*_R_ = 22.3 min), **1** (0.5 mg, *t*_R_ = 23.8 min), and **2** (0.4 mg, *t*_R_ = 27.0 min), respectively. The mixture in acetone was isolated by semipreparative HPLC (40% CH_3_OH in H_2_O, v/v, 2 ml/min, 3 min; 40–100% CH_3_OH in H_2_O, v/v, 2 ml/min, 20 min) and yielded **5**, **6** (7.0 mg, *t*_R_ = 18.7 min), and **7** (1.5 mg, *t*_R_ = 21.2 min).

### Determination of Degraded Products

The degraded products were identified by NMR experiments. Compounds were analyzed by UPLC-Q-TOF-MS/MS in positive ion mode. 1D and 2D-NMR spectra were acquired using solvent signals (CD_3_OD: *δ*_H_ 3.31/*δ*_C_ 49.9; C_3_D_6_O: *δ*_H_ 2.05/*δ*_C_ 49.9; Pyridine-*d*_5_: *δ*_H_ 8.74, 7.58, 7.22/*δ*_C_ 150.4, 135.9, and 123.9) on a Bruker 600 spectrometer (^1^H: 600 MHz) and a Bruker Avance III 500 spectrometer (^1^H: 500 MHz; ^13^C: 125 MHz) (Bruker, Rheinstetten, Germany).

### UPLC-Q-TOF MS Analysis

AFB_1_ and degraded products were analyzed using a UPLC-Q-TOF-MS/MS system (Waters, United States). Chromatographic analysis was carried out with a Waters Acquity UPLC-PDA system equipped with an analytical reverse-phase C-18 column (2.1 × 100 mm, 1.7 μm, Acquity BEH, Waters, United States) with an absorbance range of 200–400 nm. The column temperature was maintained at 40°C. 0.1% formic acid in water (A) and 0.1% formic acid in acetonitrile (B) were used as the mobile phase. The gradient conditions were as follows: 0–10 min, 10 %–60% B; 10–12.5 min, 60 %–95% B; and 12.6–15 min, 10% B. The flow rate from the UPLC system into the ESI-Q-TOF-MS detector was 0.3 ml/min. The auto-injected volume was 3 μl. Time-of-flight MS detection was performed with a Waters SYNAPT G2 HDMS (Waters Corp., Manchester, United Kingdom) TOF mass spectrometer combined with an ESI source in the positive ion scan mode. The desolvation temperature was set at 400°C with desolvation gas flow at 600 L/h, and the source temperature was 100°C. The lock mass in all analyses was leucine–enkephalin ([M + H]^+^ = 556.2771), used at a concentration of 0.5 g/ml and infused at a flow rate of 10 L/min. Raw data were acquired using the centroid mode, and the mass range was set from *m/z* 50 to 1200. The capillary voltage was set at 3.0 kV with 40 and 4.0 V of the sample and extraction cone voltage. The collision energy was set as 20 eV for low-energy scan and a ramp from 20 to 30 eV for high-energy scan. The instrument was controlled by MassLynx 4.1 software.

### Toxic Evaluation of Degraded Products and AFB_1_

All the degraded products and AFB_1_ were tested for their cytotoxicity against human normal hepatocytes LO-2 and cancer cell lines Hep-G2 and MCF-7. Cells were incubated in a DMEM high glucose medium (Gibco, USA), added with 10% fetal bovine serum (Gibco, United States) and cultured in a 5% CO_2_ incubator at 37°C. The cytotoxicity tests were performed using the MTS (Promega, United States) (Ahmed et al., 2019).

## Results and Discussion

### UPLC-Q-TOF-MS/MS Base Peak Intensity and the UPLC Chromatogram of Degraded Products

**FIGURE 1:**
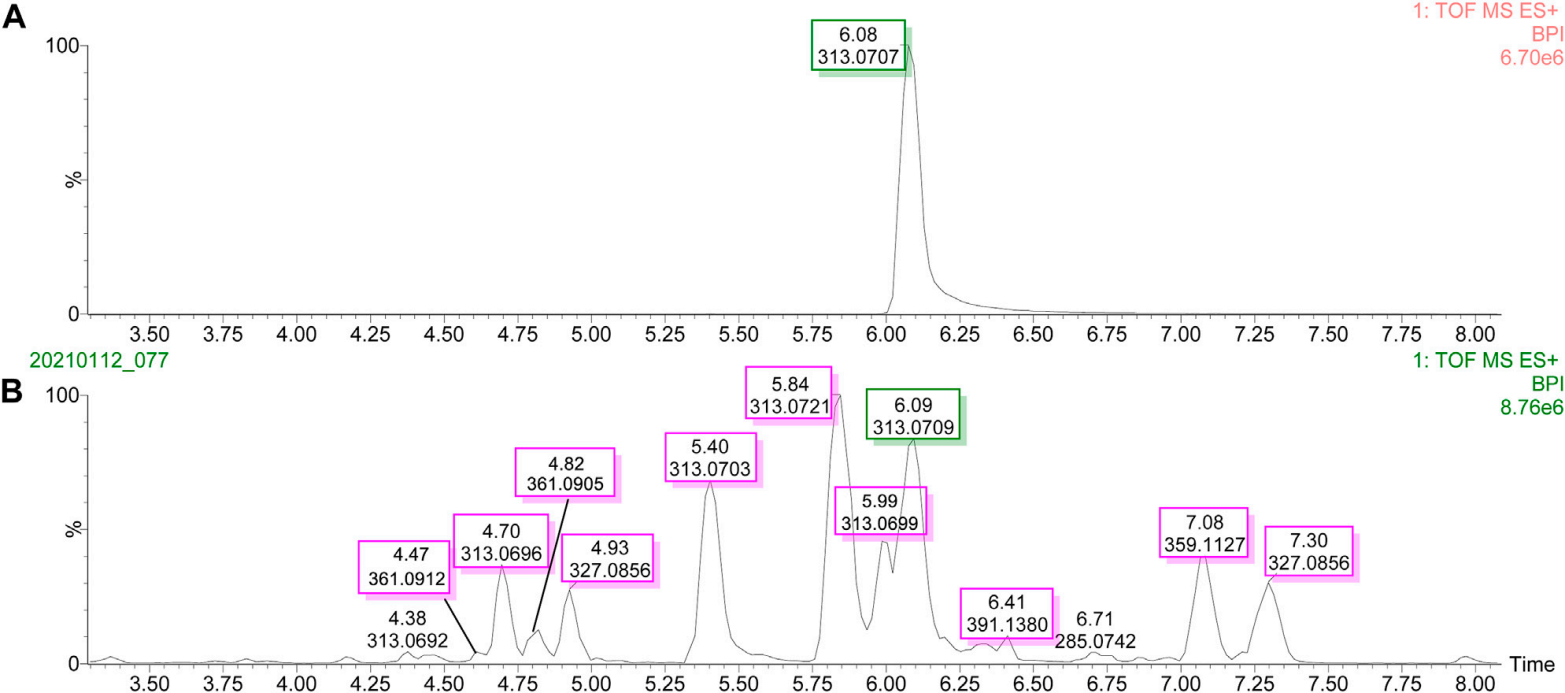
UPLC-Q-TOF-MS/MS profiles of the degradation products of aflatoxin B_1_ in methanol solvent. **(A)** Base peak intensity (BPI) of aflatoxin B_1_. **(B)** Base peak intensity (BPI) of degradation products of aflatoxin B_1_.

**FIGURE 2:**
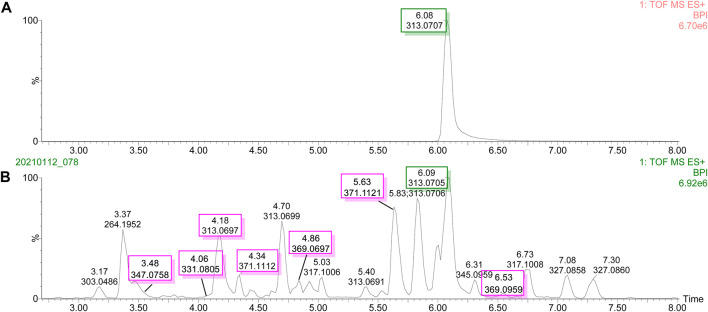
UPLC-Q-TOF-MS/MS profiles of the degradation products of aflatoxin B_1_ in acetone solvent. **(A)** Base peak intensity (BPI) of aflatoxin B_1_. **(B)** Base peak intensity (BPI) of degradation products of aflatoxin B_1_.

The UPLC-Q-TOF-MS/MS BPI of AFB_1_ and its degraded products in methanol–H_2_O and acetone–H_2_O solvents are shown in Figures 1 and 2. The retention time and molecular weight of AFB_1_ were 6.09 min and *m*/*z* 313 ([M+1]), respectively. A series of degraded products with different retention times (RTs) and molecular weights are shown in Table 1. Some ion peaks as (stereo) isomers possessed the same molecular weights (such as *m*/*z* 345) but with different RTs.

**TABLE 1:** HR-ESI and MS/MS data of seventeen degraded products and aflatoxin B_1_.

Structure		Retention time (min)	Extract Mass (m/z)	Formula	Diff (ppm)	Loss mass	Loss formula
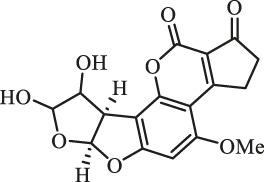		3.48	347.0758	C_17_H_15_O_8_	−2.6		
			329.0648	C_17_H_13_O_7_	−4.0	18.0110	[M + H]^+^-H_2_O
			319.0811	C_16_H_15_O_7_	−2.2	27.9947	[M + H]^+^-CO
			311.0538	C_17_H_11_O_6_	−5.8	36.0220	[M + H]^+^-H_2_O-H_2_O
			301.0697	C_16_H_13_O_6_	−5.0	46.0062	[M + H]^+^-CO-H_2_O
			283.0595	C_16_H_11_O_5_	−3.9	64.0163	[M + H]^+^-H_2_O-H_2_O-CO
			273.0747	C_15_H_13_O_5_	−5.9	74.0008	[M + H]^+^-CO-H_2_O-CO
			271.0595	C_15_H_11_O_5_	−4.1	76.0163	[M + H]^+^-CO-H_2_O-HCHO
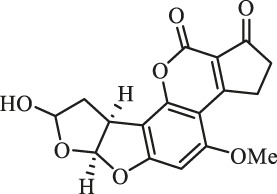		4.06	331.0801	C_17_H_15_O_7_	−5.4		
		4.18	313.0697	C_17_H_13_O_6_	−4.8	18.0104	[M + H]^+^-H_2_O
			301.0699	C_16_H_13_O_6_	−4.3	30.0102	[M + H]^+^-HCHO
			285.0746	C_16_H_13_O_5_	−6.0	46.0055	[M + H]^+^-H_2_O-CO
			283.0598	C_16_H_11_O_5_	−2.8	48.0203	[M + H]^+^-CH_2_O-H_2_O
			273.0378	C_14_H_9_O_6_	−7.7	58.0423	[M + H]^+^- CH_2_O-CO
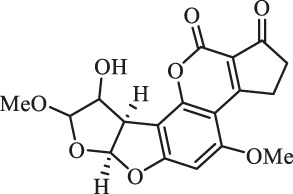		4.47	361.0905	C_18_H_17_O_8_	−5.0		
		4.82	343.0799	C_18_H_15_O_7_	−5.5	18.0106	[M + H]^+^-H_2_O
			329.0643	C_17_H_13_O_7_	−5.5	32.0262	[M + H]^+^-CH_3_OH
			315.0858	C_17_H_15_O_6_	−3.5	46.0047	[M + H]^+^-H_2_O-CO
			311.0542	C_17_H_11_O_6_	−4.5	50.0363	[M + H]^+^-H_2_O-CH_3_OH
			301.0698	C_16_H_13_O_6_	−4.7	60.0207	[M + H]^+^-CH_3_OH-CO
			283.0588	C_16_H_11_O_5_	−6.4	78.0317	[M + H]^+^-CH_3_OH-CO-H_2_O
			273.0753	C_15_H_13_O_5_	−3.7	88.0152	[M + H]^+^-CH_3_OH-CO-CO
			255.0646	C_15_H_11_O_4_	−4.3	106.0259	[M + H]^+^-CH_3_OH-CO-H_2_O-CO
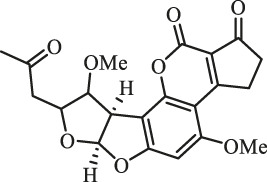		4.86	401.1224	C_21_H_21_O_8_	−3.0		
			369.0967	C_20_H_17_O_7_	−1.9	32.0257	[M + H]^+^-CH_3_OH
			343.0804	C_18_H_15_O_7_	−4.1	58.0420	[M + H]^+^-CH_3_COCH_3_
			315.0851	C_17_H_15_O_6_	−5.7	86.0373	[M + H]^+^-CH_3_COCH_3_-CO
			313.0697	C_17_H_13_O_6_	−4.8	88.0527	[M + H]^+^-CH_3_COCH_3_-CH_2_O
		6.53					[M + H]^+^-CH_3_OH-C_3_H_4_O
			287.0538	C_15_H_11_O_6_	−6.3	114.0686	[M + H]^+^-CH_3_COCH_3_-CO-CO
			285.0747	C_16_H_13_O_5_	−5.6	116.0477	[M + H]^+^-CH_3_COCH_3_-CO-CH_2_O
			283.0601	C_16_H_11_O_5_	−1.8	118.0623	[M + H]^+^-CH_3_COCH_3_-CH_2_O-CH_2_O
							[M + H]^+^-CH_3_OH-C_3_H_4_O-CH_2_O
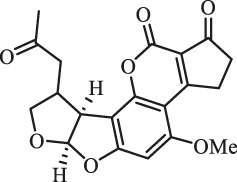		4.345.63	371.1120	C_20_H_19_O_7_	−3.0		
			313.0698	C_17_H_13_O_6_	−4.5	58.0422	[M + H]^+^-CH_3_COCH_3_
			285.0751	C_16_H_13_O_5_	−4.2	86.0369	[M + H]^+^-CH_3_COCH_3_-CO
			257.0796	C_15_H_13_O_4_	−7.0	114.0324	[M + H]^+^-CH_3_COCH_3_-CO-CO
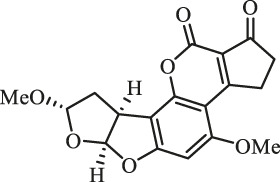		4.70	345.0971	C_18_H_17_O_7_	−0.9		
		5.40	313.0721	C_17_H_13_O_6_	2.0	32.0250	[M + H]^+^-CH_3_OH
		5.84	285.0751	C_16_H_13_O_5_	−4.2	60.0220	[M + H]^+^-CH_3_OH-CO
		5.99	269.0802	C_16_H_13_O_4_	−4.5	76.0169	[M + H]^+^-CH_3_OH-CO_2_
			257.0794	C_15_H_13_O_4_	−7.8	88.0171	[M + H]^+^-CH_3_OH-CO-CO
			243.0647	C_14_H_11_O_4_	−4.1	102.0324	[M + H]^+^-CH_3_OH-CO-CO-CH_2_
			241.0846	C_15_H_13_O_3_	−7.9	104.0125	[M + H]^+^-CH_3_OH-CO_2_-CO
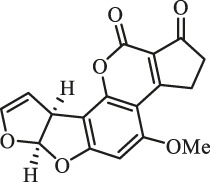		6.09	313.0705	C_17_H_13_O_6_	−2.2	27.9955	[M + H]^+^-CO
		AFB_1_	285.0750	C_16_H_13_O_5_	−4.6	43.9900	[M + H]^+^-CO_2_
			269.0805	C_16_H_13_O_4_	−3.3	55.9912	[M + H]^+^-CO-CO
			257.0793	C_15_H_13_O_4_	−8.2	72.0211	[M + H]^+^-CO_2_-CO
			241.0494	C_14_H_9_O_4_	−2.9	83.9857	[M + H]^+^-CO-CO-CO
			229.0848	C_14_H_13_O_3_	−1.7		
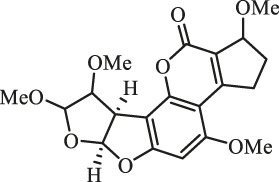		6.41	391.1380	C_20_H_23_O_8_	−3.3		
			359.1111	C_19_H_19_O_7_	−5.6	32.0269	[M + H]^+^-CH_3_OH
			345.0955	C_18_H_17_O_7_	−5.5	46.0425	[M + H]^+^-CH_3_OH-CH_2_
			327.0852	C_18_H_15_O_6_	−5.2	64.0528	[M + H]^+^-CH_3_OH-CH_3_OH
			313.0696	C_17_H_13_O_6_	−5.1	78.0684	[M + H]^+^-CH_3_OH-CH_2_-CH_3_OH
			297.0749	C_17_H_13_O_5_	−4.7	94.0631	[M + H]^+^-CH_3_OH-CH_3_OH-CH_2_O
			285.0754	C_16_H_13_O_5_	−3.2	106.0626	[M + H]^+^-CH_3_OH-CH_2_-CH_3_OH-CO
			283.0597	C_16_H_11_O_5_	−3.2	108.0783	[M + H]^+^-CH_3_OH-CH_3_OH-CH_2_O-CH_2_
			255.0653	C_15_H_11_O_4_	−1.6	136.0727	[M + H]^+^-CH_3_OH-CH_2_-CH_3_OH-CO
							[M + H]^+^-CH_3_OH-CH_3_OH-CH_2_O-CH_2_-CO
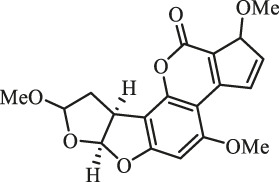		4.93	359.1121	C_19_H_19_O_7_	−2.8		
		7.30	345.0957	C_18_H_17_O_7_	−4.9	14.0164	[M + H]^+^-CH_2_
			327.0856	C_18_H_15_O_6_	−4.0	32.0265	[M + H]^+^-CH_3_OH
			313.0693	C_17_H_13_O_6_	−6.1	46.0428	[M + H]^+^-CH_2_-CH_3_OH
			301.0697	C_16_H_13_O_6_	−5.0	58.0424	[M + H]^+^-CH_3_OH-C_2_H_2_
			299.0904	C_17_H_15_O_5_	−5.0	60.0217	[M + H]^+^-CH_3_OH-CO
			287.0564	C_15_H_11_O_6_	2.8	72.0557	[M + H]^+^-CH_3_OH-C_2_H_2_-CH_2_
			273.0746	C_15_H_13_O_5_	−6.2	86.0375	[M + H]^+^-CH_3_OH-C_2_H_2_-CO
			259.0594	C_14_H_11_O_5_	−4.6	100.0527	[M + H]^+^-CH_3_COCH_3_-CO-CO

### Structural Analysis of Degraded Products Based on Exact Molecular Weights and Fragment Ions

**FIGURE 3:**
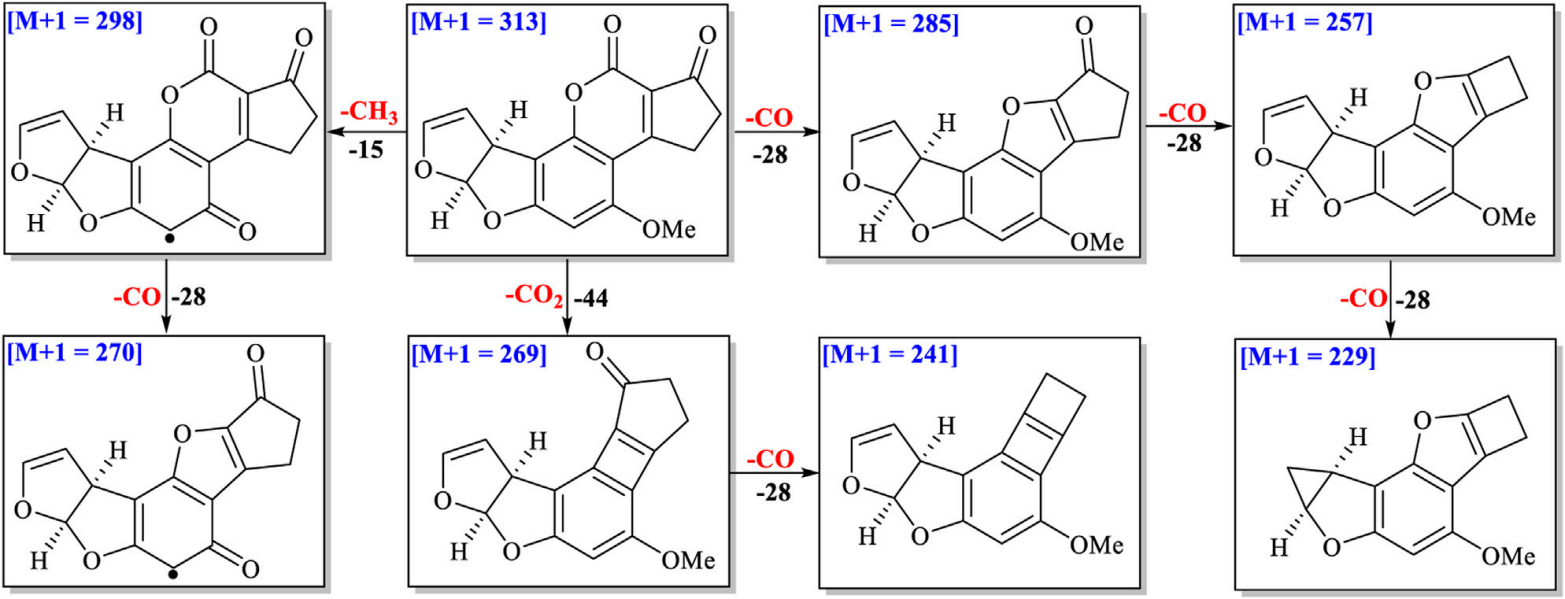
Fragmentation pathway of AFB_1_.

Different free radicals such as reactive hydroxyl (OH^•^), hydrated electrons (eaq^−^), hydrogen atoms (H^•^), and methoxy species (OCH_3_^•^) were produced when methanol–H_2_O and acetone–H_2_O solvents were irradiated under UV (White, 2001; Azrague et al., 2005). These free radicals could attack the AFB_1_ structure to form different degraded products. The double bond C_8_-C_9_ in AFB_1_ was broken easily by these free radicals *via* addition reactions. Ten and seven main degraded products in methanol–H_2_O and acetone–H_2_O solvents were characterized based on molecular weights and fragment ions of compounds (Supplementary Tables S1, S2). The other degraded product fragmentation rules are provided in supporting information, considering a similar fragmentation pathway with AFB_1_ (Figure 3 and Supplementary Figures S2–S9).

Four ions as (stereo) isomers (*m*/*z* 345, C_18_H_16_O_7_) appeared at *t*_R_ = 4.70, 5.40, 5.84 and 5.99 min in methanol–H_2_O solvent with 32 Da (CH_4_O) more than that of AFB_1_ (Supplementary Figure S2). After a neutral loss of CH_3_OH from ion (*m*/*z* 345), the fragmentation pathways of these four ions were nearly the same as those of AFB_1_. It is suggested that these four degraded compounds might be addition products of CH_3_OH with AFB_1_ at C-8/C-9. The possible fragmentation pathways of these four (stereo) isomers are depicted in Supplementary Figure S2.

The molecular formula of the ion at *m*/*z* 361 ([M+1], *t*_R_ = 4.47 and 4.82 min) was determined to be C_18_H_16_O_8_ based on HR-ESI-MS with 16 Da (O) more than that of degraded products (*m*/*z* 345) (Supplementary Figure S3), suggesting one more oxygen atom connected on C_8_ or C_9_. Both of them were suggested to be the addition products from free radical hydrogen atoms (OH^•^) and methoxy species (OCH_3_^•^) with C_8_/C_9_ or C_9_/C_8_ of AFB_1_ under UV irradiation. The possible fragmentation pathway of the ion (*m*/*z* 361) is shown in Supplementary Figure S3.

Three ions at *m*/*z* 359 ([M+1], *t*_R_ = 4.94, 7.08 and 7.30 min) gave the molecular formula as C_19_H_18_O_7_ based on HR-ESI-MS. The neutral loss of -CO, -CH_3_OH, and -C_2_H_2_ was observed in the MS/MS profiles (Supplementary Figure S4). The possible structure and fragmentation pathway of these three ions is suggested in Supplementary Figure S4.

The molecular formula of the degraded product at *m*/*z* 391 ([M+1], *t*_R_ = 6.41 min) was determined to be C_20_H_22_O_8_ based on HR-ESI-MS (Supplementary Figure S5). Sequential losses of two -CH_3_OH (391→359→327), one -CH_2_O (327→297), and one -CH_2_ (297→283) implied that four methoxyls might be present in degraded products. Two methoxyls might be connected on C-8/C-9 and the keto-carboxyl group might be transformed to another methoxyl through reduction and addition reactions, and the remaining -OMe was anchored on the aromatic ring. The possible fragmentation pathway of these three ions is suggested in Supplementary Figure S5.

The molecular formula of ions at *m*/*z* 331 ([M+1, C_17_H_14_O_7_] in acetone–H_2_O at 4.06 and 4.18 min) possessed 18 Da (H_2_O) more than that of AFB_1_, which indicated these two degraded products were (stereo) isomers (Supplementary Figure S6). The fragmentation pathways of these two ions were nearly the same as those of AFB_1_ after the loss of a molecule of H_2_O, which implied that two degraded products were the adducts of H_2_O with the double bond C-8/C-9. Though the molecular formulas and fragmentation pathways of these two degraded products were the same, the retention time and abundance of fragment ions were different. A higher abundance of ion at *m*/*z* 313 (*t*_R_ = 4.18 min) was observed than the other (*t*_R_ = 4.06 min). This suggested that the position of OH on the furan ring was different in two degraded products. The possible fragmentation pathway of two ions is suggested in Supplementary Figure S6.

The molecular formula of the ion at *m*/*z* 347 ([M+1], *t*_R_ = 3.48 min) was determined to be C_18_H_14_O_8_ based on HR-ESI-MS with 16 Da (O) more than that of degraded products (*m*/*z* 331), indicating two hydroxyl groups connected on C_8_ and C_9_, respectively (Supplementary Figure S7). The possible fragmentation pathway is suggested in Supplementary Figure S7.

The molecular formula of the ion at *m*/*z* 371 ([M+1], *t*_R_ = 4.34 and 5.63 min) was determined to be C_20_H_18_O_7_ based on HR-ESI-MS with 58 Da (CH_3_COCH_3_) more than that of AFB_1_ (*m*/*z* 313) (Supplementary Figure S8), which implied that one molecule of acetone attacked on C-8 or C-9 under UV irradiation. The possible fragmentation pathway of them is suggested in Supplementary Figure S8.

The molecular formulas of ions at *m*/*z* 401 ([M+1], *t*_R_ = 4.86 and 6.54 min) were determined to be C_21_H_20_O_8_ based on HR-ESI-MS. The loss of 32 Da from *m*/*z* 401 to *m*/*z* 369 and the loss of 58 Da from *m*/*z* 401 to *m*/*z* 343 suggested that a methoxyl and acetone were connected on C-8/C-9 or C-9/C-8 (Supplementary Figure S9). The possible fragmentation pathway of these two ions is suggested in Supplementary Figure S9.

Though seventeen degraded products were characterized by molecular formula and fragment ions, the planar structures and configurations of some degraded products could not be determined only based on UPLC-Q-TOF-MS/MS analysis. Thus, further purification and NMR experiments are needed to elucidate their structures and stereochemistry.

### Purification and Elucidation of Seven Degraded Products Structures

**FIGURE 4:**
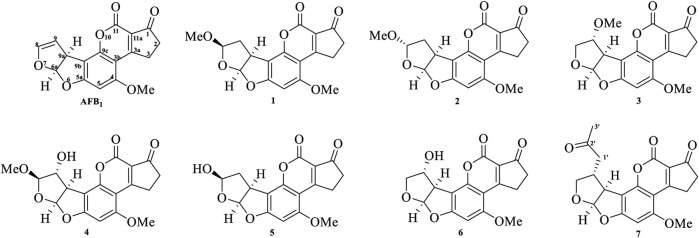
Structures of AFB_1_ and 1-7

**FIGURE 5:**
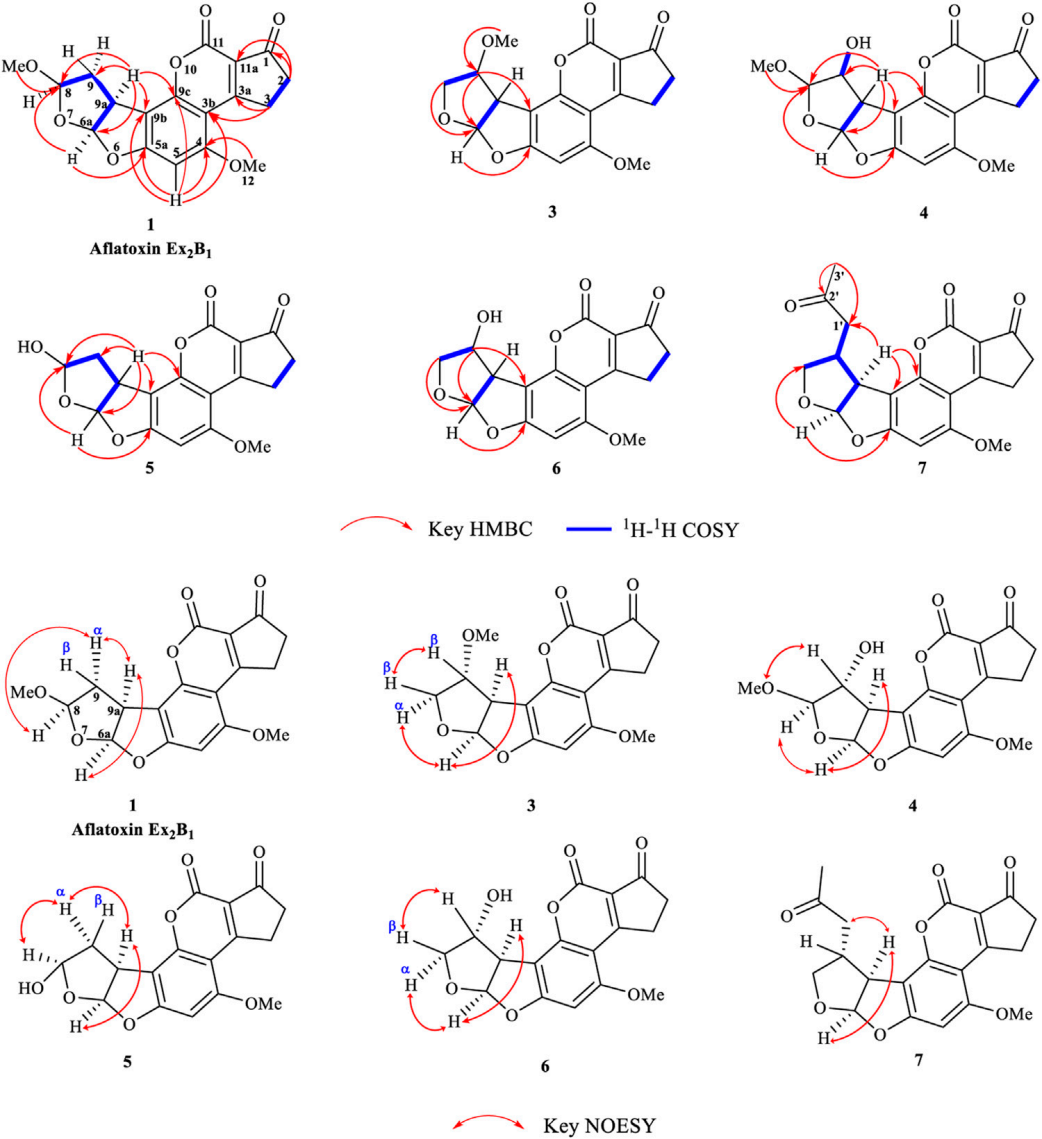
Key 2D-NMR and NOESY correlations of 1 and 3-7

Seven main degraded products with limited amounts were purified by HPLC and then elucidated by NMR spectra (Figure 4). Compounds 1–3 were isolated as the photochemical adducts of 6-methoxydifurocoumarone, which were analyzed based on the ^1^H-NMR spectrum (Waiss and Wiley, 1969). In this study, the structures of these three degraded products were elucidated in detail by analyzing ^1^H, ^13^C, and 2D-NMR spectra (Figure 5). The ^1^H-NMR data of 1–3 and ^13^C-NMR data of 1 and 3 are shown in Table 2 and Table 3. The relative configurations of 1 and 3 were determined by NOESY correlations (Figure 5). Compound 4 was a new degraded product isolated from methanol solution. The molecular formula of 4 was determined to be C_18_H_17_O_8_ on the basis of HR-ESI-MS with 16 more daltons than that of 1, implying that an additional hydroxyl group was present in 4, which was supported by the NMR spectra (Table 2 and Table 3). The ^1^H–^1^H COSY and HMBC correlations confirmed that the additional hydroxyl group was connected on C-9 (Figure 5). The NOESY correlations determined the relative configuration of 8-OMe and 9-OH to be β and α configuration, respectively (Figure 5).

**TABLE 2:** ^1^H NMR data of compounds **1**-**4** in acetone-*d*_6_ at 600 MHz and **5**-**7** in pyridine-*d*_5_ at 500 MHz.

**Pos**	**1**	**2**	**3**	**4**	**5**	**6**	**7**
	***δ***_**H**_ **(*J* in Hz)**	***δ***_**H**_ **(*J* in Hz)**	***δ***_**H**_ **(*J* in Hz)**	***δ***_**H**_ **(*J* in Hz)**	***δ***_**H**_ **(*J* in Hz)**	***δ***_**H**_ **(*J* in Hz)**	***δ***_**H**_ **(*J* in Hz)**
2	2.51, t (5.4)	2.48, m	2.48, m	2.47, dd (6.0, 4.8)	2.53, ddd (7.0, 4.5, 2.5)	2.57, t (5.5)	2.57, dt (6.5, 5.0)
3	3.42, dt (5.4, 4.2)	3.38, m	3.38, m	3.38, ddd (5.4, 4.2, 3.0)	3.03, m	3.14, m	3.13, m
5	6.54, s	6.54, s	6.52, s	6.50, s	6.54, s	6.59, s	6.56, s
6a	6.60, d (6.0)	6.48, d (6.0)	6.57, d (5.4)	6.65, d (6.0)	6.79, d (6.0)	6.95, d (5.5)	6.74, d (5.5)
8	5.27, d (4.8)	5.15, t (4.8)	4.10, dd (10.8, 1.2)	5.02, s	6.06, d (5.0)	4.44, d (10.0)	4.04, m
			3.66, dd (10.8, 3.0)			4.06, dd (10.0, 3.0)	
9	2.42, ddd (13.2, 9.6, 4.8)	2.31, m	4.12, d (3.0)	4.37, d (3.6)	2.68, d (13.0)	5.02, d (3.0)	3.13, m
	2.27, d (13.2)	2.23, m			2.36, m		
9a	4.19, dd (9.6, 6.0)	4.24, t (6.0)	4.15, d (5.4)	3.95, d (6.0)	4.20, dd (9.5, 6.0)	4.37, d (5.5)	3.93, dd (5.5, 1.0)
4-OCH_3_	4.03, s	4.01, s	4.00, s	3.99, s	3.72, s	3.84, s	3.84, s
8-OCH_3_	3.16, s	3.37, s		3.11, s			
9-OCH_3_			3.43, s				
1′							2.72, d (7.5)
3′							2.12, s

**TABLE 3:** ^13^C NMR data of compounds **1**, **3**, **4** in acetone-*d*_6_ and **5**-**7** in pyridine-*d*_5_ at 125 MHz.

**Pos**	**1**	**3**	**4**	**5**	**6**	**7**
1	200.9, C	200.8, C	200.8, C	200.8, C	200.8, C	200.8, C
2	35.5, CH_2_	35.5, CH_2_	35.5, CH_2_	35.8, CH_2_	35.8, CH_2_	36.0, CH_2_
3	29.5, CH_2_	29.6, CH_2_	29.8, CH_2_	29.5, CH_2_	29.6, CH_2_	29.1, CH_2_
3a	178.1, C	177.9, C	178.1, C	177.7, C	177.7, C	178.3, C
3b	103.8, C	104.2, C	104.1, C	104.4, C	104.3, C	103.2, C
4	162.7, C	163.0, C	162.9, C	162.4, C	162.6, C	163.1, C
5	91.2, CH	91.0, CH	91.2, CH	92.2, CH	90.7, CH	90.3, C
5a	167.0, C	167.8, C	167.0, C	167.6, C	168.6, C	167.4, C
6a	115.0, CH	114.7, CH	115.0, CH	115.4, CH	115.0, CH	114.3, C
8	107.6, CH	72.6, CH_2_	112.5, CH	101.7, CH	76.9, CH_2_	73.3, CH_2_
9	37.8, CH_2_	84.7, CH	78.2, CH	38.9, CH_2_	74.7, CH	41.1, CH
9a	43.1, CH	50.7, CH	52.8, CH	43.4, CH	55.2, CH	50.3, CH
9b	109.8, C	107.6, C	106.3, C	110.1, C	105.1, C	107.2, C
9c	153.5, C	154.5, C	153.8, C	154.5, C	154.5, C	154.2, C
11	155.3, C	156.0, C	154.8, C	155.6, C	155.6, C	156.5, C
11a	117.8, C	117.0, C	117.5, C	117.5, C	117.8, C	117.7, C
1′						47.5, CH_2_
2′						206.5, C
3′						30.4, CH_3_
4-OCH_3_	56.9, CH_3_	57.1, CH_3_	57.1, CH_3_	56.7, CH_3_	57.8, CH_3_	56.8, CH_3_
8-OCH_3_	55.0, CH_3_		54.8, CH_3_			
9-OCH_3_		56.8, CH_3_				

Compounds 5 and 6 were obtained as an inseparable mixture through HPLC with various stationary and mobile phases, whereas well-resolved NMR spectra determined the structures of 5 and 6 as isomers. The ^1^H and ^13^C spectra data of 5 were reported, and 6 was a new degraded product reported for the first time (Cox and Cole, 1977; Liu, Jin, Tao, Shan, et al., 2010; Wang et al., 2011; Wang et al., 2012; Stanley et al., 2020). The molecular formula of 5 and 6 was determined to be C_17_H_14_O_7_ on the basis of HR-ESI-MS, with 18 more daltons than that of AFB_1_, implying that 5 and 6 might be transformed from AFB_1_ through an addition reaction with H_2_O on the double bond (C-8/C-9). The planar and relative configurations of 5 and 6 were established based on 2D-NMR data (Figure 5). Compound 7 was a new degraded product isolated from acetone solvent. The molecular formula of 7 was established to be C_20_H_19_O_7_ based on HR-ESI-MS. In the ^1^H NMR spectrum, an additional methyl (*δ*_H_ = 2.12 ppm) and an additional methylene unit (*δ*_H_ = 2.72 ppm) were observed compared with that of AFB_1_, which indicated that one molecule of acetone might be connected on C-8 or C-9. The ^1^H–^1^H COSY and HMBC correlations confirmed that the acetonyl group was connected with C-9 (Figure 5). The NOESY correlations from H-9a (*δ*_H_ = 3.93 ppm) to H-1' (*δ*_H_ = 2.72 ppm) determined the acetonyl group to be α-configuration (Figure 5). Considering that the stereochemistry of C-6a and C-9a were not changed in the photocatalytic reaction, the absolute configurations of (**1**–**7**) are shown in Figure 4.

### Elucidation of the Photodegraded Mechanism of Degraded Products

**FIGURE 6:**
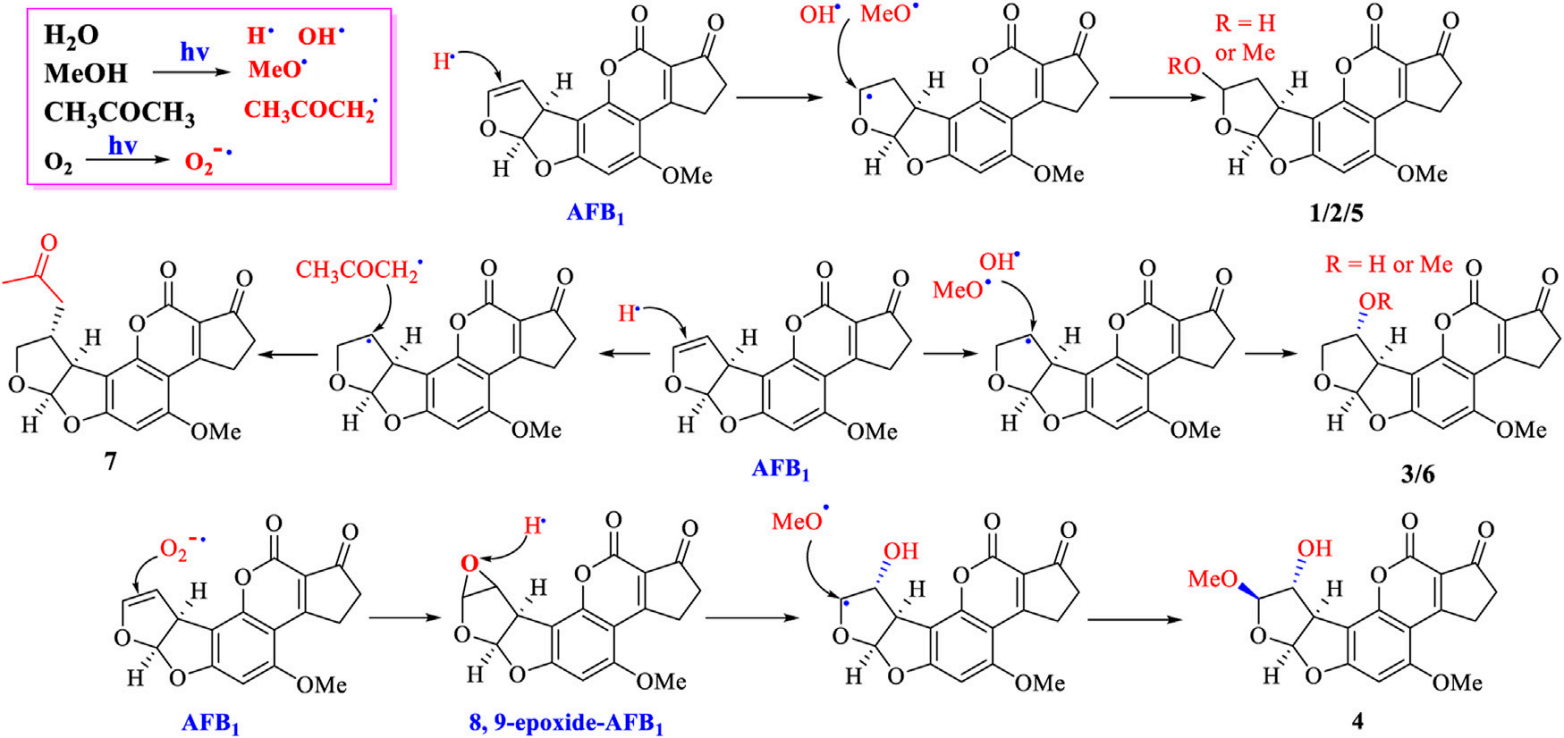
Possibly photocatalytic mechanism of AFB_1_ in MeOH and acetone.

According to the structural features of AFB_1_ and degraded products (1–7), the possible photocatalytic reactions were suggested: 1) addition reactions happened between MeOH, H_2_O, or acetone with AFB_1_ under UV irradiation to produce compounds such as 1–3 and 5–7; 2) compound 4 might be originated from the oxygen free radical attacking the double bond (C-8/C-9) to form an epoxide, which was further attacked by OMe^•^ or OH^•^ (Figure 6) (Waiss and Wiley, 1969; Iyer et al., 1994). The photocatalytic mechanism was suggested: MeOH, H_2_O, or CH_3_COCH_3_ formed potential free radicals (H^•^, OH^•^, OMe^•^, or CH_3_COCH_2_^•^) under UV irradiation. Then, H^•^ attacked on the double bond (C-8 or C-9) leading to form carbon-free radicals, which was then coupled with OH^•^, OMe^•^, or CH_3_COCH_2_^•^ to shape degraded products 1–3 and 5–7 (Waiss and Wiley, 1969; Iyer et al., 1994; Jamil et al., 2017; Mao et al., 2019). In addition, O_2_ in the air under UV irradiation could form O_2_^•-^, which could attack on the double bond C-8/C-9 to produce 8,9-epoxide-AFB_1_. Addition reactions then happened fast by the highly unstable intermediate 8,9-epoxide-AFB_1_ with OMe^•^ to form the degraded products of 4 (Figure 6) (Waiss and Wiley, 1969; Iyer et al., 1994; Jamil et al., 2017).

**FIGURE 7:**
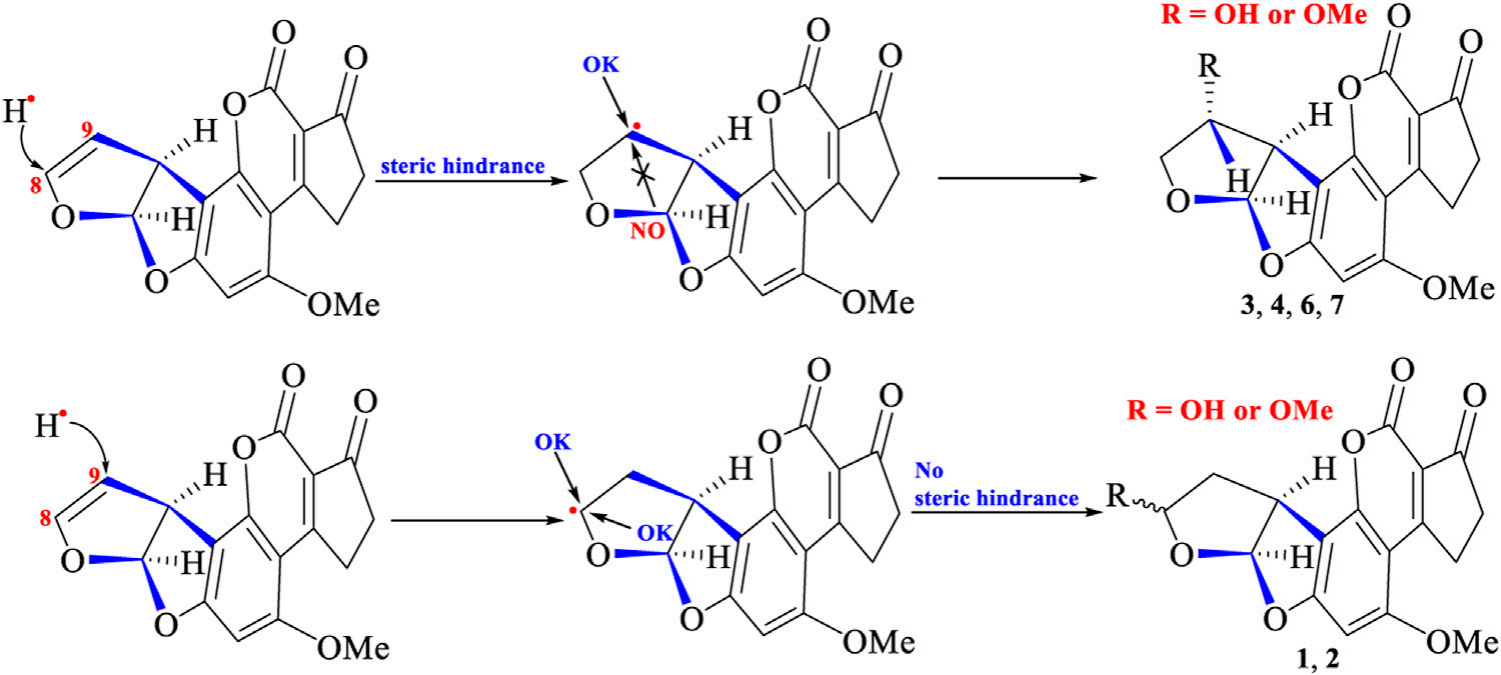
Possible catalytic reaction happened at C-8 and C-9

From the structural features of degraded products (1–7), an interesting phenomenon was also observed that the group of C-9 (in 3, 4, 6, and 7) was α-configuration, whereas the group of C-8 in 1 and 2 was α- or β-configuration. This demonstrated that steric hindrance (from right part of AFB_1_ structure) might exist and prevent different groups (OH^•^, OMe^•^, or CH_3_COCH_2_^•^) attacking C-9 from the positive face (β-position), whereas C-8 could be attacked from two sides (α- or β-configuration) without steric hindrance. The crystal structure of AFB_1_ (Cheung and Sim, 1964; van Soest and Peerdeman, 1970) revealed that the right part of the AFB_1_ structure was indeed closer to C-9 than C-8 in space, which might preclude different groups to attack C-9 from the positive face (β-position) due to spatial hindrance. The photocatalytic reactions are depicted in Figure 7.

### Toxic Evaluation of Degraded Products

The cytotoxicity of AFB_1_ and seven degraded products (1–7) was evaluated against human normal hepatocytes LO-2 and cancer cell lines Hep-G2 and MCF-7 using the MTS method, with *cis*-platinum as the positive control; the results are shown in Table 4. AFB_1_ displayed stronger cytotoxicity to three cell lines than the degraded products, further supporting that the double bond (C-8/C-9) in the furan ring was the key toxic group, and the toxicity was markedly reduced after the double bond was broken.

**TABLE 4:** Cytotoxic activity of seven degraded products and aflatoxin B_1_.

**Compounds**	**Cytotoxic activity (μM)**		
	**LO-2**	**Hep-G2**	**MCF-7**
AFB_1_	22.47 ± 3.10	29.08 ± 4.92	36.57 ± 4.43
1	>100	>100	>100
2	>100	>100	>100
3	>100	>100	>100
4	>100	>100	>100
5/6	>100	>100	>100
7	>100	>100	>100
*cis*-platinum	6.54 ± 0.72	11.36 ± 1.47	21.47 ± 2.18

## Conclusion

In this work, the degraded products of AFB_1_ under UV irradiation were analyzed through UPLC-Q-TOF-MS/MS, and seventeen degraded products were characterized. Seven degraded products were purified and elucidated by NMR experiments. The double bond (C-8/C-9) of all degraded products was broken, which was coupled with different groups such as OH^•^, H^•^, and OCH_3_^•^ through addition reactions under UV irradiation. The cytotoxic evaluation revealed that the toxicity of AFB_1_-degraded products was markedly reduced after their double bond in the furan ring was cleaved. The results demonstrated that the UPLC-Q-TOF-MS/MS technique coupled with purification NMR analysis and biological tests was an applicably integrated approach for the analysis, characterization, and toxic evaluation of degraded products of AFB_1_, which can also be used to evaluate other mycotoxin degradation processes.

## Data Availability

The datasets presented in this study can be found in online repositories. The names of the repository/repositories and accession number(s) can be found in the article/Supplementary Material.
