# Supplementary material for: Accurate Identification of Degraded Products of Aflatoxin B1 Under UV Irradiation Based on UPLC-Q-TOF-MS/MS and NMR Analysis
Source: Front Chem. 2021 Nov 24;9:789249. doi: 10.3389/fchem.2021.789249 (PMC8654243; doi:10.3389/fchem.2021.789249)
Supplement: Supplementary file 1 [file DataSheet1.pdf]

Supporting information

Accurate identification of degraded products of Aflatoxin B_1 under UV Irradiation based on UPLC-Q-TOF-MS/MS and NMR analysis

Yan-Duo Wang[1,†], Cheng-Gang Song[2,†], Jian Yang[3], Tao Zhou[4], Yu-Yang Zhao[3], Jian-Chun Qin[2,*], Lan-Ping Guo[3,*], Gang Ding[1,*]

[1]*Key Laboratory of Bioactive Substances and Resources Utilization of Chinese Herbal Medicine, Ministry of Education, Institute of Medicinal Plant Development, Chinese Academy of Medical Sciences and Peking Union Medical College, Beijing 100193, People's Republic of China.*

[2]*College of Plant Sciences, Jilin University, Changchun, Jilin 130062, People's Republic of China.*

[3]*State Key Laboratory Breeding Base of Dao-di Herbs, National Resource Center for Chinese Materia Medica, China Academy of Chinese Medical Sciences, Beijing 100700, People's Republic of China.*

[4]*Guizhou University of Traditional Chinese Medicine, Guiyang, Guizhou 550025, People's Republic of China.*

[†]These authors have contributed equally to this work and share the first authorship.

Table of contents

Table S1 Mass accuracy measurement of 10 degraded products of aflatoxin B_1 in methanol solvent, using UPLC-Q-TOF-MS/MS.

Table S2 Mass accuracy measurement of 7 degraded products of aflatoxin B_1 in acetone solvent, using UPLC-Q-TOF-MS/MS

Figure S1 Analysis of AFB_1 based on HR-ESI and MS/MS data at *m/z* 313, t_R = 6.09 min.

Figure S2 Analysis of degraded products structures based on HR-ESI and MS/MS data at *m/z* 345, t_R = 4.70 min, 5.40 min, 5.84 min and 5.99 min.

Figure S3 Analysis of degraded products structures based on HR-ESI and MS/MS data at *m/z* 361, t_R = 4.47 min and 4.82 min.

Figure S4 Analysis of degraded products structures based on HR-ESI and MS/MS data at *m/z* 359, t_R = 4.94 min, 7.08 min and 7.30 min.

Figure S5 Analysis of degraded products structures based on HR-ESI and MS/MS data at *m/z* 391, t_R = 6.41 min.

Figure S6 Analysis of degraded products structures based on HR-ESI and MS/MS data at *m/z* 331, t_R = 4.06 min and 4.18 min.

Figure S7 Analysis of degraded products structures based on HR-ESI and MS/MS data at *m/z* 347, t_R = 3.48 min.

F**igure S8** Analysis of degraded products structures based on HR-ESI and MS/MS data at *m/z* 371, t_R = 4.34 min and 5.63 min.

Figure S9 Analysis of degraded products structures based on HR-ESI and MS/MS data at *m/z* 401, t_R = 4.86 min and 6.54 min.

Figure S10 ^{1}H-NMR spectrum of compound **1** (Acetone-d_6, 600MHz)

Figure S11 (a-c) ^{13}C-NMR spectrum of compound **1** (Acetone-d_6, 125 MHz)

Figure S12 ^{1}H-^{1}H COSY spectrum of compound **1** (Acetone-d_6, 500 MHz)

Figure S13 HSQC spectrum of compound **1** (Acetone-d_6, 500 MHz)

Figure S14 HMBC spectrum of compound **1** (Acetone-d_6, 500 MHz)

Figure S15 ROESY spectrum of compound **1** (Acetone-d_6, 500 MHz)

Figure S16 HR-ESI-MS spectrum of compound **1**

Figure S17 ^{1}H-NMR spectrum of compound **2** (Acetone-d_6, 600MHz)

Figure S18 HR-ESI-MS spectrum of compound **2**

Figure S19 ^{1}H-NMR spectrum of compound **3** (Acetone-d_6, 600 MHz)

Figure S20 (a-c) ^{13}C-NMR spectrum of compound **3** (Acetone-d_6, 125 MHz)

Figure S21 ^{1}H-^{1}H COSY spectrum of compound **3** (Acetone-d_6, 500 MHz)

Figure S22 HSQC spectrum of compound **3** (Acetone-d_6, 500 MHz)
Figure S23 HMBC spectrum of compound **3** (Acetone-d_6, 500 MHz)
Figure S24 ROESY spectrum of compound **3** (Acetone-d_6, 500 MHz)
Figure S25 HR-ESI-MS spectrum of compound **3**
Figure S26 ^{1}H-NMR spectrum of compound **4** (Acetone-d_6, 600 MHz)
Figure S27 (a-c) ^{13}C-NMR spectrum of compound **4** (Acetone-d_6, 125 MHz)
Figure S28 ^{1}H-^{1}HCOSY spectrum of compound **4** (Acetone-d_6, 500 MHz)
Figure S29 HSQC spectrum of compound **4** (Acetone-d_6, 500 MHz)
Figure S30 HMBC spectrum of compound **4** (Acetone-d_6, 500 MHz)
Figure S31 ROESY spectrum of compound **4** (Acetone-d_6, 500 MHz)
Figure S32 HR-ESI-MS spectrum of compound **4**
Figure S33 ^{1}H-NMR spectrum of compound **5** and **6** (Pyridine-d_5, 500MHz)
Figure S34 ^{1}H-NMR spectrum of compound **6** (CD_3OD, 500 MHz)
Figure S35 (a, b) ^{13}C-NMR spectrum of compound **5** and **6** (Pyridine-d_5, 125 MHz)
Figure S36 ^{1}H-^{1}HCOSY spectrum of compound **5** and **6** (Pyridine-d_5, 500 MHz)
Figure S37 HSQC spectrum of compound **5** and **6** (Pyridine-d_5, 500 MHz)
Figure S38 HMBC spectrum of compound **5** and **6** (Pyridine-d_5, 500 MHz)
Figure S39 ROESY spectrum of compound **5** and **6** (Pyridine-d_5, 500 MHz)
Figure S40 HR-ESI-MS spectrum of compound **5**
Figure S41 HR-ESI-MS spectrum of compound **6**
Figure S42 ^{1}H-NMR spectrum of compound **7** (Pyridine-d_5, 500MHz)
Figure S43 ^{13}C-NMR spectrum of compound **7** (Pyridine-d_5, 125 MHz)
Figure S44 ^{1}H-^{1}HCOSY spectrum of compound **7** (Pyridine-d_5, 500 MHz)
Figure S45 HSQC spectrum of compound **7** (Pyridine-d_5, 500 MHz)
Figure S46 HMBC spectrum of compound **7** (Pyridine-d_5, 500 MHz)
Figure S47 ROESY spectrum of compound **7** (Pyridine-d_5, 500 MHz)
Figure S48 HR-ESI-MS spectrum of compound **7**

Table S1 Mass accuracy measurement of 10 degraded products of aflatoxin B_1 in methanol solvent, using UPLC-Q-TOF-MS/MS.

Retention time (min)	Experimental mass (m/z)	Theoretical mass (m/z)	Elemental composition	Error		DBE	Scores (%)
				mDa	ppm		
4.47	361.0912	361.0923	$C_{18}H_{17}O_8$	-1.1	-3.0	11	100
4.70	345.0960	345.0974	$C_{18}H_{17}O_7$	-1.4	-4.1	11	98.74
4.82	361.0905	361.0923	$C_{18}H_{17}O_8$	-1.8	-5.0	11	99.29
4.93	359.1118	359.1131	$C_{19}H_{19}O_7$	-1.3	-3.6	11	97.72
5.40	345.0963	345.0974	$C_{18}H_{17}O_7$	-1.1	-3.2	11	99.70
5.84	345.0971	345.0974	$C_{18}H_{17}O_7$	-0.3	-0.9	11	100
5.99	345.0963	345.0974	$C_{18}H_{17}O_7$	-1.1	-3.2	11	86.77
6.41	391.1380	391.1393	$C_{20}H_{23}O_8$	-1.3	-3.3	10	99.18
7.08	359.1127	359.1131	$C_{19}H_{19}O_7$	-0.4	-1.1	11	100
7.30	359.1121	359.1131	$C_{19}H_{19}O_7$	-1.0	-2.8	11	98.97

Table S2 Mass accuracy measurement of 7 degraded products of aflatoxin B_1 in acetone solvent, using UPLC-Q-TOF-MS/MS.

Retention time (min)	Experimental mass (*m/z*)	Theoretical mass (*m/z*)	Elemental composition	Error		DBE	Scores (%)
				mDa	ppm		
3.48	347.0758	347.0767	$C_{17}H_{15}O_8$	-0.9	-2.6	11	99.13
4.06	331.0805	331.0818	$C_{17}H_{15}O_7$	-1.3	-3.9	11	54.98
4.18	331.0801	331.0818	$C_{17}H_{15}O_7$	-1.7	-5.1	11	79.61
4.34	371.1112	371.1131	$C_{20}H_{19}O_7$	-1.9	-5.1	12	98.58
4.86	401.1224	401.1236	$C_{21}H_{21}O_8$	-1.2	-3.0	12	100
5.63	371.1121	371.1131	$C_{20}H_{19}O_7$	-1.0	-2.7	12	99.33
6.53	401.1221	401.1236	$C_{21}H_{21}O_8$	-1.5	-3.7	12	100

Figure S1 Analysis of AFB_1 based on HR-ESI and MS/MS data at *m/z* 313, t_R = 6.09 min.

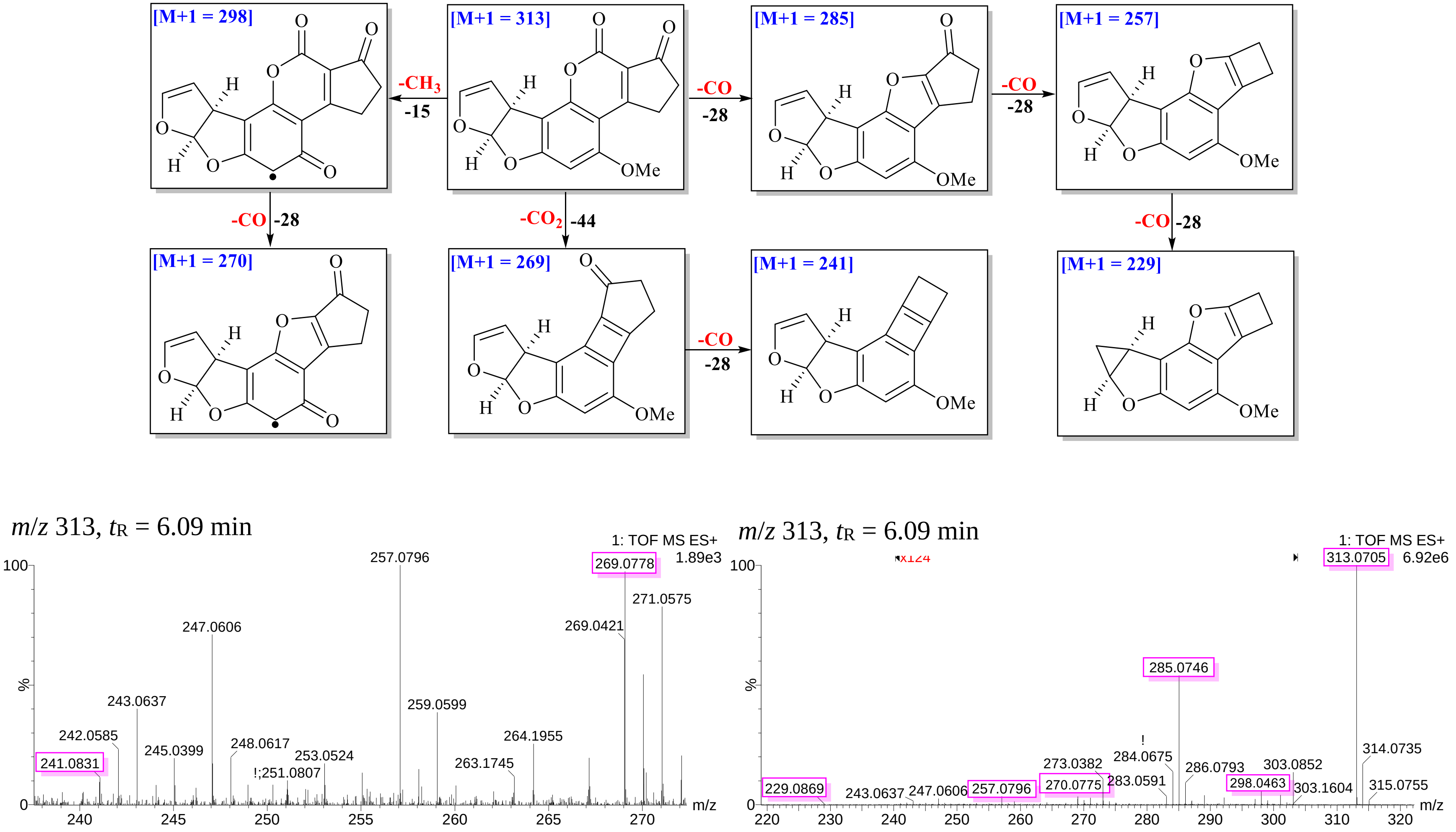

Figure S2 Analysis of degraded products structures based on HR-ESI and MS/MS data at *m/z* 345, t_R = 4.70 min, 5.40 min, 5.84 min and 5.99 min.

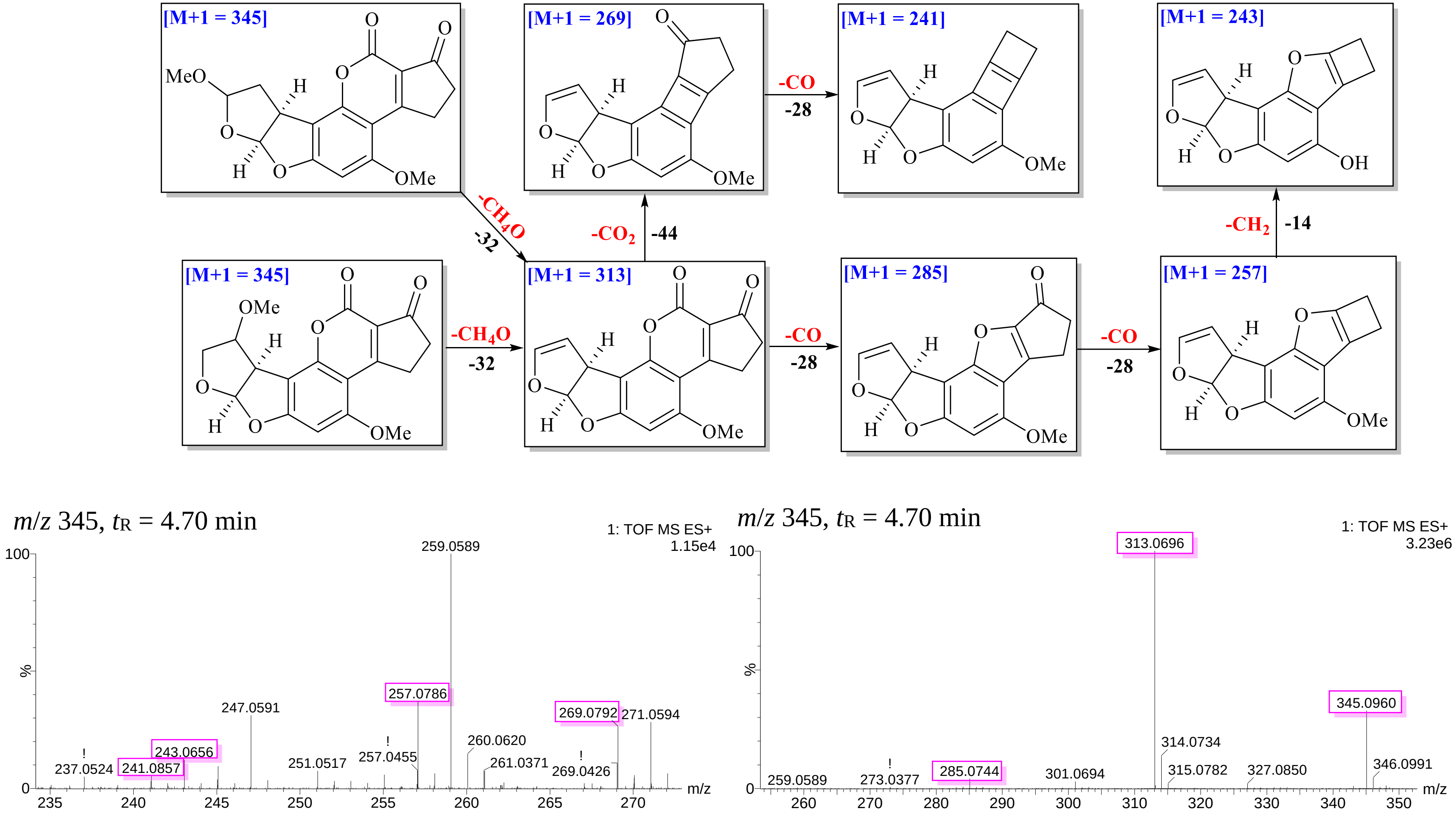

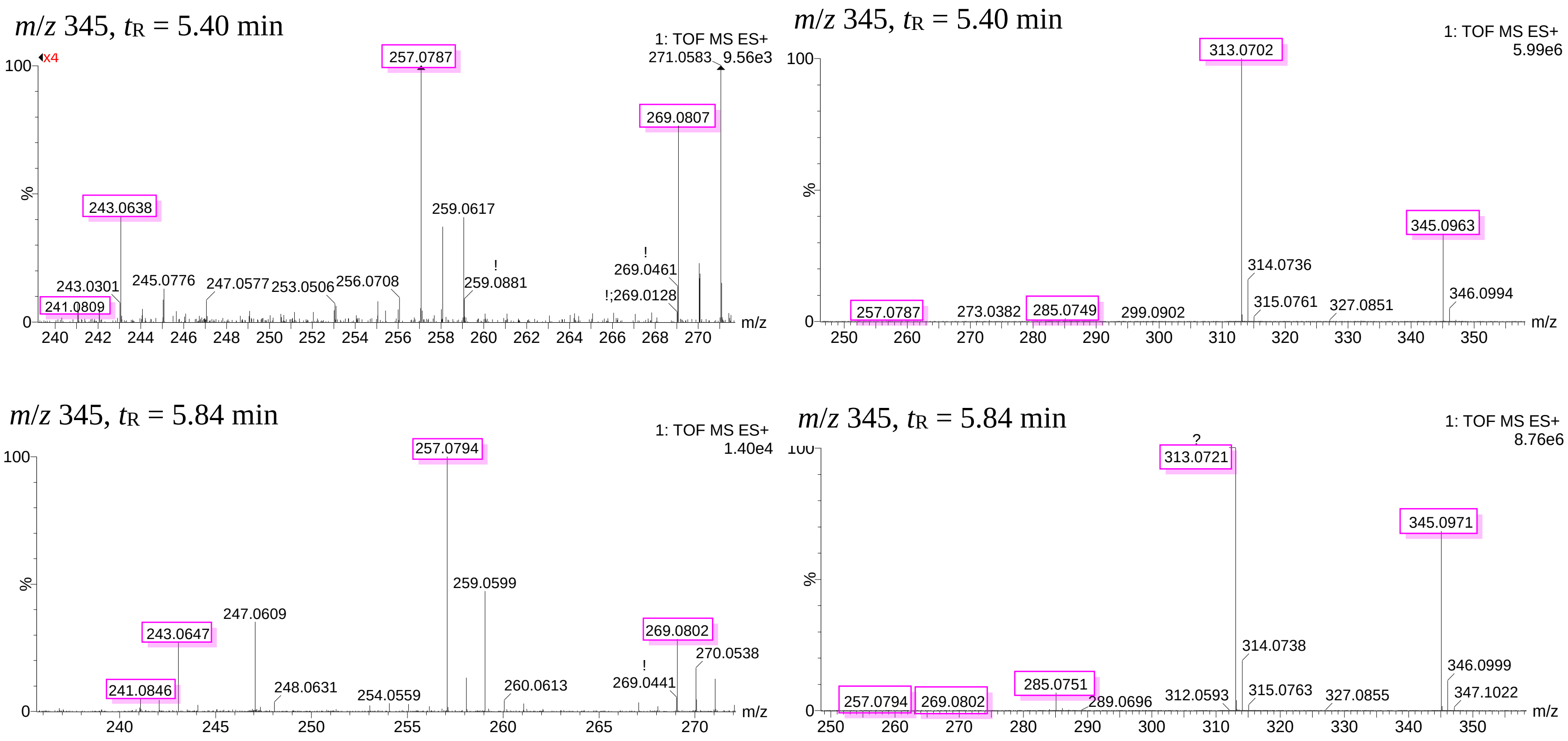

m/z 345, tR = 5.40 min
1: TOF MS ES+
271.0583 9.56e3
x4
100
%
0
257.0787
269.0807
243.0638
259.0617
259.0881
269.0461
!;269.0128
243.0301
241.0809
245.0776
247.0577
253.0506
256.0708
m/z
240 242 244 246 248 250 252 254 256 258 260 262 264 266 268 270
m/z 345, tR = 5.40 min
1: TOF MS ES+
5.99e6
313.0702
345.0963
314.0736
315.0761
327.0851
346.0994
257.0787
273.0382
285.0749
299.0902
250 260 270 280 290 300 310 320 330 340 350
m/z 345, tR = 5.84 min
1: TOF MS ES+
1.40e4
257.0794
259.0599
247.0609
243.0647
269.0802
270.0538
269.0441
241.0846
248.0631
254.0559
260.0613
240 245 250 255 260 265 270
m/z 345, tR = 5.84 min
1: TOF MS ES+
8.76e6
313.0721
345.0971
314.0738
346.0999
347.1022
257.0794
269.0802
285.0751
289.0696
312.0593
315.0763
327.0855
250 260 270 280 290 300 310 320 330 340 350

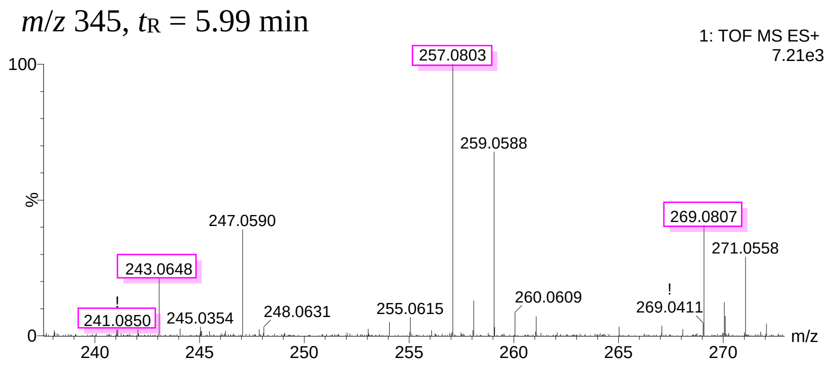
m/z 345, tR = 5.99 min
1: TOF MS ES+
7.21e3
100
%
0
257.0803
259.0588
247.0590
269.0807
271.0558
243.0648
260.0609
269.0411
241.0850
245.0354
248.0631
255.0615
240
245
250
255
260
265
270
m/z

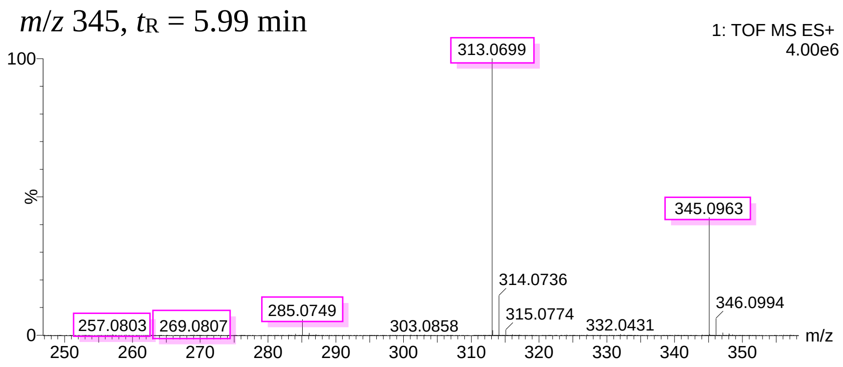
m/z 345, tR = 5.99 min
1: TOF MS ES+
4.00e6
100
%
0
313.0699
345.0963
314.0736
346.0994
315.0774
332.0431
285.0749
303.0858
257.0803
269.0807
250
260
270
280
290
300
310
320
330
340
350
m/z

Figure S3 Analysis of degraded products structures based on HR-ESI and MS/MS data at *m*/*z* 361, t_R = 4.47 min and 4.82 min.

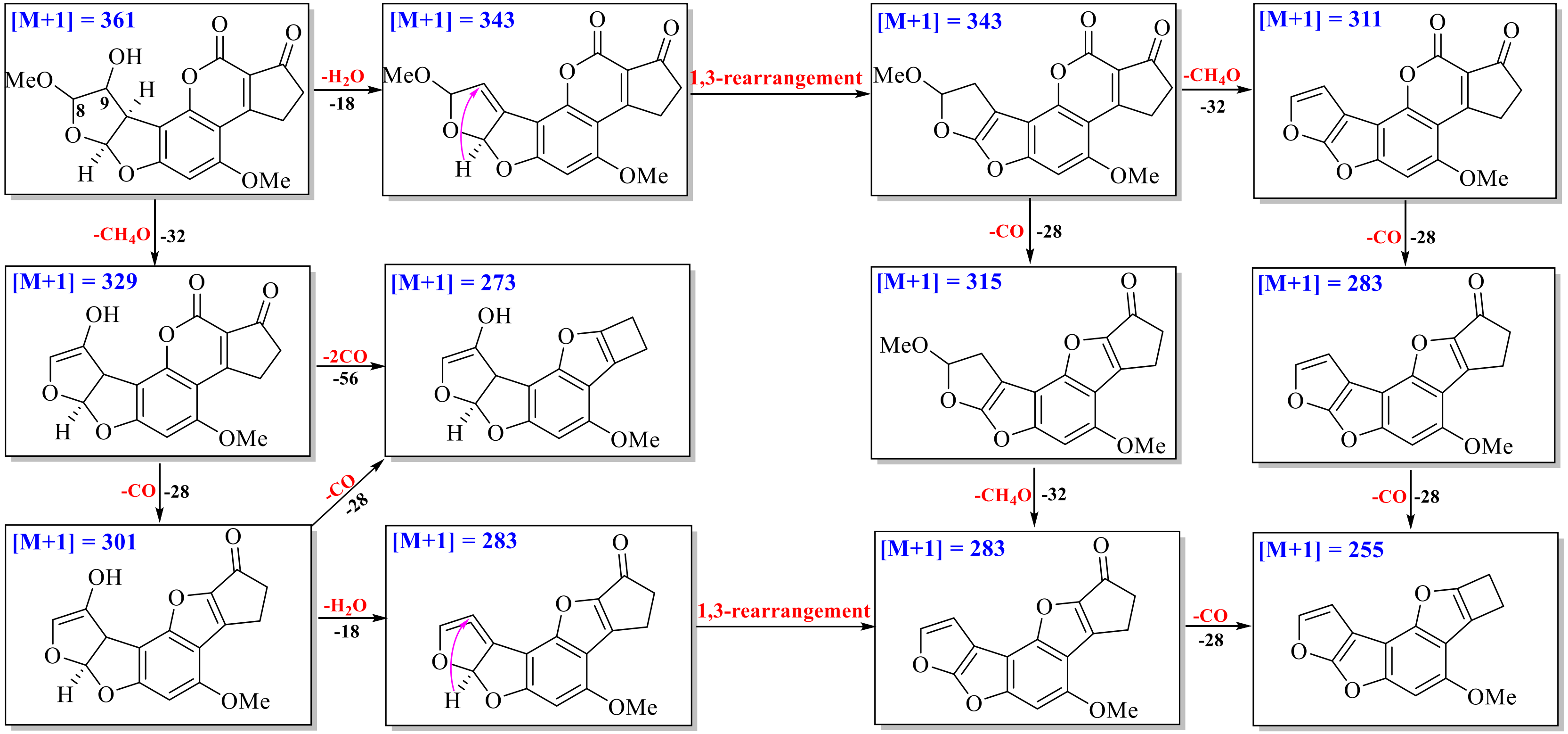

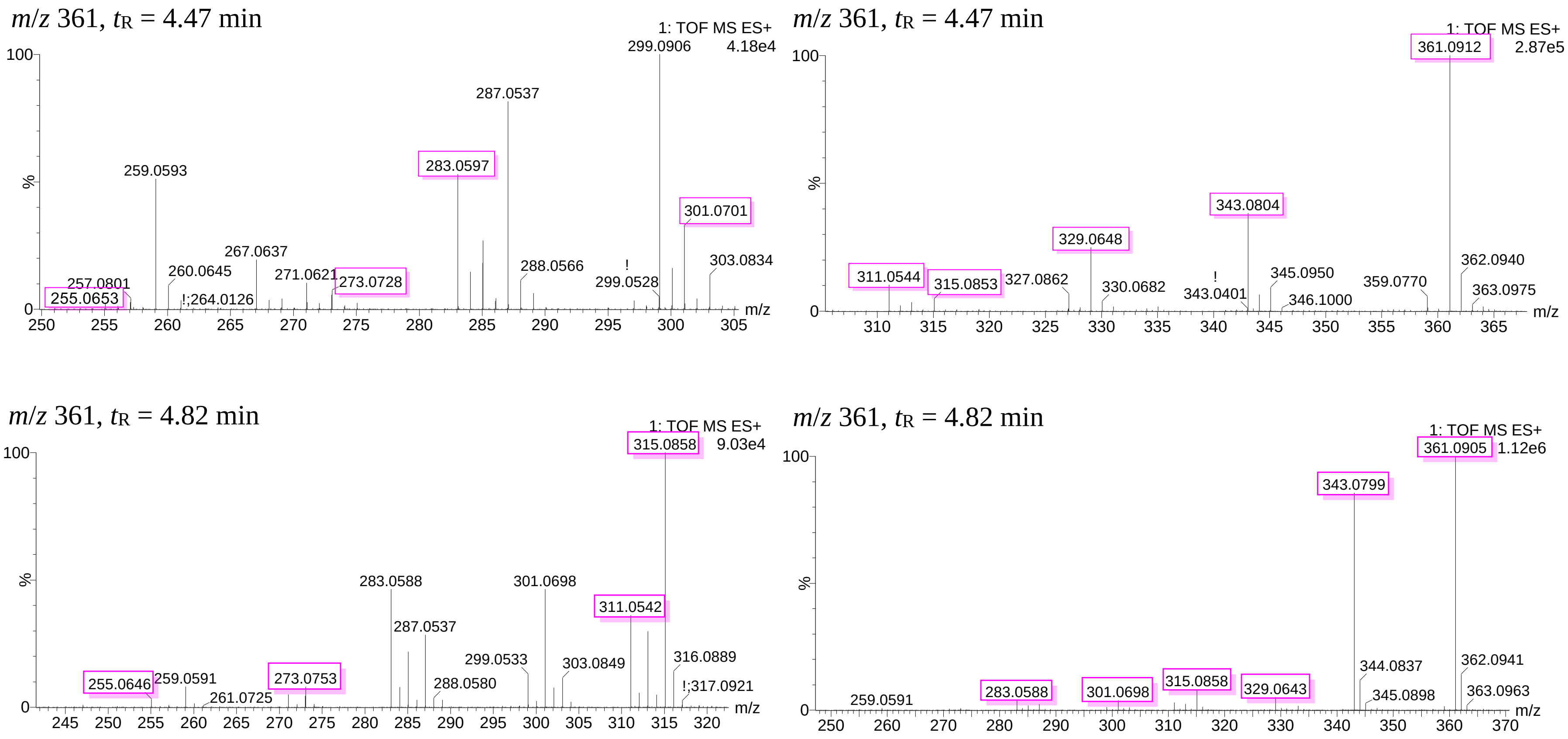
m/z 361, tR = 4.47 min
1: TOF MS ES+
4.18e4
299.0906
287.0537
283.0597
259.0593
301.0701
267.0637
260.0645
271.0621
273.0728
288.0566
299.0528
303.0834
257.0801
255.0653
264.0126
m/z 361, tR = 4.47 min
1: TOF MS ES+
361.0912
2.87e5
343.0804
329.0648
311.0544
315.0853
327.0862
330.0682
343.0401
345.0950
346.1000
359.0770
362.0940
363.0975
m/z 361, tR = 4.82 min
1: TOF MS ES+
315.0858
9.03e4
283.0588
301.0698
311.0542
287.0537
299.0533
303.0849
316.0889
255.0646
259.0591
261.0725
273.0753
288.0580
317.0921
m/z 361, tR = 4.82 min
1: TOF MS ES+
361.0905
1.12e6
343.0799
344.0837
362.0941
345.0898
363.0963
259.0591
283.0588
301.0698
315.0858
329.0643
%
m/z

Figure S4 Analysis of degraded products structures based on HR-ESI and MS/MS data at *m/z* 359, t_R = 4.94 min, 7.08 min and 7.30 min.

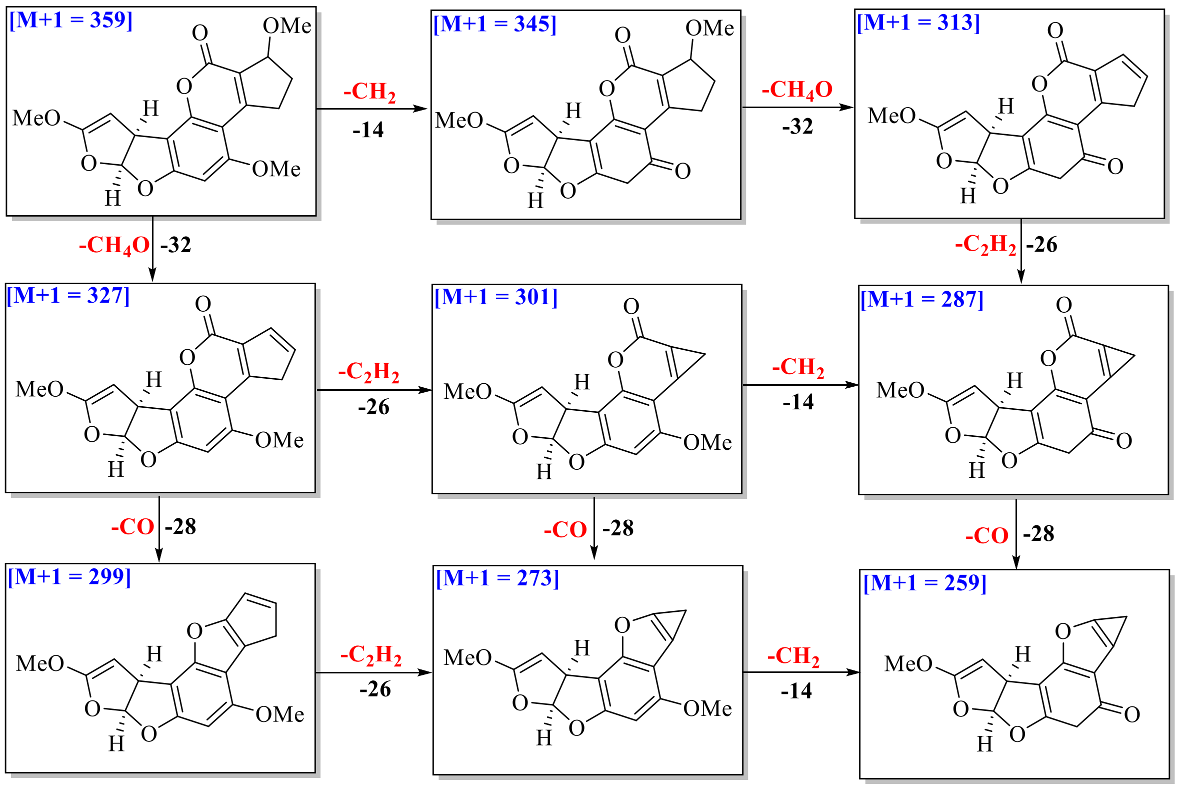

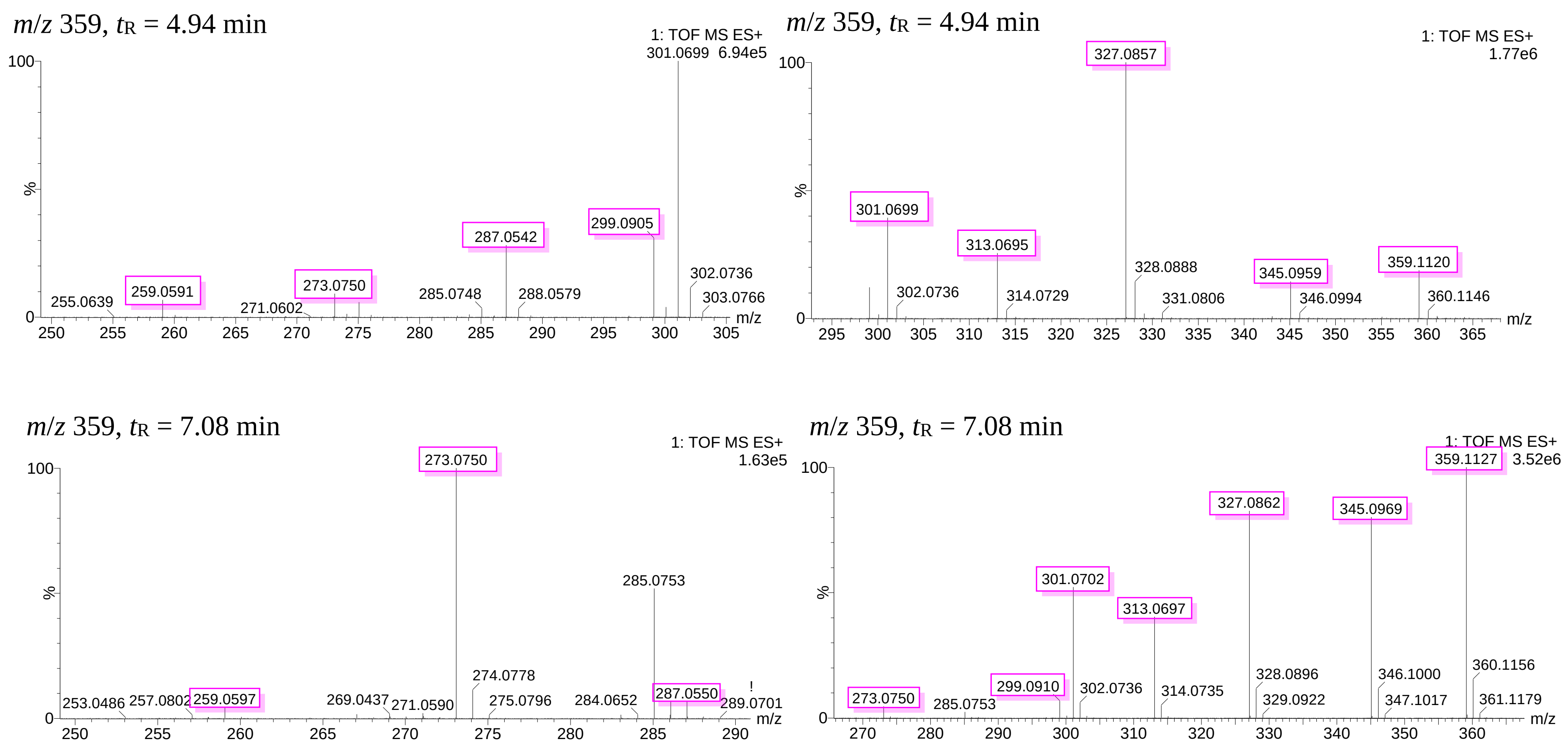
m/z 359, tR = 4.94 min
1: TOF MS ES+
301.0699 6.94e5
100
%
0
255.0639
259.0591
271.0602
273.0750
285.0748
287.0542
288.0579
299.0905
302.0736
303.0766
250 255 260 265 270 275 280 285 290 295 300 305
m/z
m/z 359, tR = 4.94 min
1: TOF MS ES+
1.77e6
327.0857
301.0699
302.0736
313.0695
314.0729
328.0888
331.0806
345.0959
346.0994
359.1120
360.1146
295 300 305 310 315 320 325 330 335 340 345 350 355 360 365
m/z
m/z 359, tR = 7.08 min
1: TOF MS ES+
1.63e6
273.0750
285.0753
274.0778
253.0486
257.0802
259.0597
269.0437
271.0590
275.0796
284.0652
287.0550
289.0701
250 255 260 265 270 275 280 285 290
m/z
m/z 359, tR = 7.08 min
1: TOF MS ES+
3.52e6
359.1127
327.0862
345.0969
301.0702
313.0697
273.0750
285.0753
299.0910
302.0736
314.0735
328.0896
329.0922
346.1000
347.1017
360.1156
361.1179
270 280 290 300 310 320 330 340 350 360
m/z

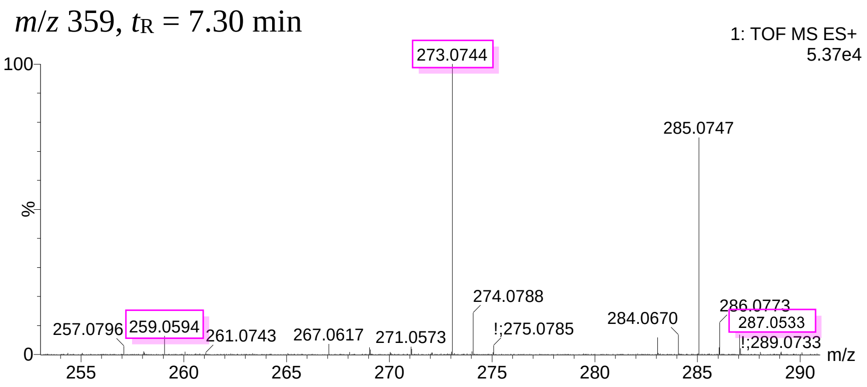
m/z 359, tR = 7.30 min
1: TOF MS ES+
5.37e4
100
%
0
273.0744
285.0747
274.0788
257.0796
259.0594
261.0743
267.0617
271.0573
275.0785
284.0670
286.0773
287.0533
289.0733
m/z
255
260
265
270
275
280
285
290

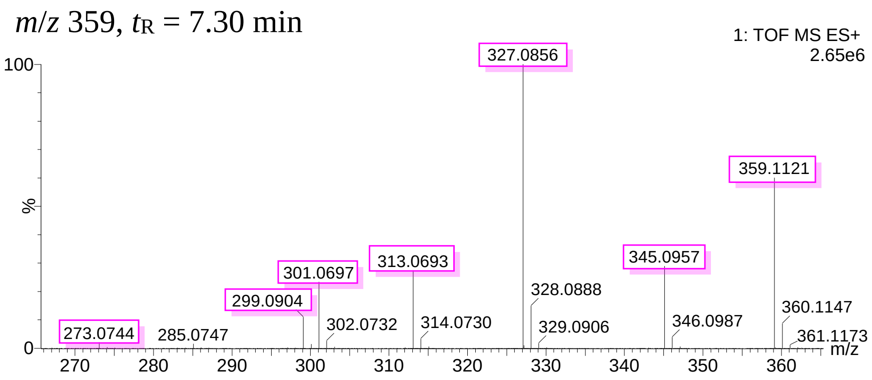
m/z 359, tR = 7.30 min
1: TOF MS ES+
2.65e6
100
%
0
327.0856
359.1121
345.0957
313.0693
301.0697
299.0904
328.0888
360.1147
273.0744
285.0747
302.0732
314.0730
329.0906
346.0987
361.1173
m/z
270
280
290
300
310
320
330
340
350
360

Figure S5 Analysis of degraded products structures based on HR-ESI and MS/MS data at *m/z* 391, t_R = 6.41 min.

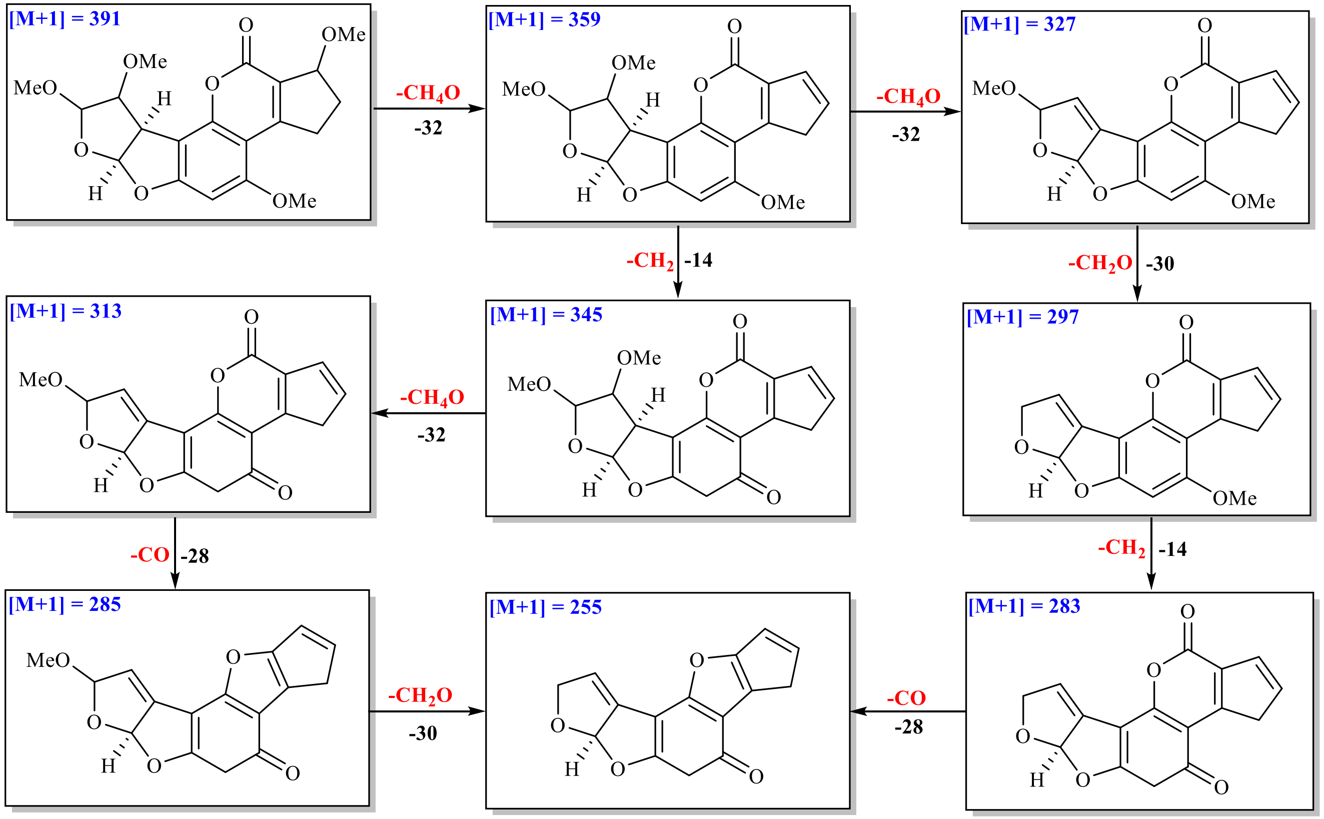

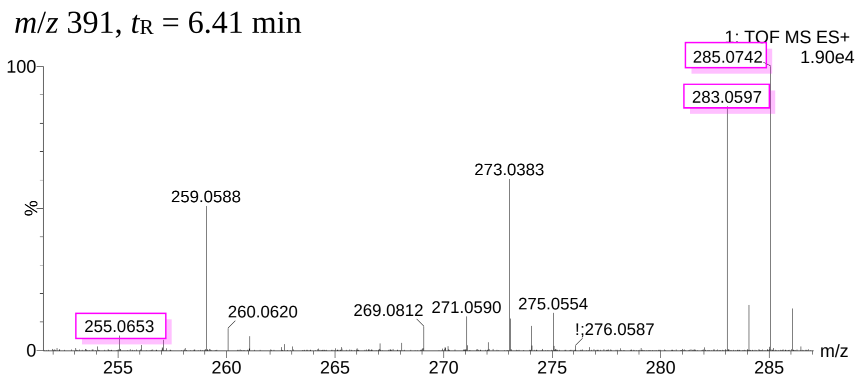
m/z 391, tR = 6.41 min
1: TOF MS ES+
1.90e4
285.0742
283.0597
273.0383
259.0588
260.0620
269.0812
271.0590
275.0554
276.0587
255.0653
100
%
0
m/z
255
260
265
270
275
280
285

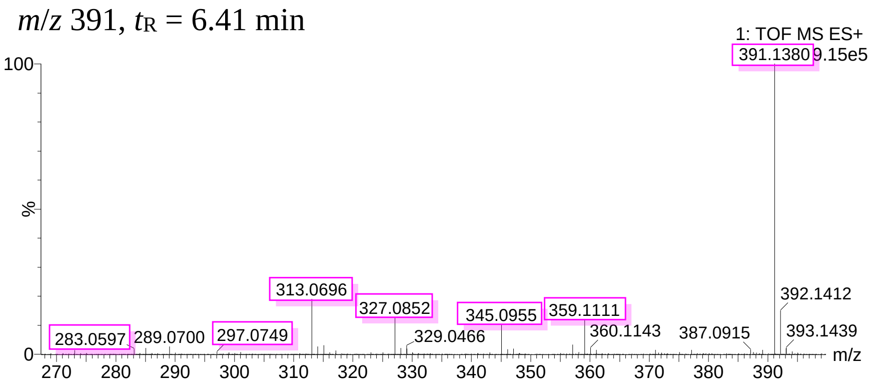
m/z 391, tR = 6.41 min
1: TOF MS ES+
391.1380
9.15e5
392.1412
393.1439
313.0696
327.0852
345.0955
359.1111
360.1143
387.0915
283.0597
289.0700
297.0749
329.0466
100
%
0
m/z
270
280
290
300
310
320
330
340
350
360
370
380
390

Figure S6 Analysis of degraded products structures based on HR-ESI and MS/MS data at *m*/*z* 331, t_R = 4.06 min and 4.18 min.

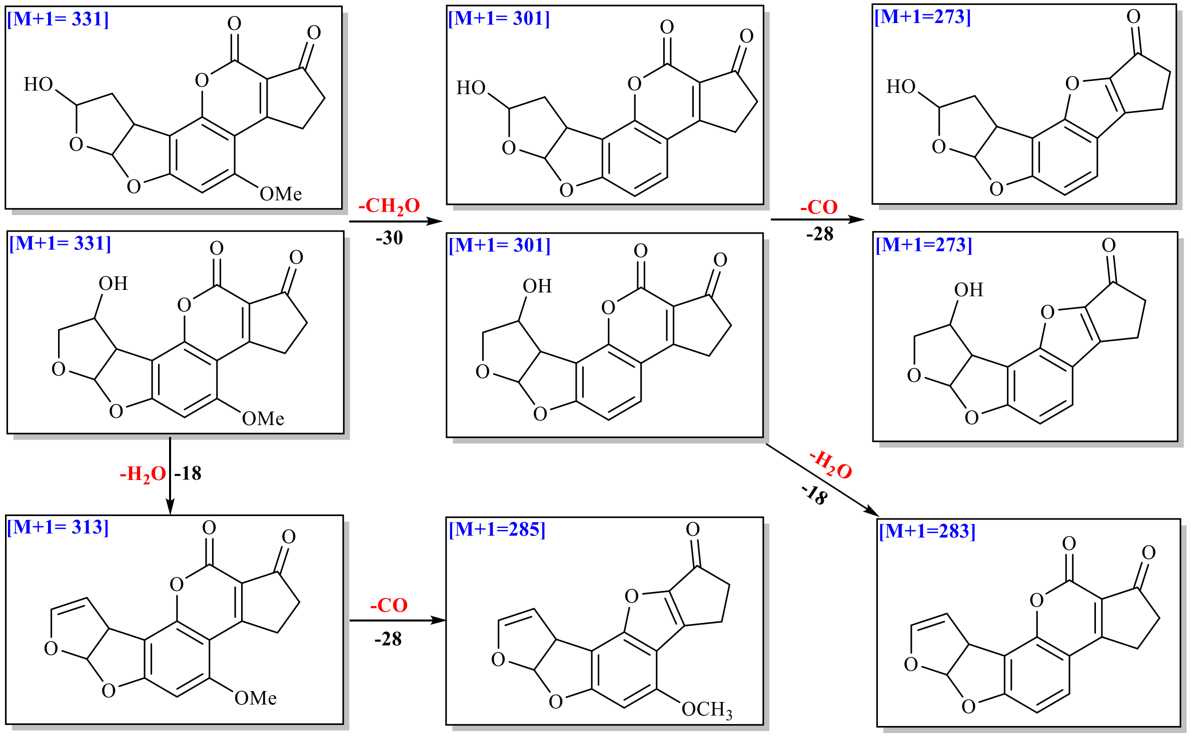

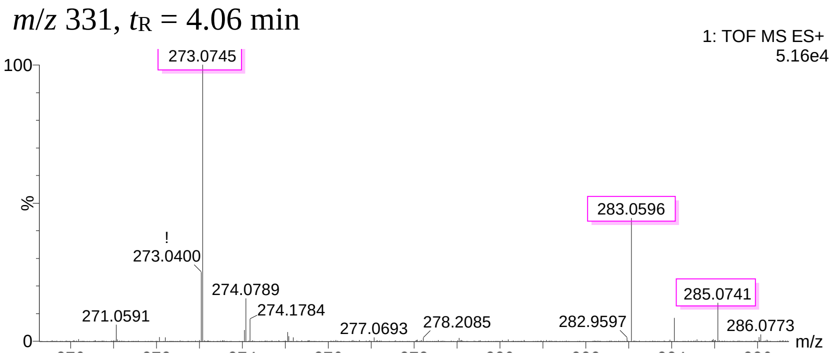
m/z 331, tR = 4.06 min
1: TOF MS ES+
5.16e4
100
%
0
273.0745
283.0596
285.0741
273.0400
274.0789
274.1784
271.0591
277.0693
278.2085
282.9597
286.0773
m/z

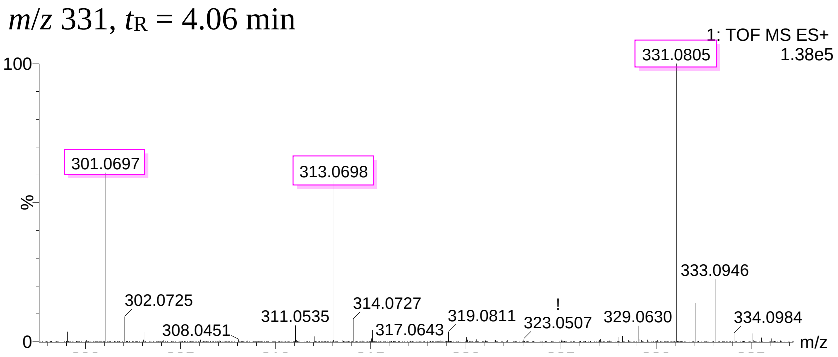
m/z 331, tR = 4.06 min
1: TOF MS ES+
1.38e5
100
%
0
331.0805
301.0697
313.0698
333.0946
302.0725
314.0727
311.0535
308.0451
317.0643
319.0811
323.0507
329.0630
334.0984
m/z

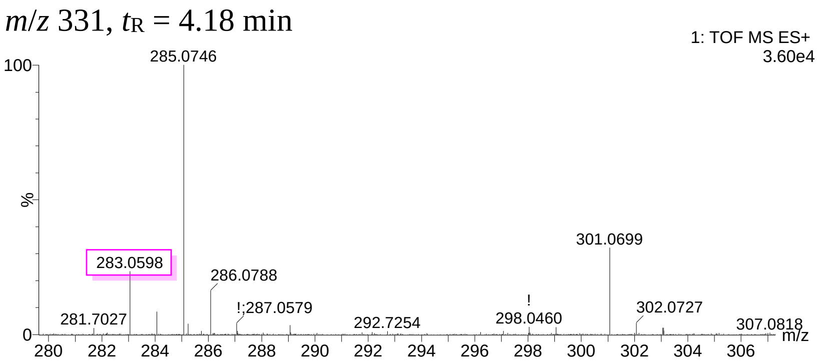
m/z 331, tR = 4.18 min
1: TOF MS ES+
3.60e4
100
%
0
285.0746
301.0699
283.0598
286.0788
287.0579
281.7027
292.7254
298.0460
302.0727
307.0818
m/z
280
282
284
286
288
290
292
294
296
298
300
302
304
306

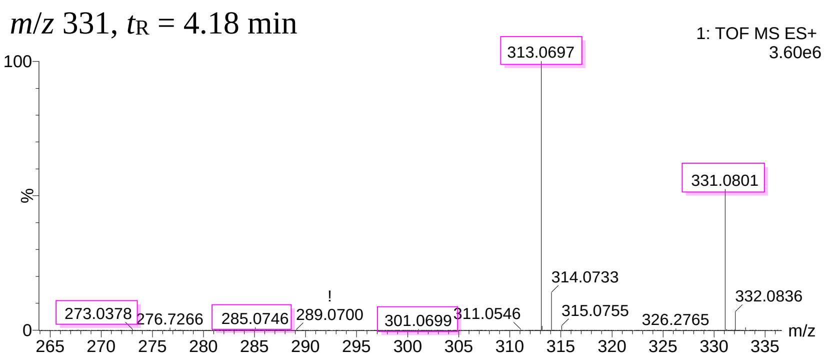
m/z 331, tR = 4.18 min
1: TOF MS ES+
3.60e6
100
%
0
313.0697
331.0801
314.0733
332.0836
273.0378
276.7266
285.0746
289.0700
301.0699
311.0546
315.0755
326.2765
m/z
265
270
275
280
285
290
295
300
305
310
315
320
325
330
335

Figure S7 Analysis of degraded products structures based on HR-ESI and MS/MS data at *m*/*z* 347, t_R = 3.48 min.

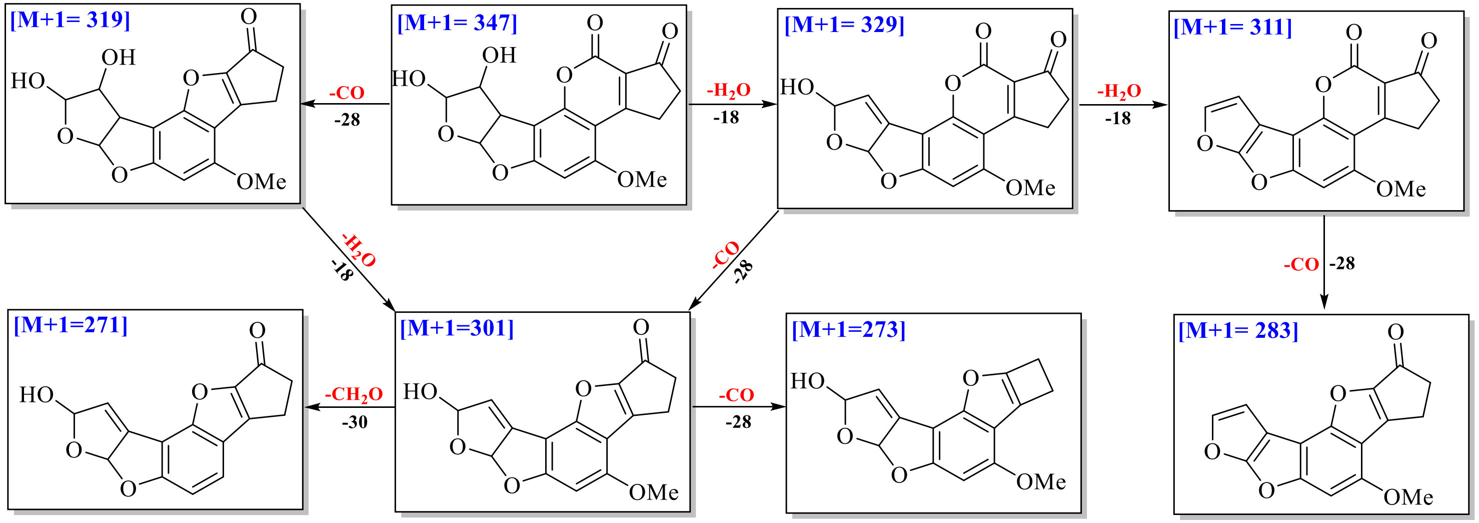

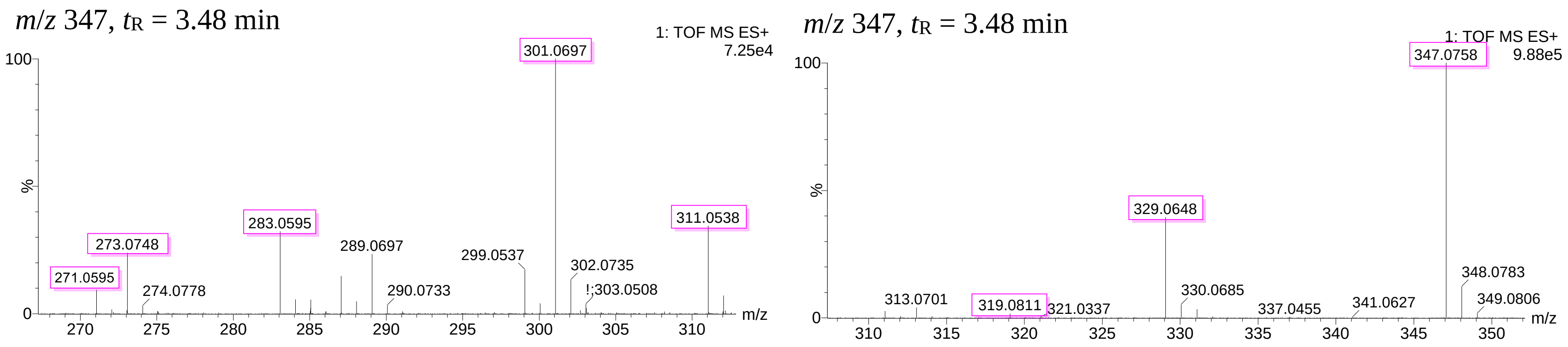

Figure S8 Analysis of degraded products structures based on HR-ESI and MS/MS data at *m/z* 371, t_R = 4.34 min and 5.63 min.

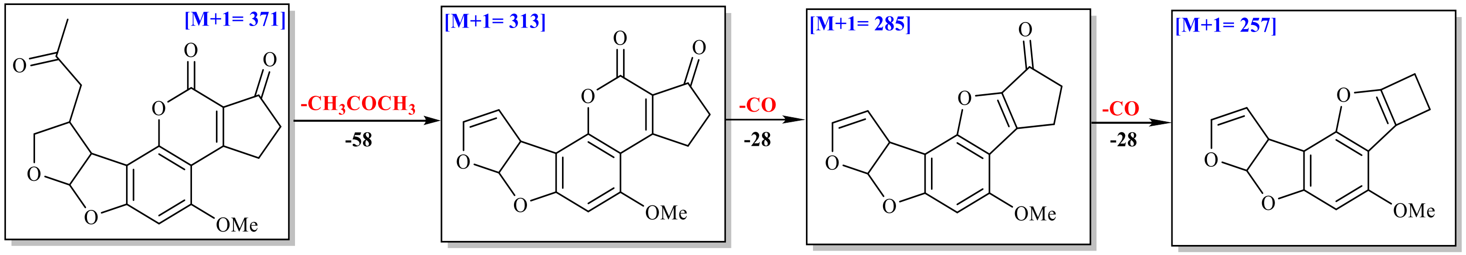

m/z 371, t_R = 4.34 min

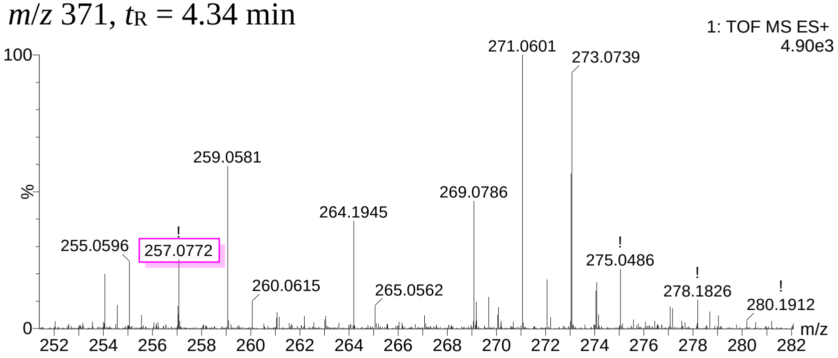

m/z 371, t_R = 4.34 min

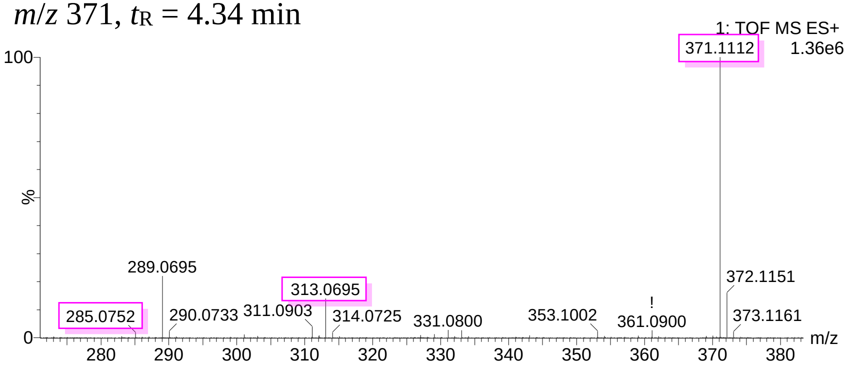

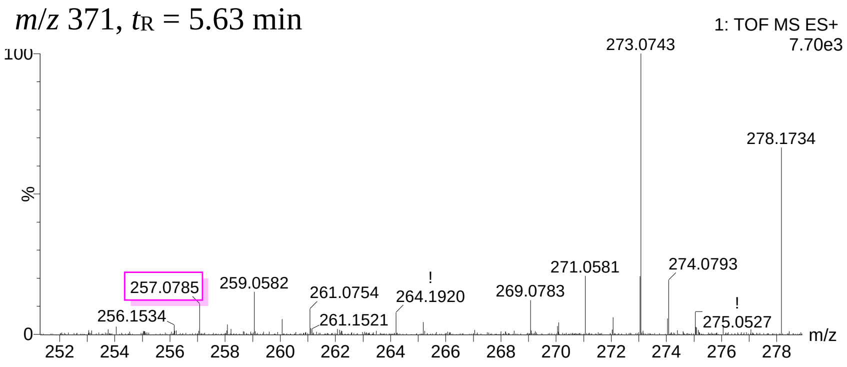
m/z 371, tR = 5.63 min
1: TOF MS ES+
7.70e3
273.0743
278.1734
%
100
0
257.0785
259.0582
261.0754
264.1920
269.0783
271.0581
274.0793
256.1534
261.1521
275.0527
252 254 256 258 260 262 264 266 268 270 272 274 276 278
m/z

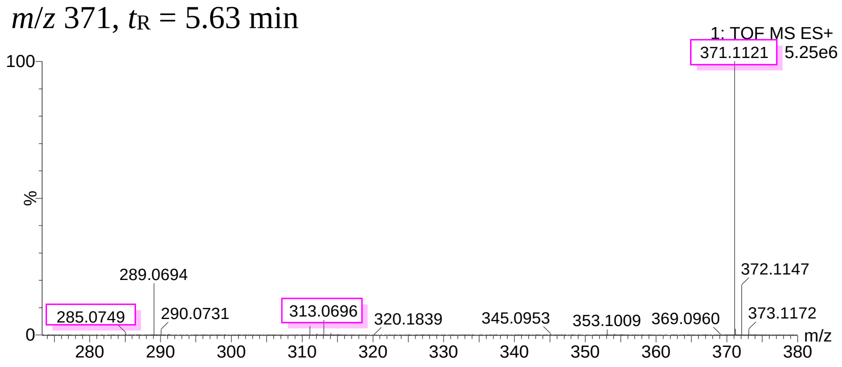
m/z 371, tR = 5.63 min
1: TOF MS ES+
371.1121
5.25e6
100
%
0
289.0694
372.1147
285.0749
290.0731
313.0696
320.1839
345.0953
353.1009
369.0960
373.1172
280 290 300 310 320 330 340 350 360 370 380
m/z

Figure S9 Analysis of degraded products structures based on HR-ESI and MS/MS data at *m*/*z* 401, t_R = 4.86 min and 6.54 min.

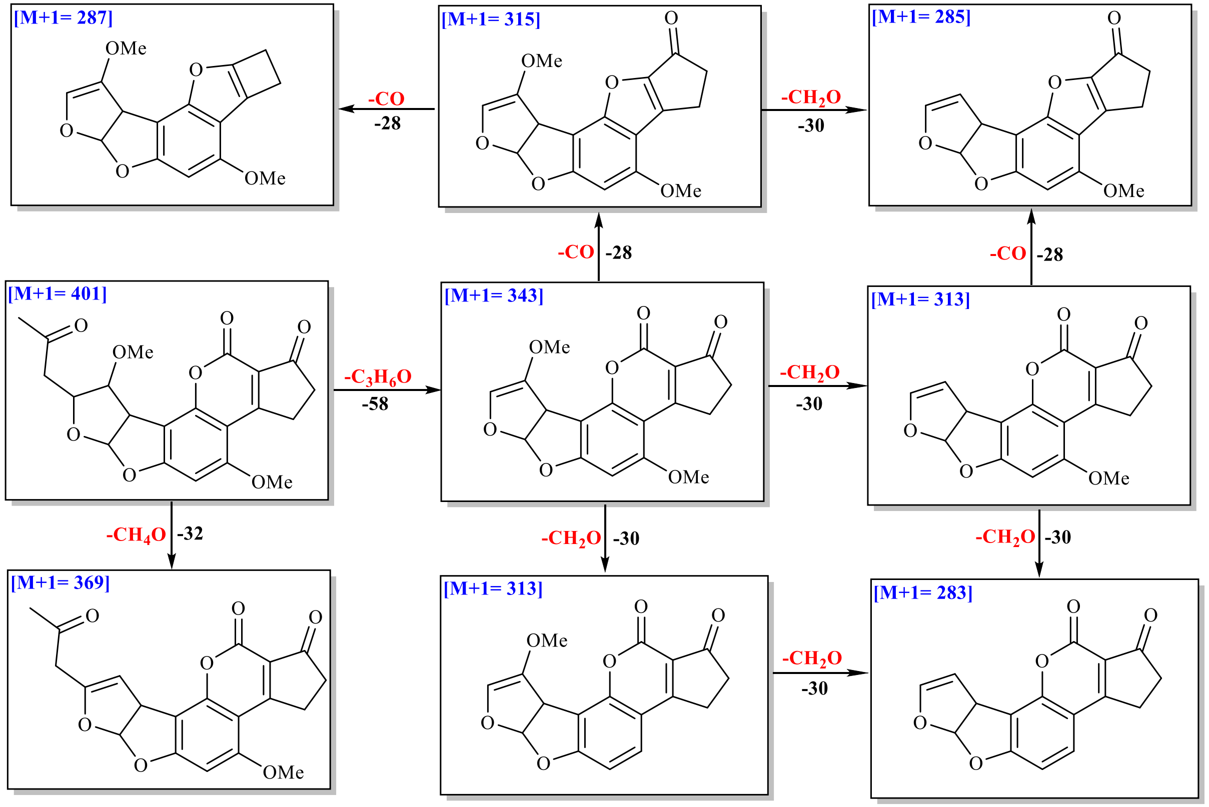

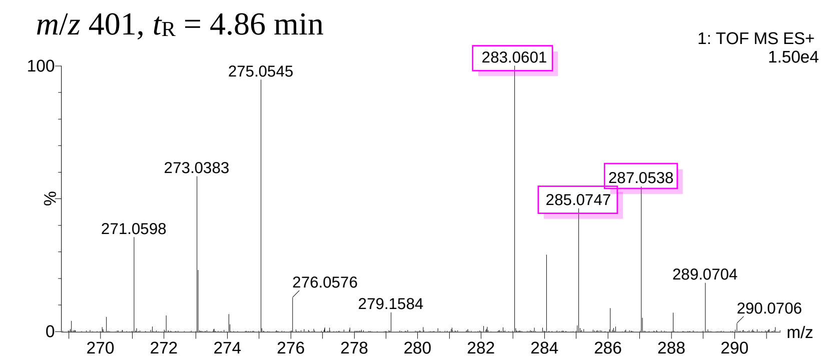
m/z 401, tR = 4.86 min
1: TOF MS ES+
1.50e4
100
%
0
283.0601
275.0545
273.0383
271.0598
285.0747
287.0538
276.0576
279.1584
289.0704
290.0706
270
272
274
276
278
280
282
284
286
288
290
m/z

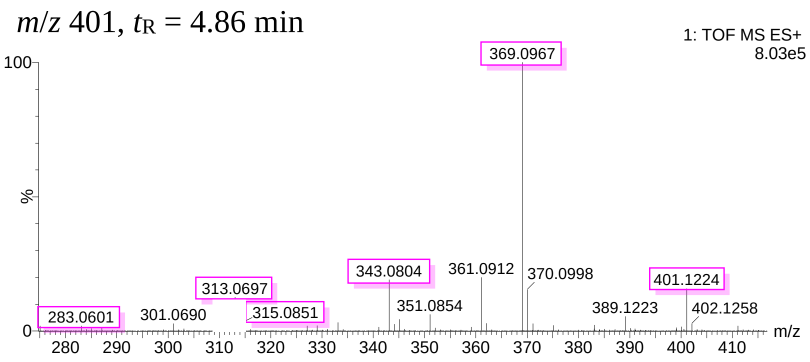
m/z 401, tR = 4.86 min
1: TOF MS ES+
8.03e5
100
%
0
369.0967
343.0804
361.0912
370.0998
401.1224
313.0697
315.0851
351.0854
389.1223
402.1258
283.0601
301.0690
280
290
300
310
320
330
340
350
360
370
380
390
400
410
m/z

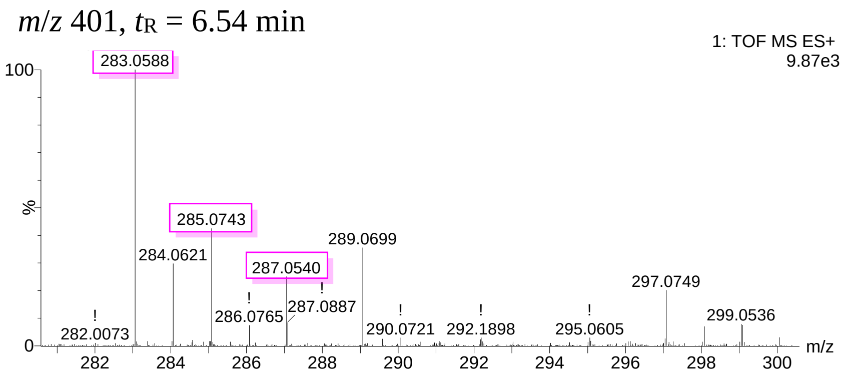
m/z 401, tR = 6.54 min
1: TOF MS ES+
9.87e3
100
%
0
283.0588
285.0743
284.0621
289.0699
287.0540
297.0749
287.0887
286.0765
299.0536
282.0073
290.0721
292.1898
295.0605
282
284
286
288
290
292
294
296
298
300
m/z

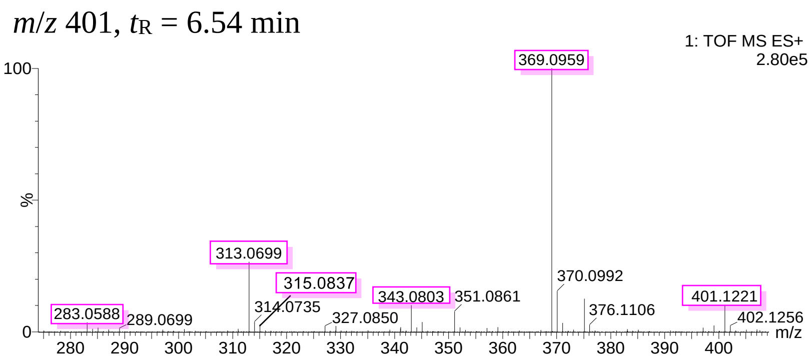
m/z 401, tR = 6.54 min
1: TOF MS ES+
2.80e5
100
%
0
369.0959
313.0699
370.0992
315.0837
314.0735
343.0803
351.0861
376.1106
401.1221
283.0588
289.0699
327.0850
402.1256
280
290
300
310
320
330
340
350
360
370
380
390
400
m/z

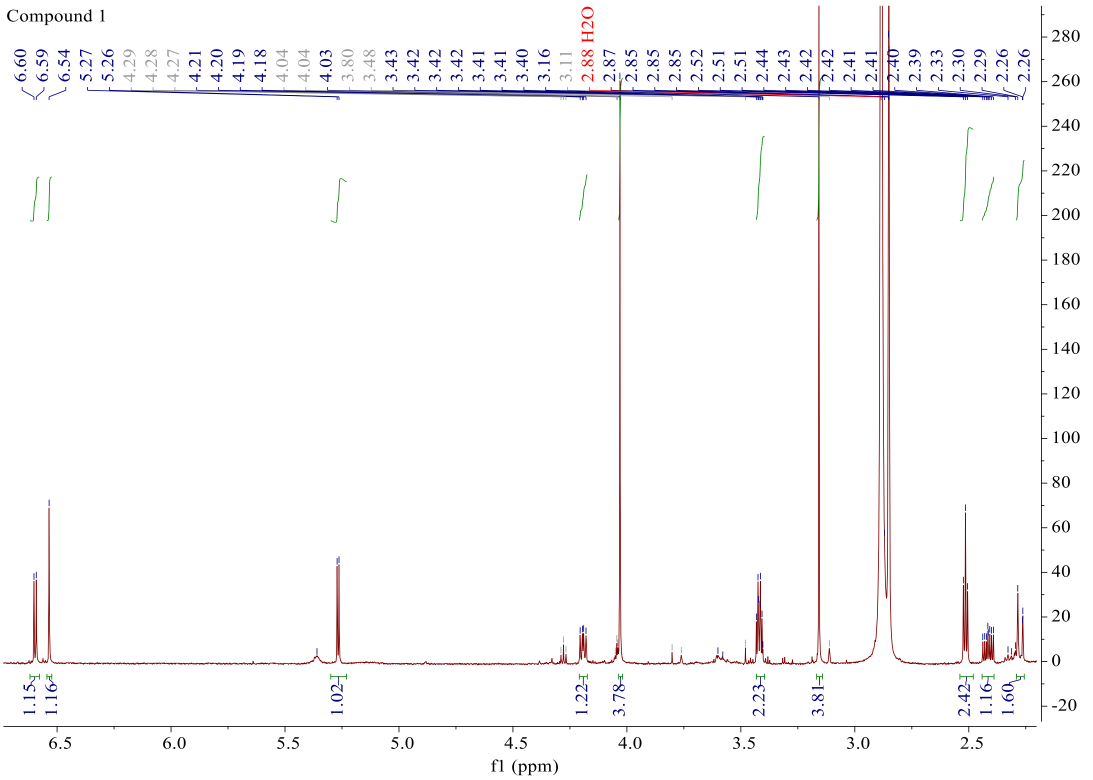

Figure S10 ^{1}H-NMR spectrum of compound **1** (Acetone-d_6, 600MHz)

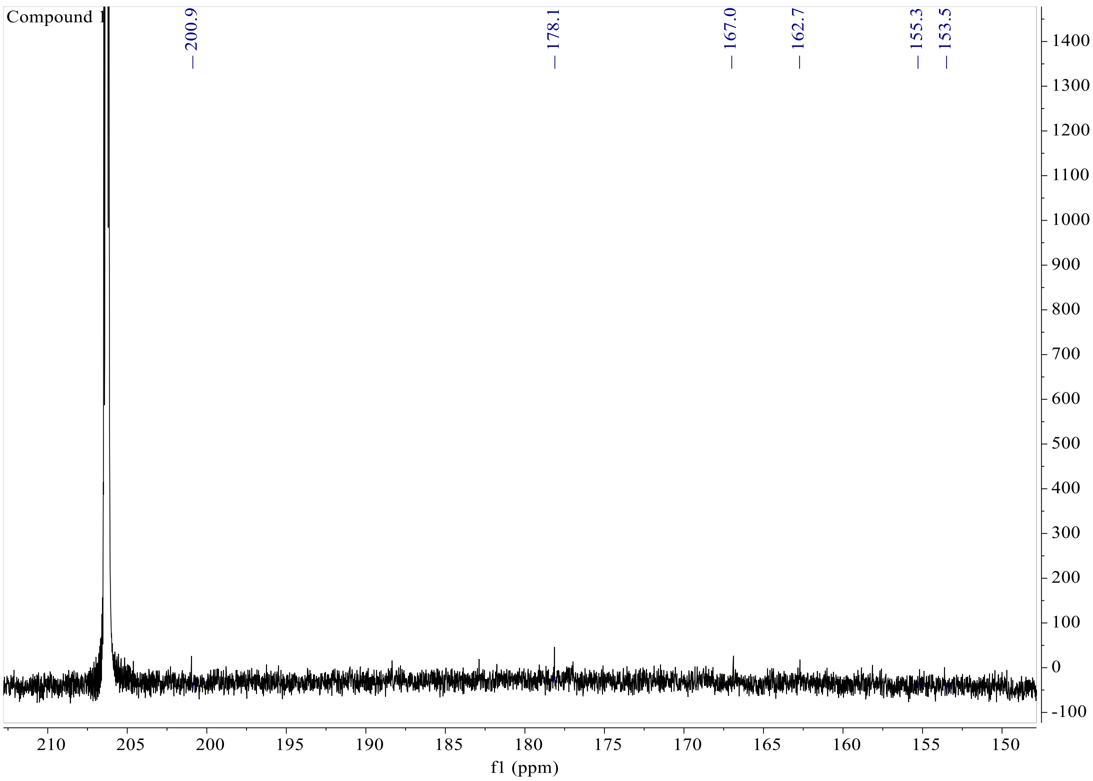

Figure S11-a ^{13}C-NMR spectrum of compound **1** (Acetone-d_6, 125 MHz)

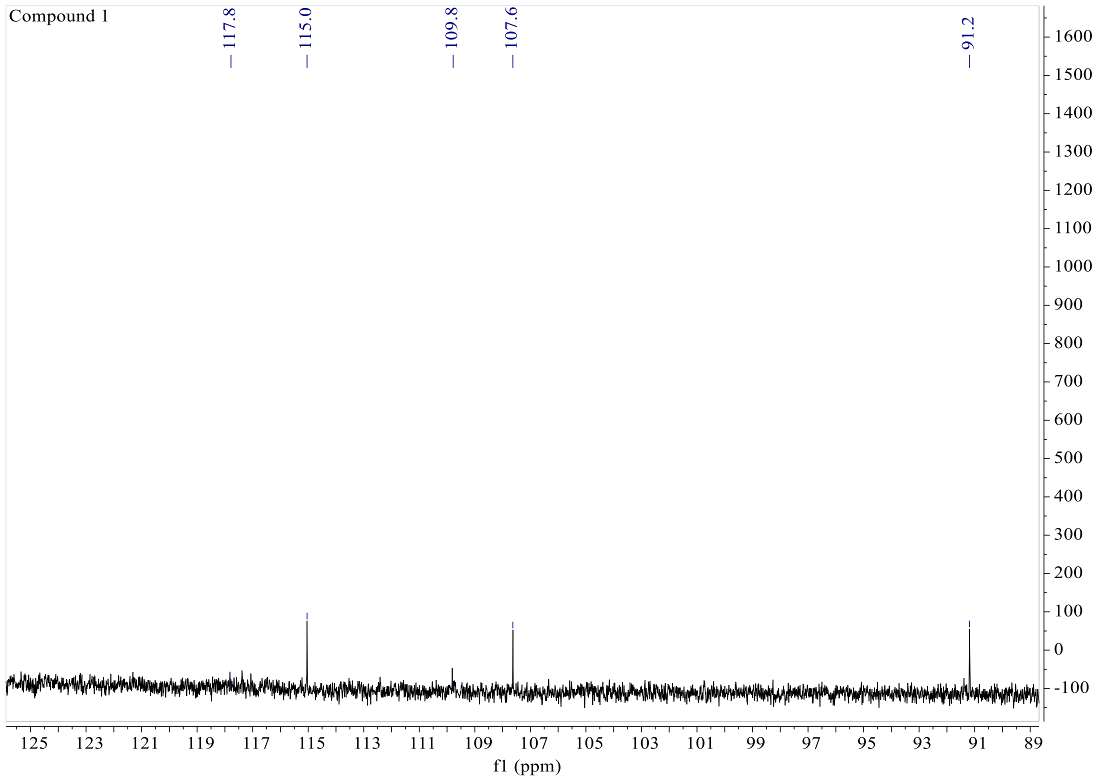

Figure S11-b ^{13}C-NMR spectrum of compound **1** (Acetone-d_6, 125 MHz)

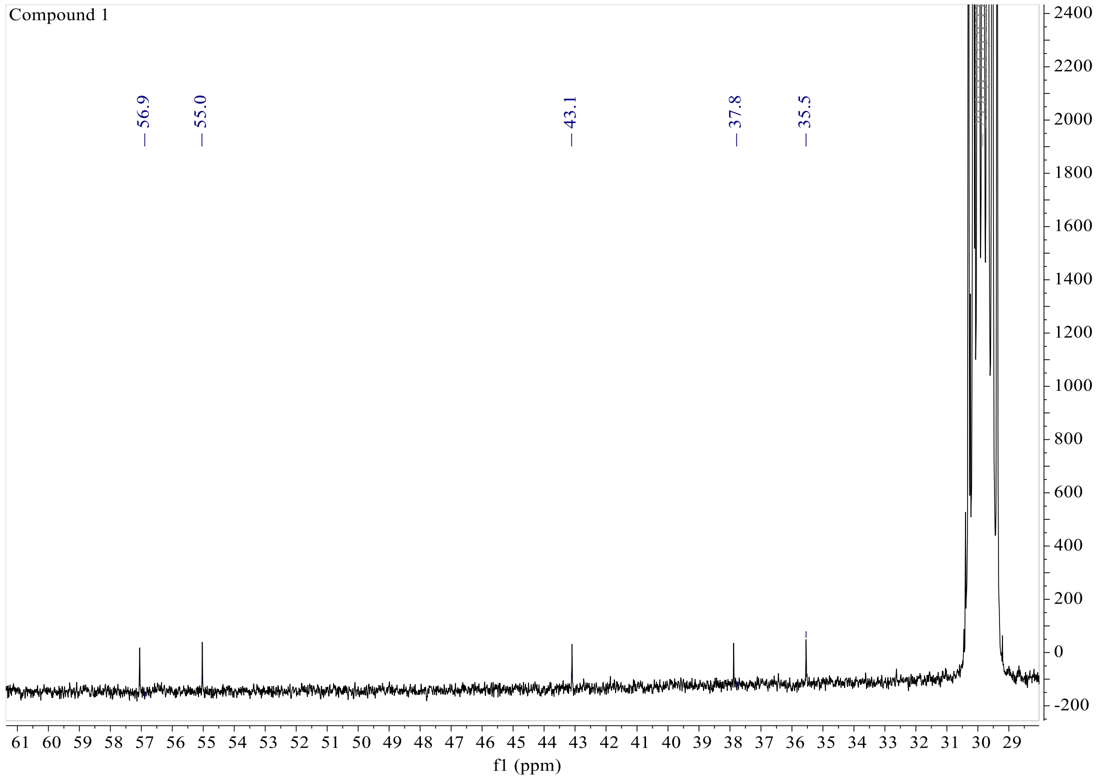

Figure S11-c ^{13}C-NMR spectrum of compound **1** (Acetone-d_6, 125 MHz)

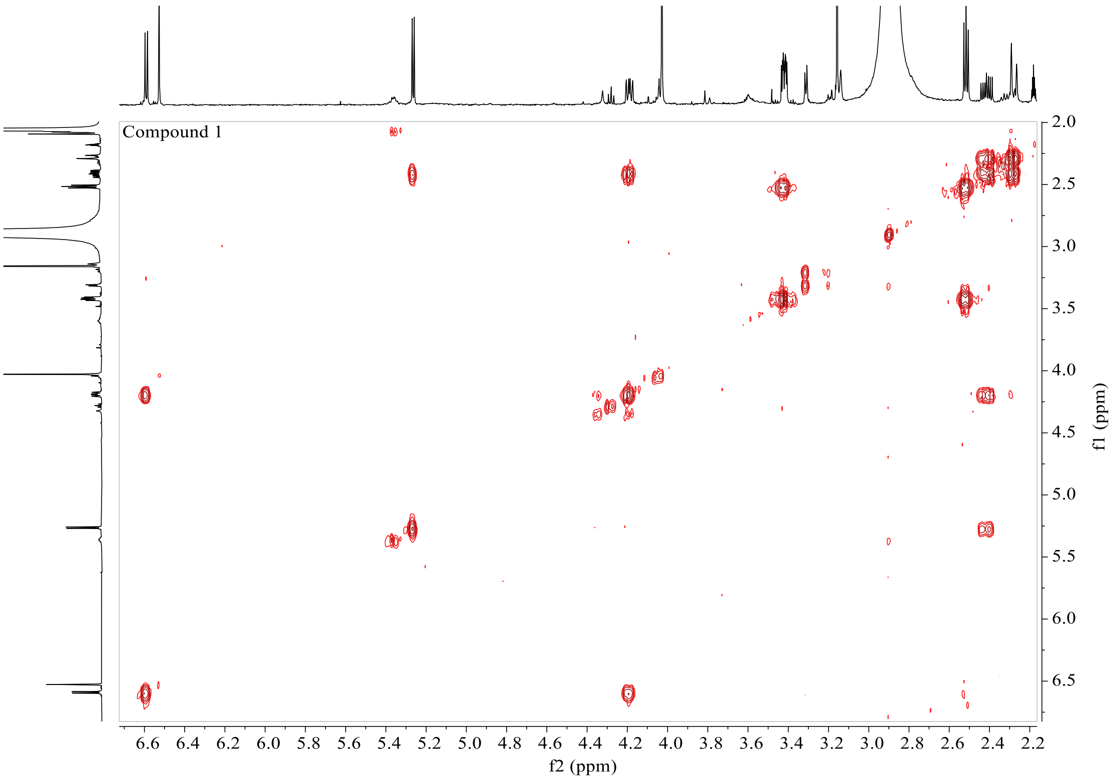

Figure S12 ^{1}H-^{1}H COSY spectrum of compound **1** (Acetone-d_6, 500 MHz)

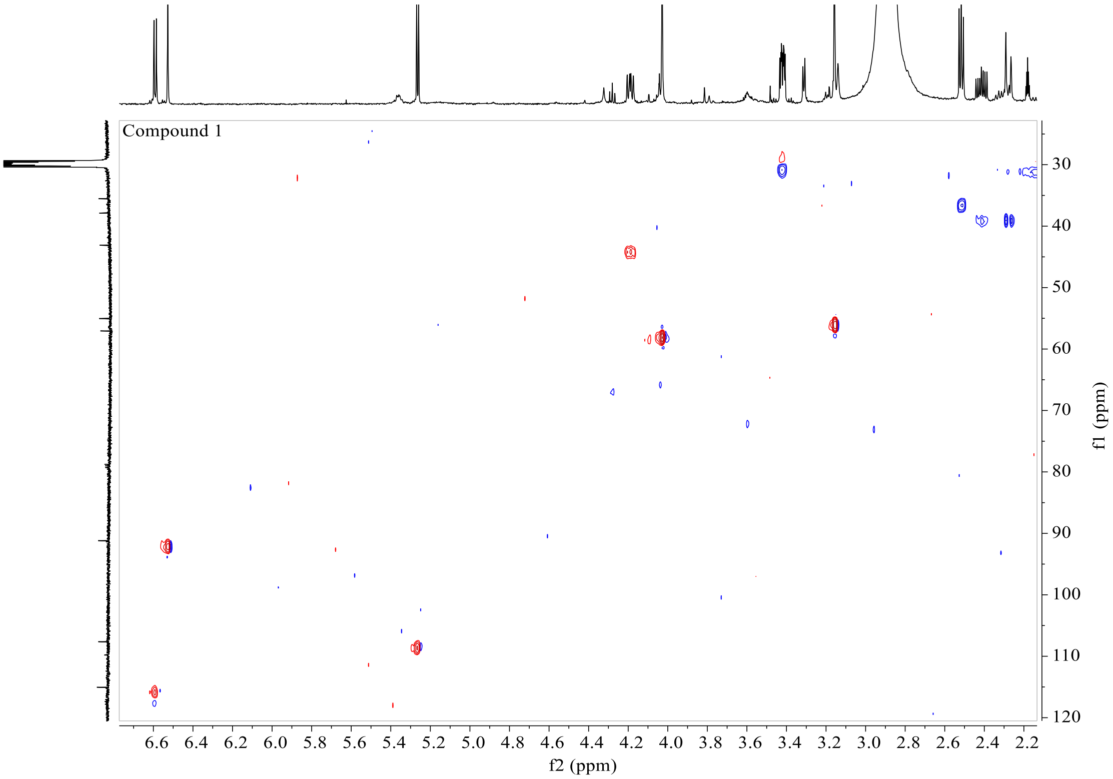

Figure S13 HSQC spectrum of compound **1** (Acetone-d_6, 500 MHz)

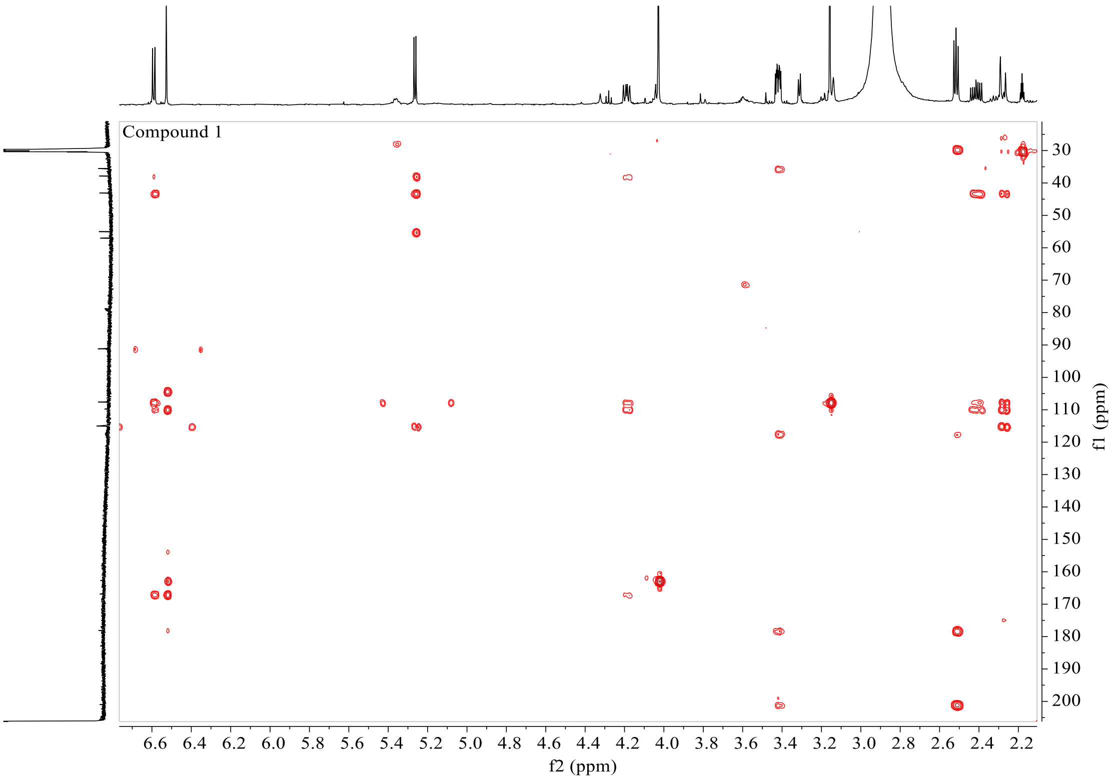

Figure S14 HMBC spectrum of compound **1** (Acetone-*d*6, 500 MHz)

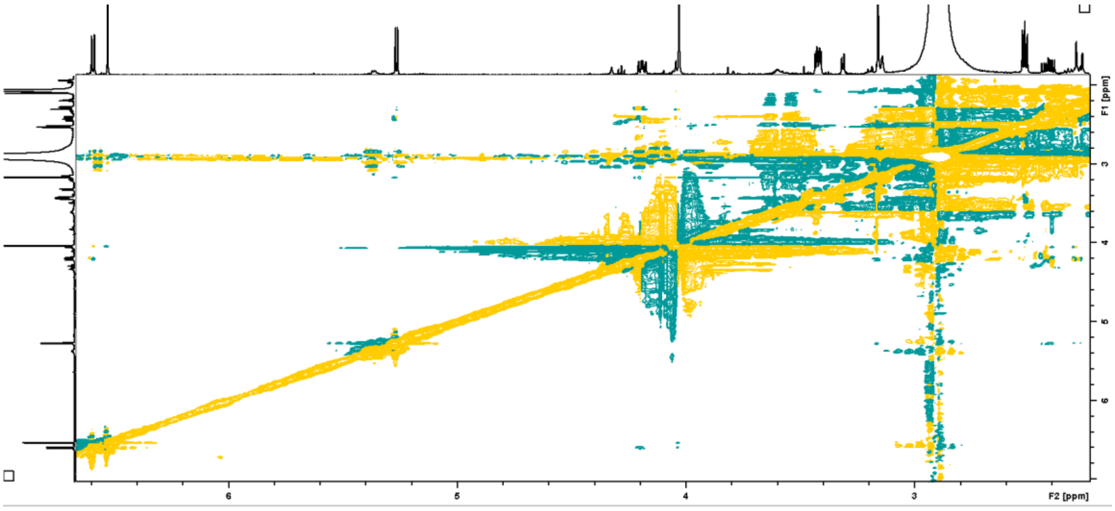

Figure S15 ROESY spectrum of compound **1** (Acetone-*d*6, 500 MHz)

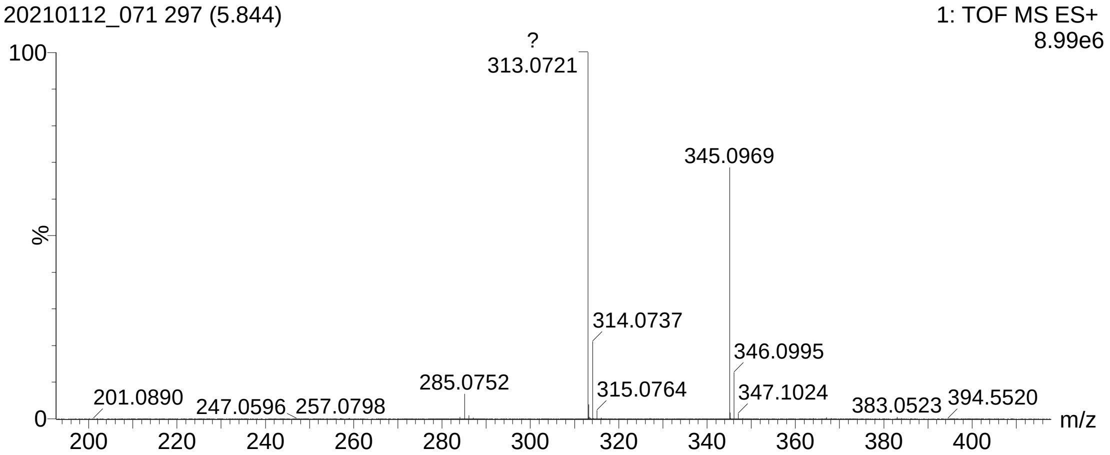

Figure S16 HR-ESI-MS spectrum of compound **1**

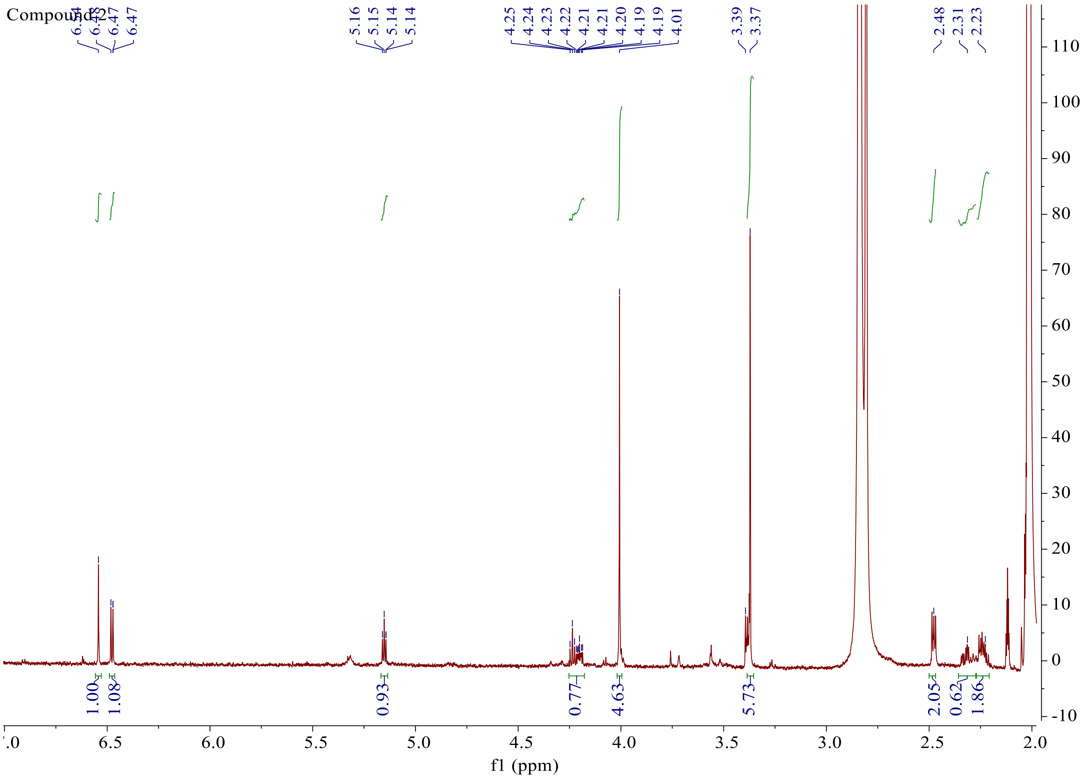

Figure S17 ^{1}H-NMR spectrum of compound **2** (Acetone-d_6, 600MHz)

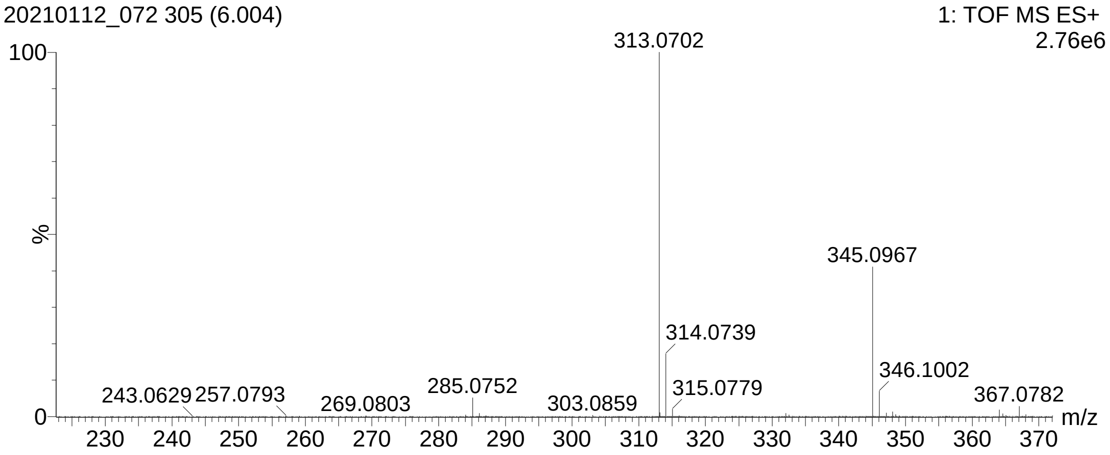

Figure S18 HR-ESI-MS spectrum of compound **2**

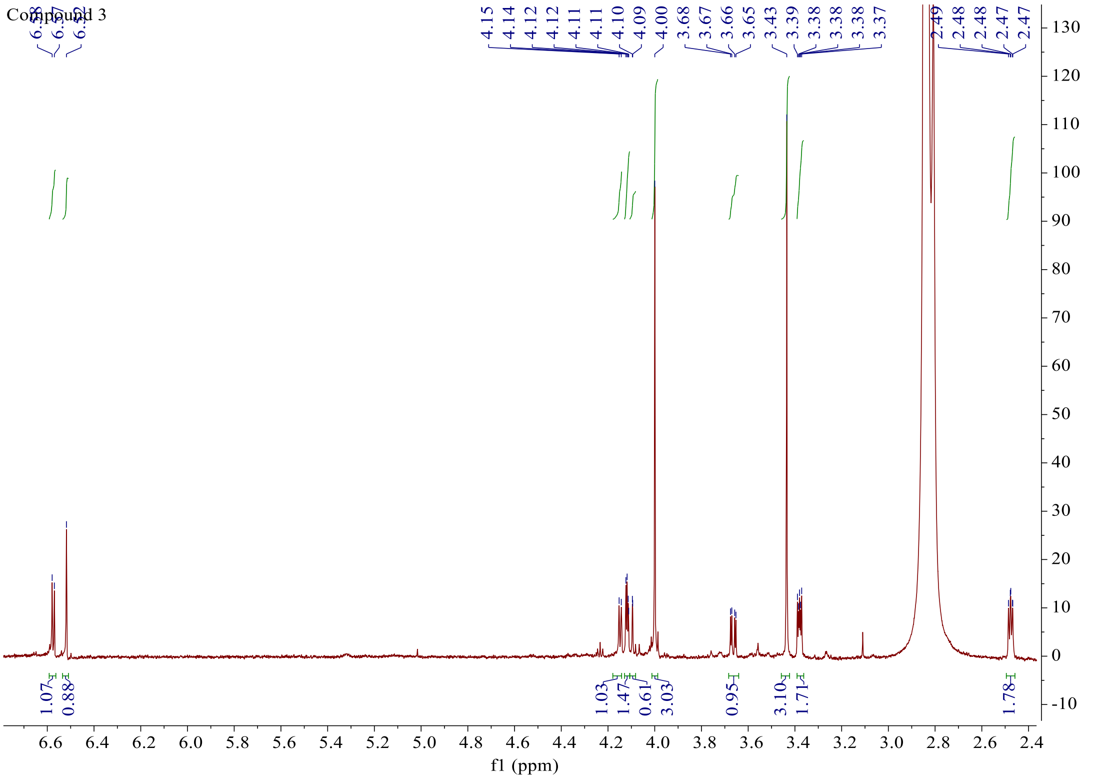

Figure S19 ^{1}H-NMR spectrum of compound **3** (Acetone-d_6, 600 MHz)

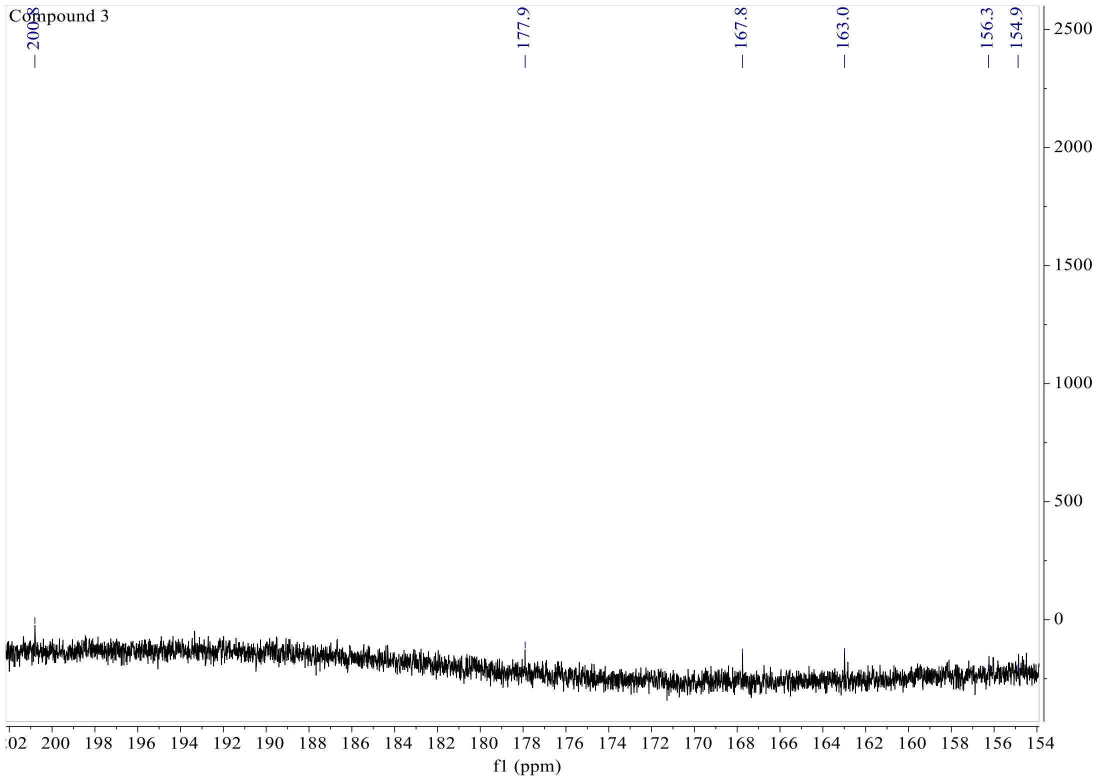

Figure S20-a ^{13}C-NMR spectrum of compound **3** (Acetone-*d*6, 125 MHz)

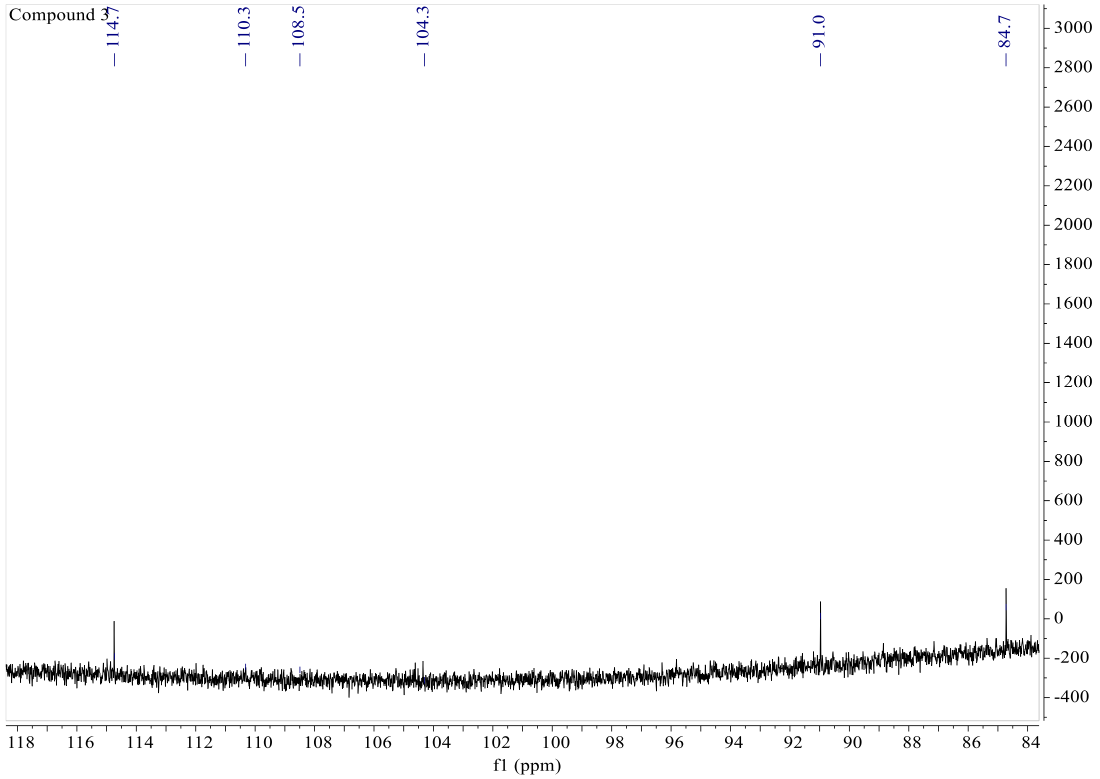

Figure S20-b ^{13}C-NMR spectrum of compound **3** (Acetone-*d*6, 125 MHz)

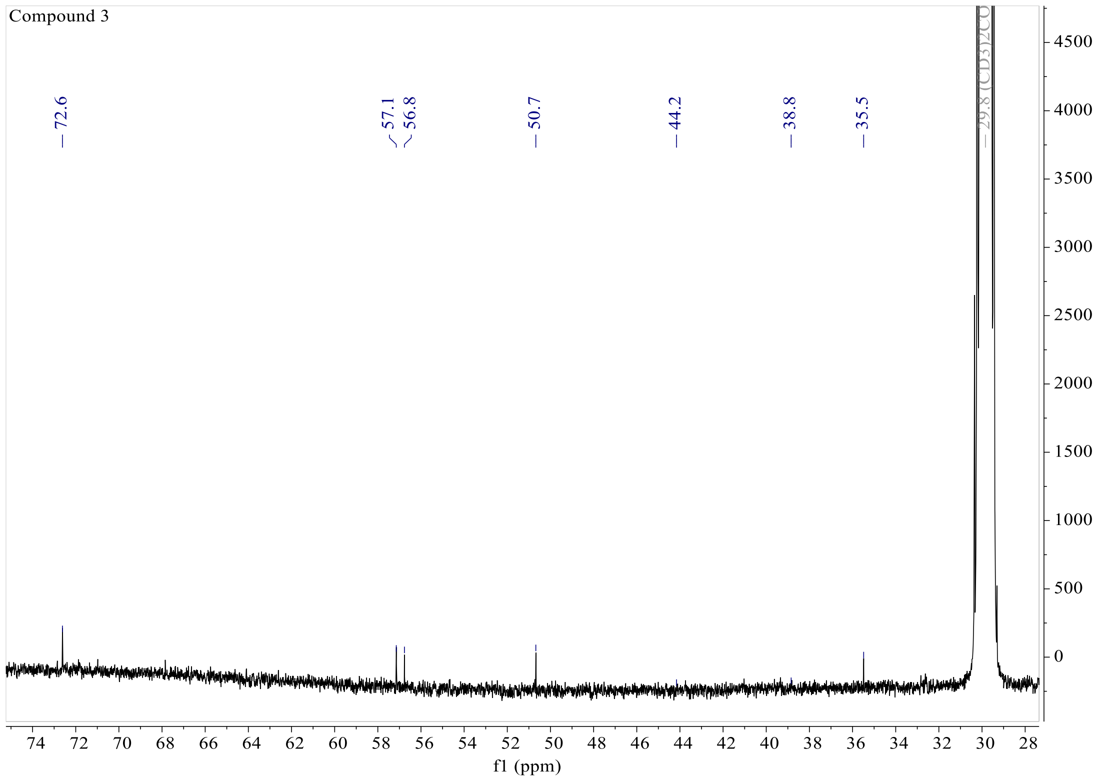

Figure S20-c ^{13}C-NMR spectrum of compound **3** (Acetone-d_6, 125 MHz)

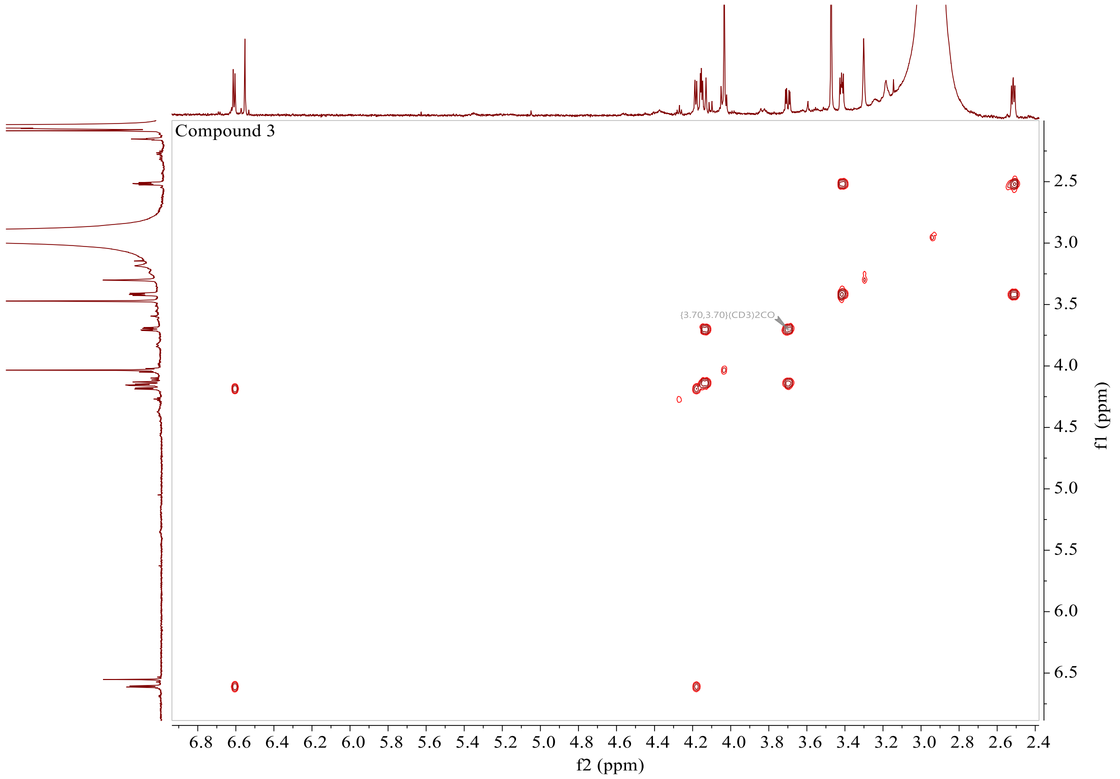

Figure S21 ^{1}H-^{1}H COSY spectrum of compound **3** (Acetone-d_6, 500 MHz)

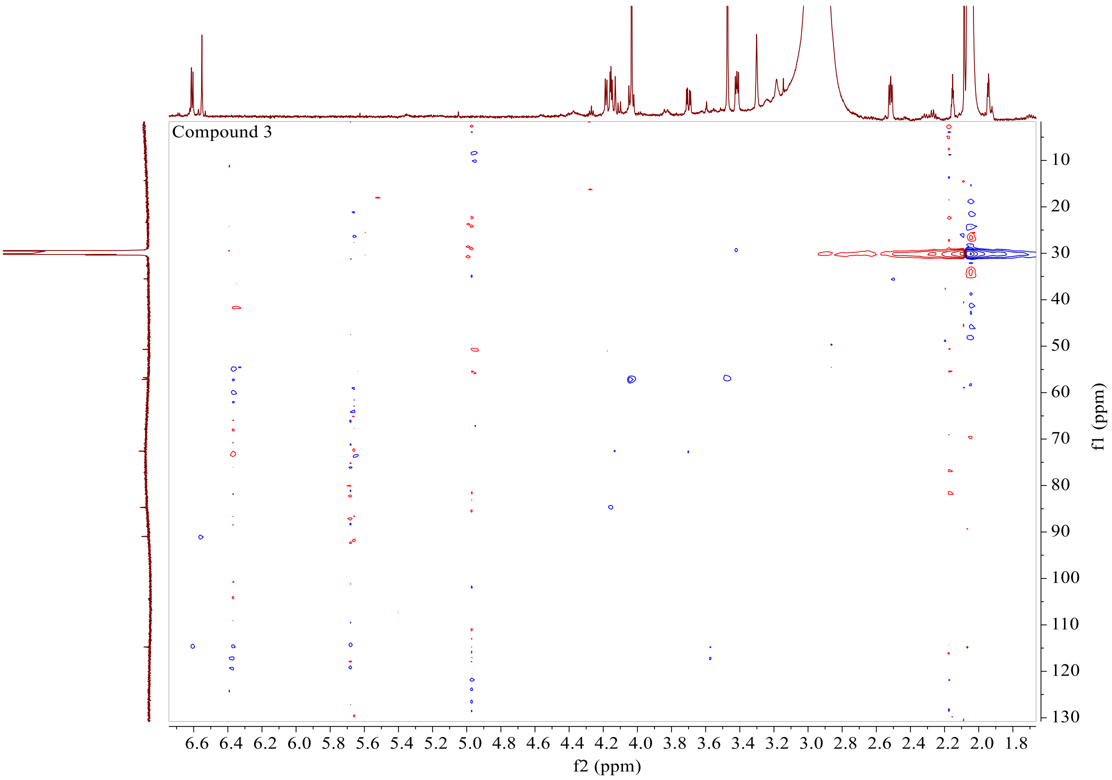

Figure S22 HSQC spectrum of compound **3** (Acetone-*d*6, 500 MHz)

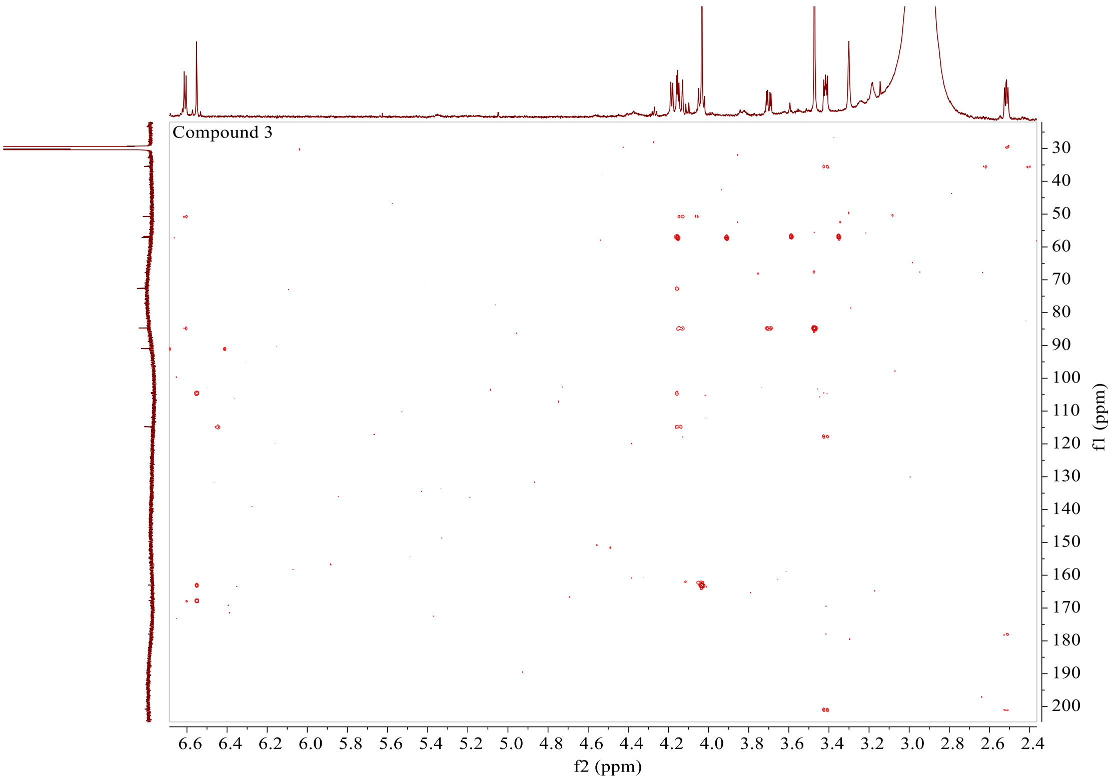

Figure S23 HMBC spectrum of compound **3** (Acetone-*d*6, 500 MHz)

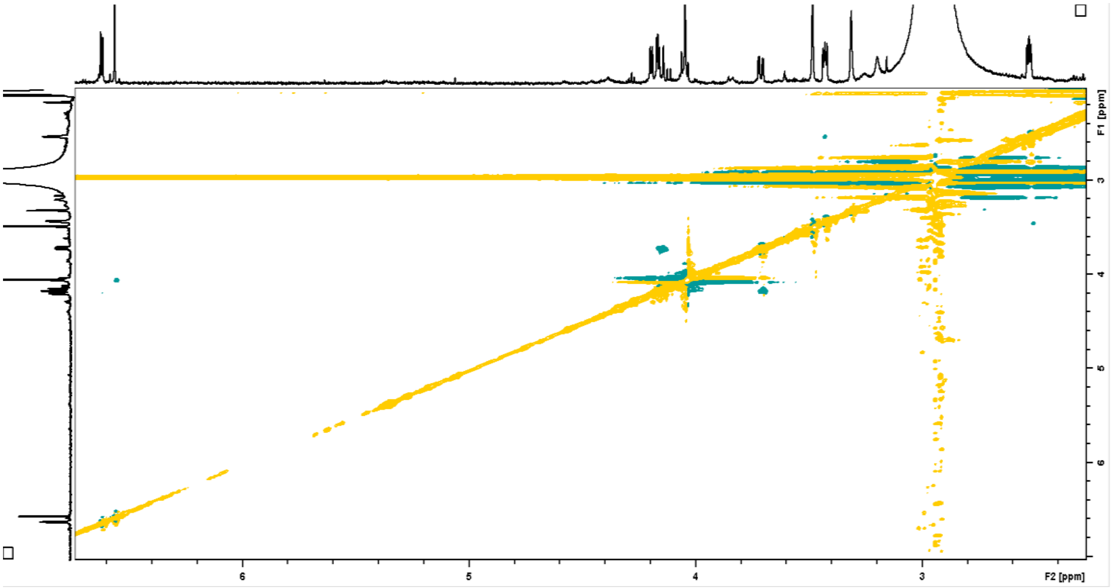

Figure S24 ROESY spectrum of compound **3** (Acetone-*d*6, 500 MHz)

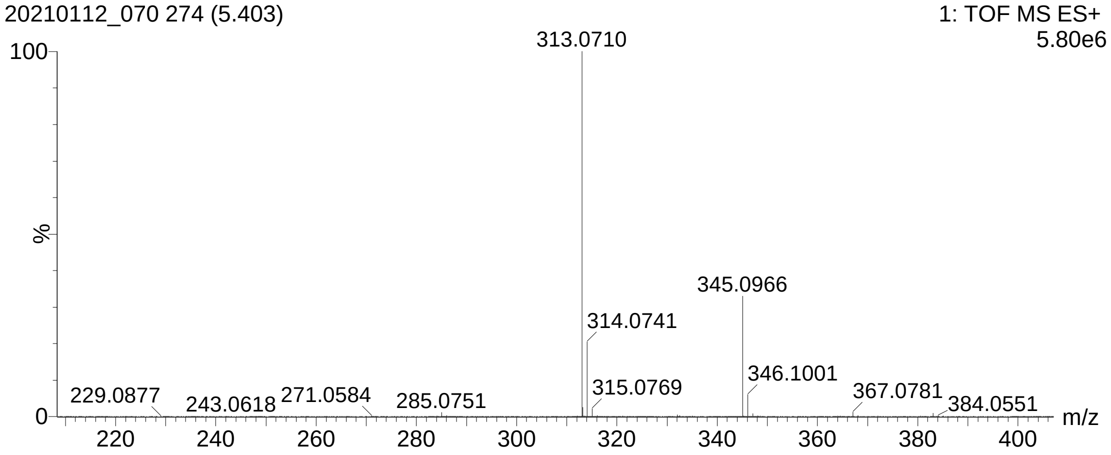

Figure S25 HR-ESI-MS spectrum of compound **3**

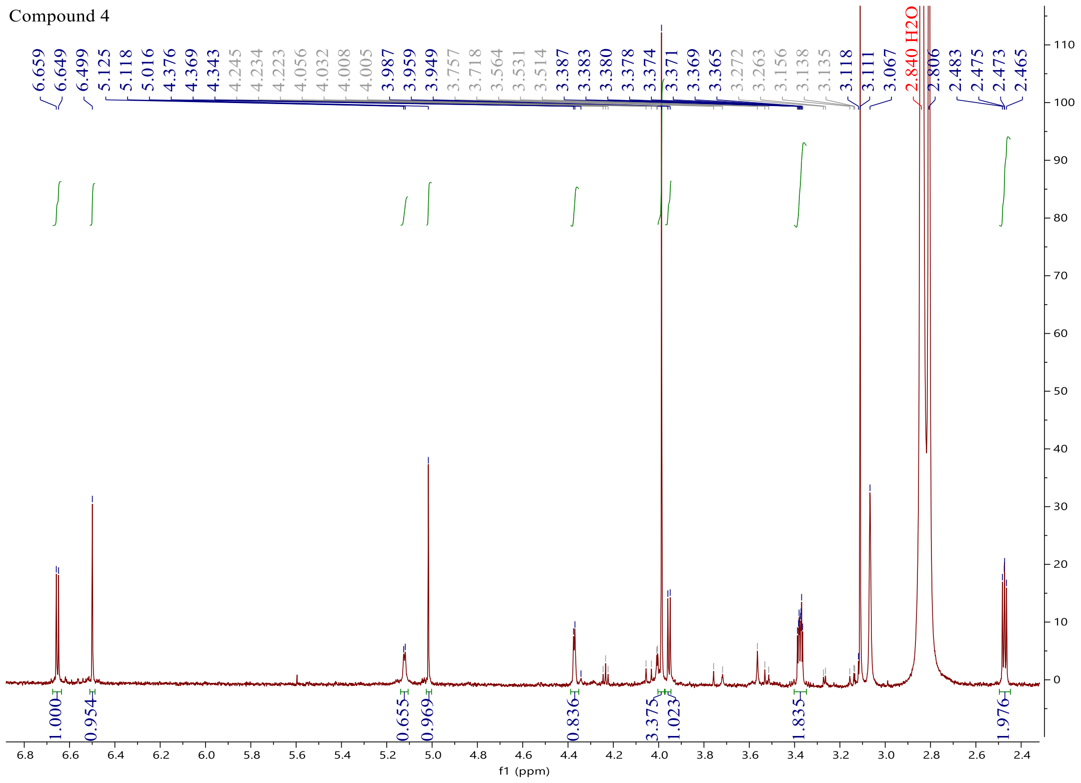

Figure S26 ^{1}H-NMR spectrum of compound **4** (Acetone-d_6, 600 MHz)

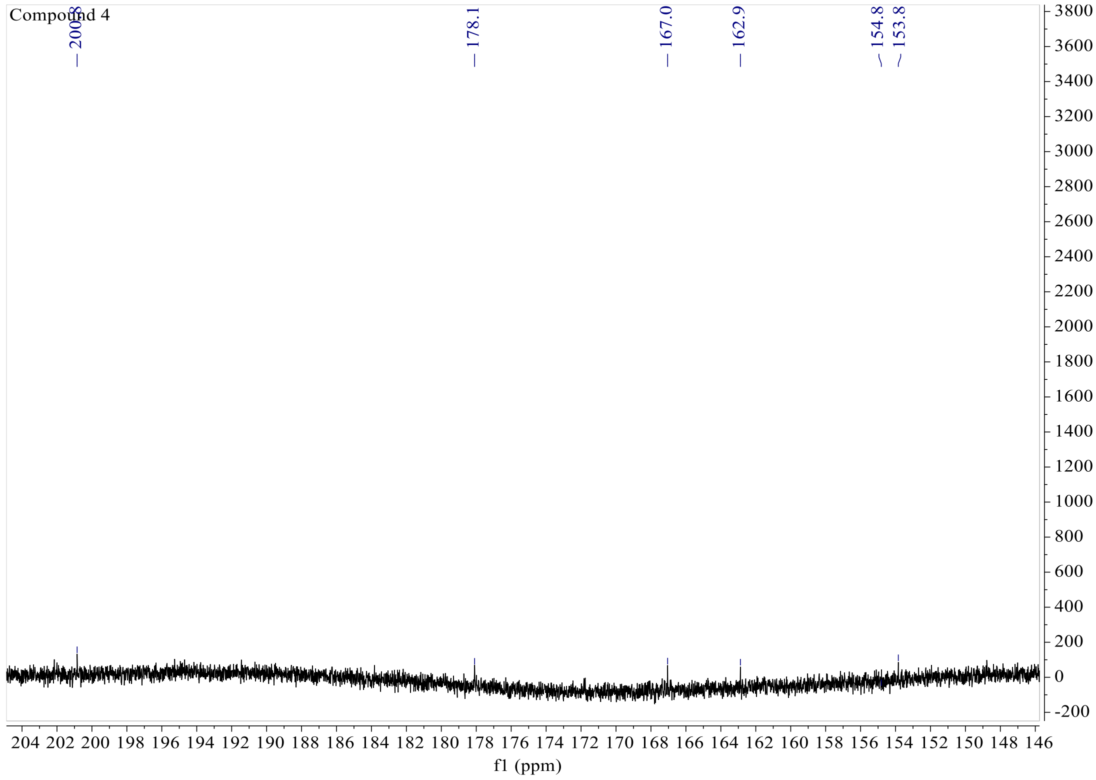

Figure S27-a ^{13}C-NMR spectrum of compound **4** (Acetone-d_6, 125 MHz)

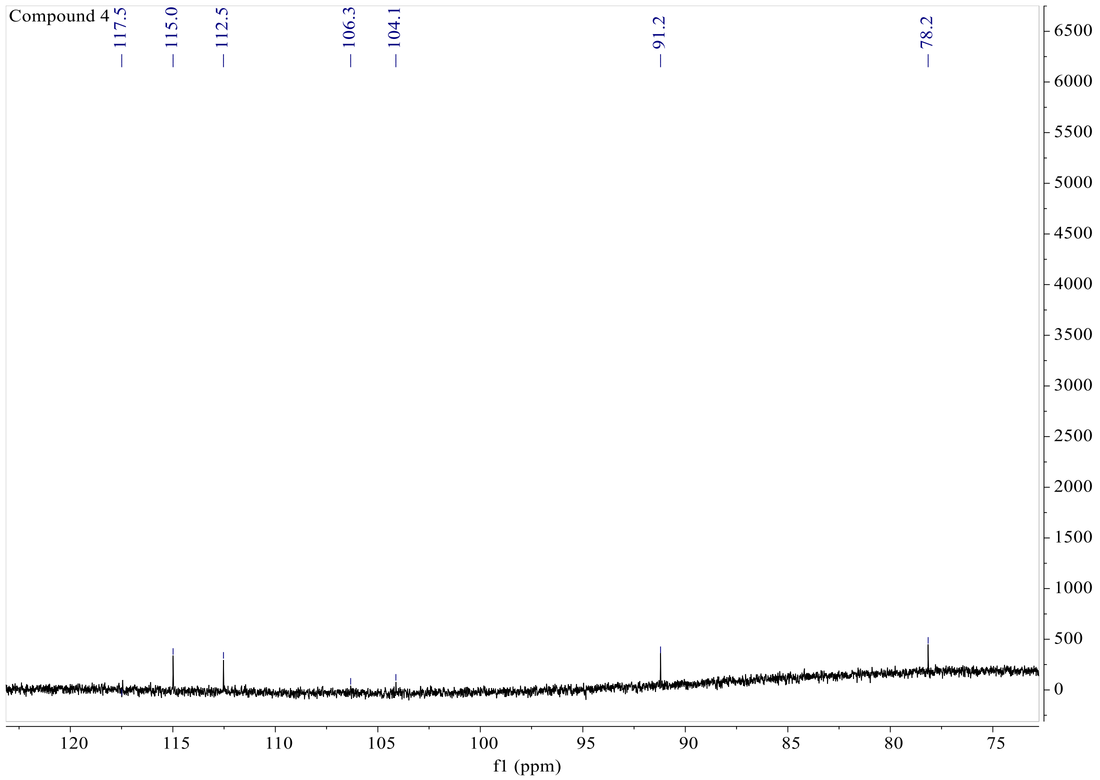

Figure S27-b ^{13}C-NMR spectrum of compound **4** (Acetone-d_6, 125 MHz)

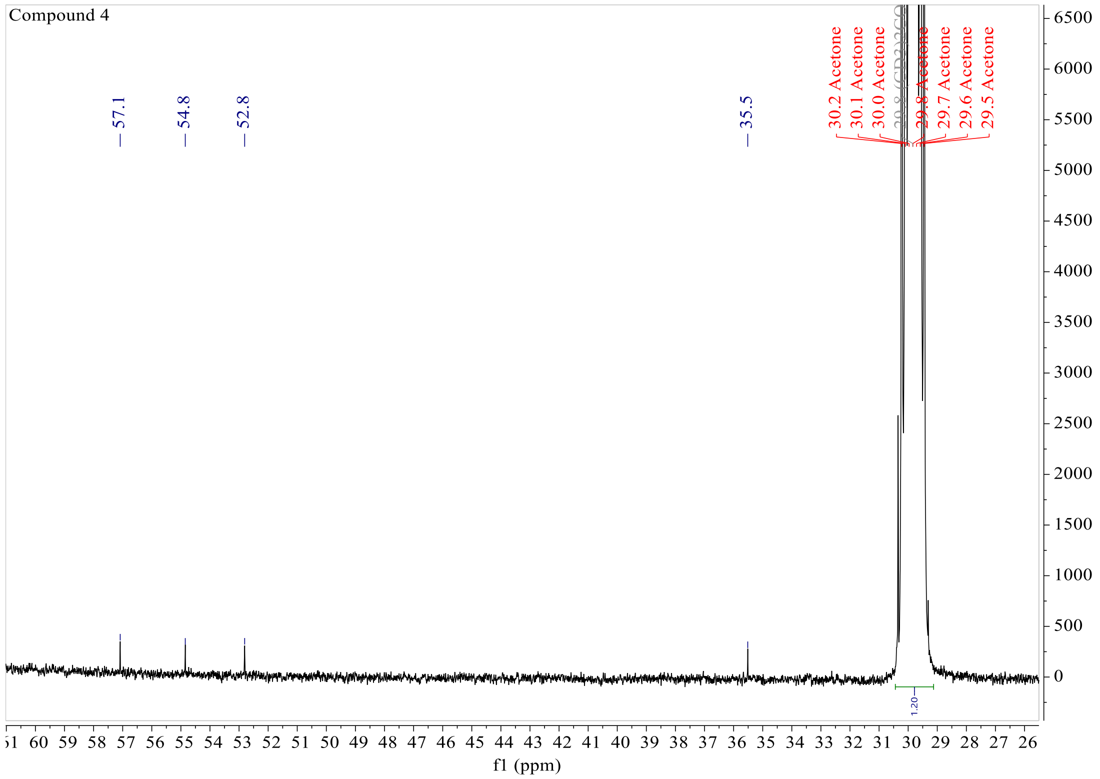

Figure S27-c ^{13}C-NMR spectrum of compound **4** (Acetone-d_6, 125 MHz)

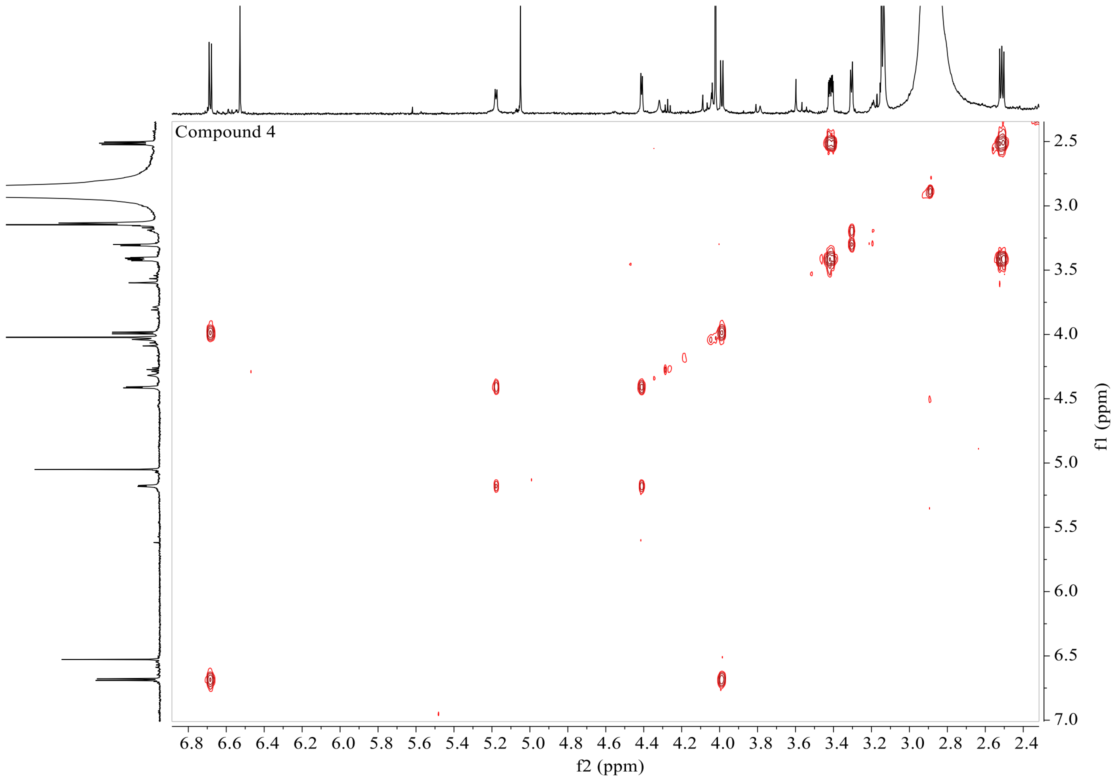

Figure S28 ^{1}H-^{1}HCOSY spectrum of compound **4** (Acetone-*d*$_6$, 500 MHz)

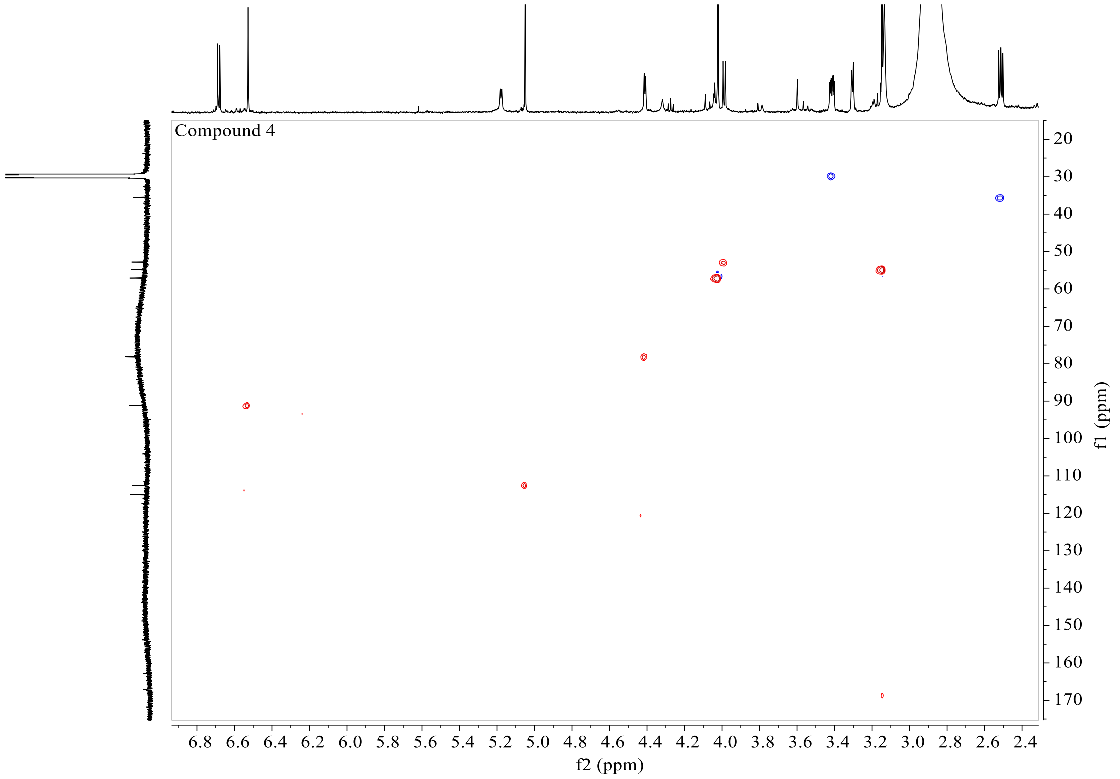

Figure S29 HSQC spectrum of compound **4** (Acetone-*d*$_6$, 500 MHz)

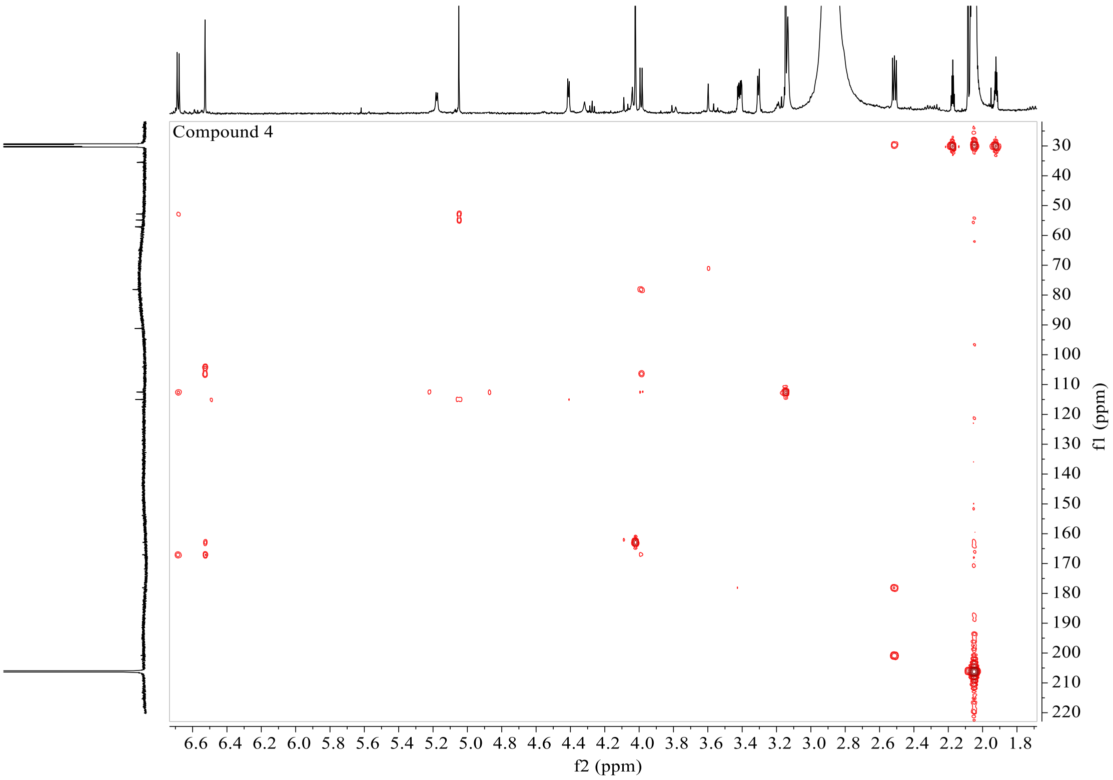

Figure S30 HMBC spectrum of compound **4** (Acetone-d_6, 500 MHz)

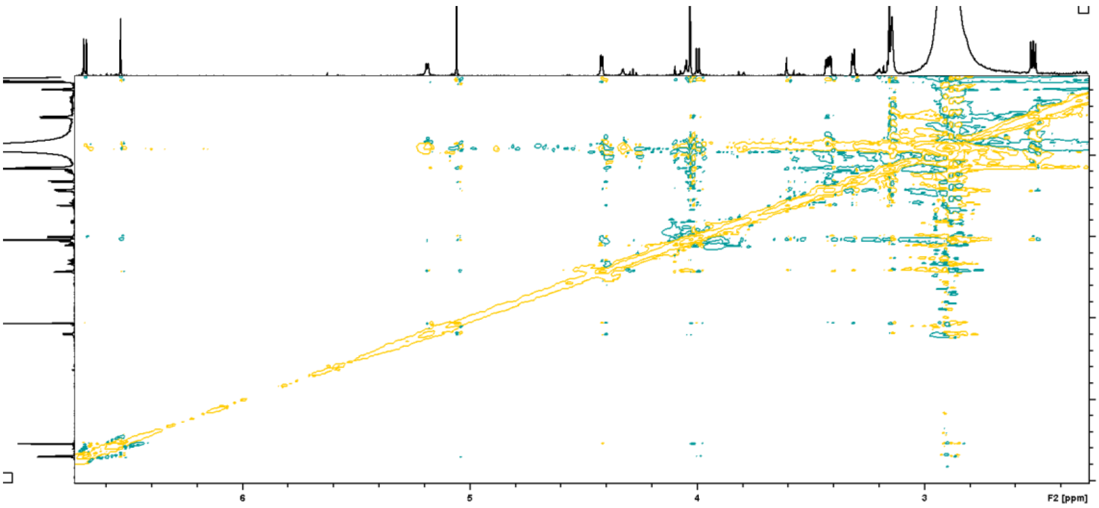

Figure S31 ROESY spectrum of compound **4** (Acetone-d_6, 500 MHz)

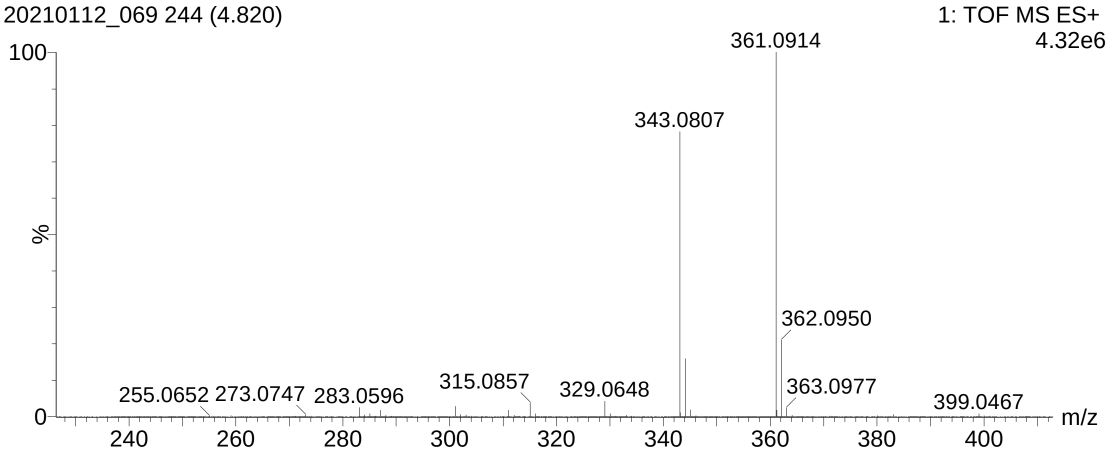

Figure S32 HR-ESI-MS spectrum of compound **4**

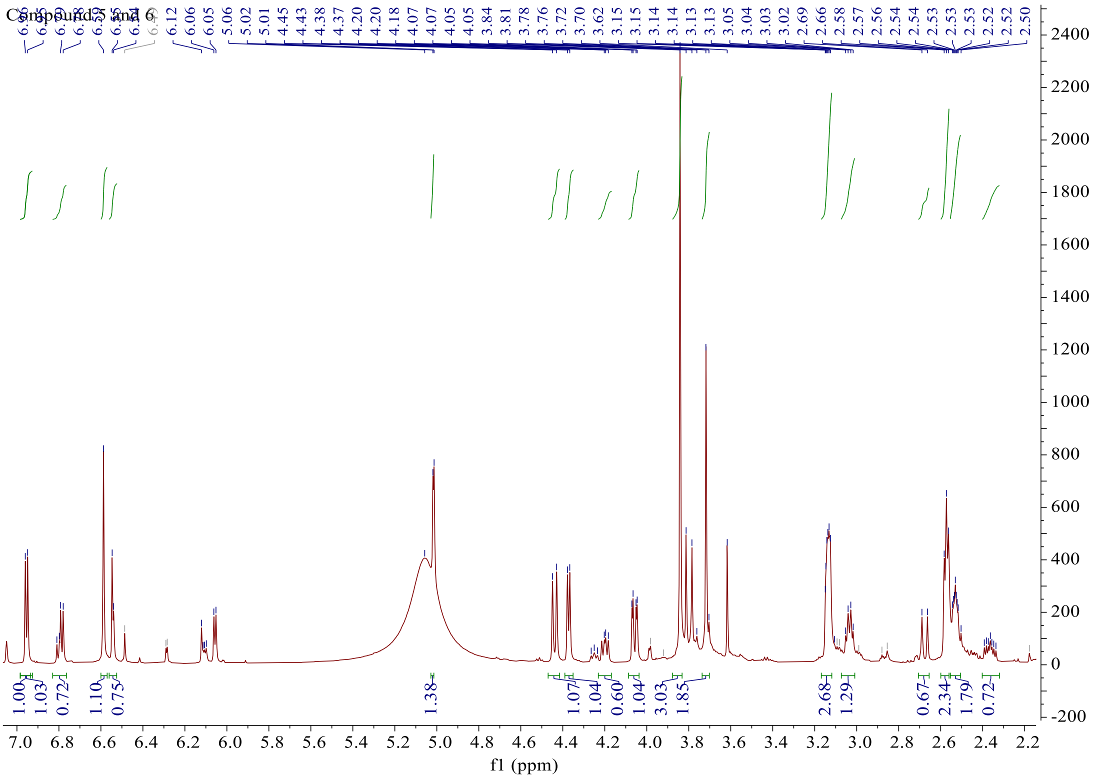

Figure S33 ^{1}H-NMR spectrum of compound **5** and **6** (Pyridine-d_5, 500MHz)

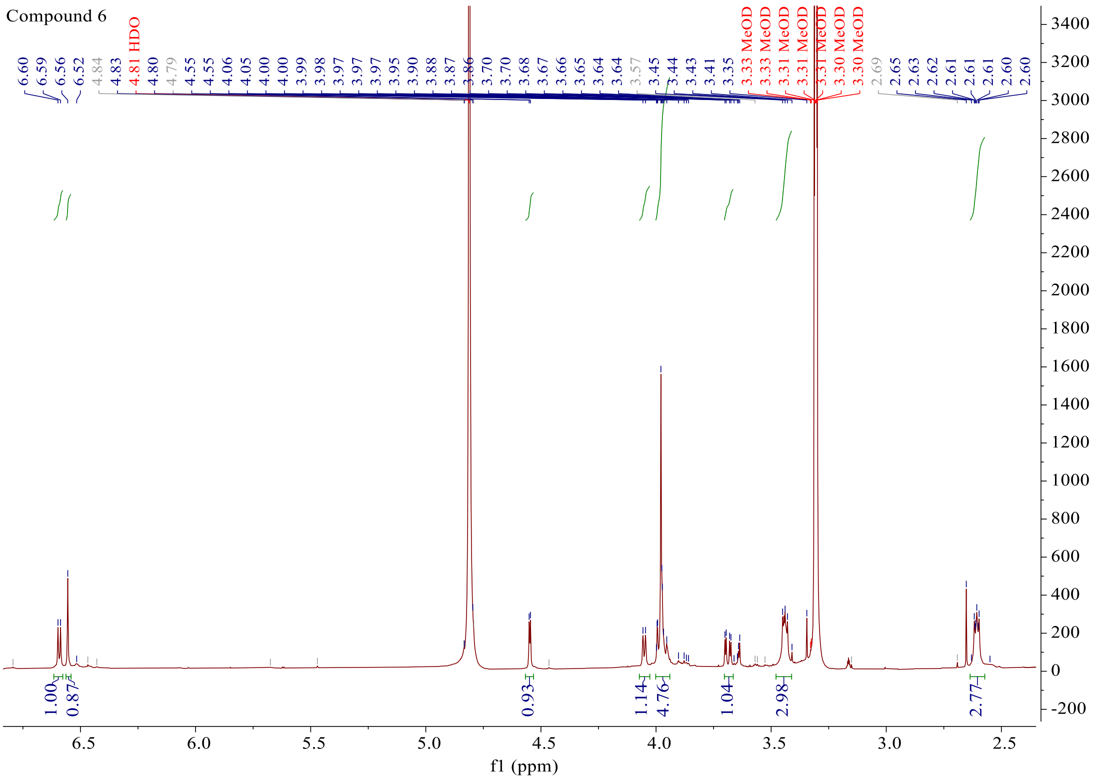

Figure S34 ¹H-NMR spectrum of compound **6** (CD_3OD, 500 MHz)

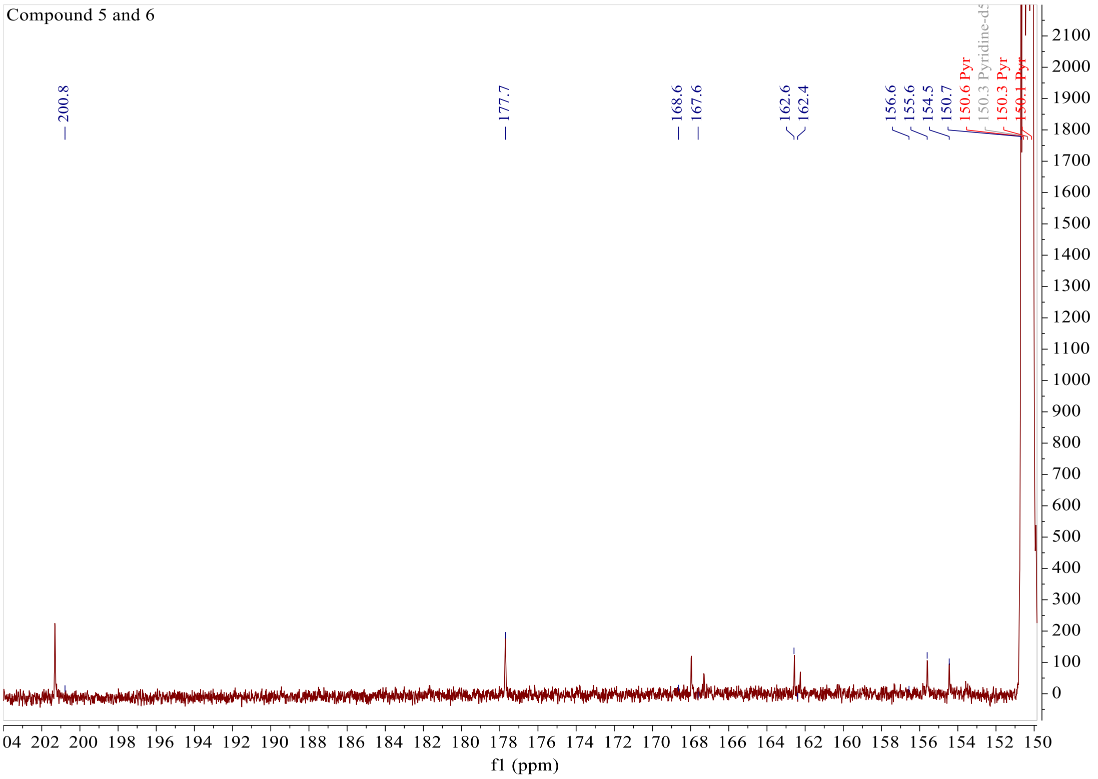

Figure S35-a ¹³C-NMR spectrum of compound **5** and **6** (Pyridine-d_5, 125 MHz)

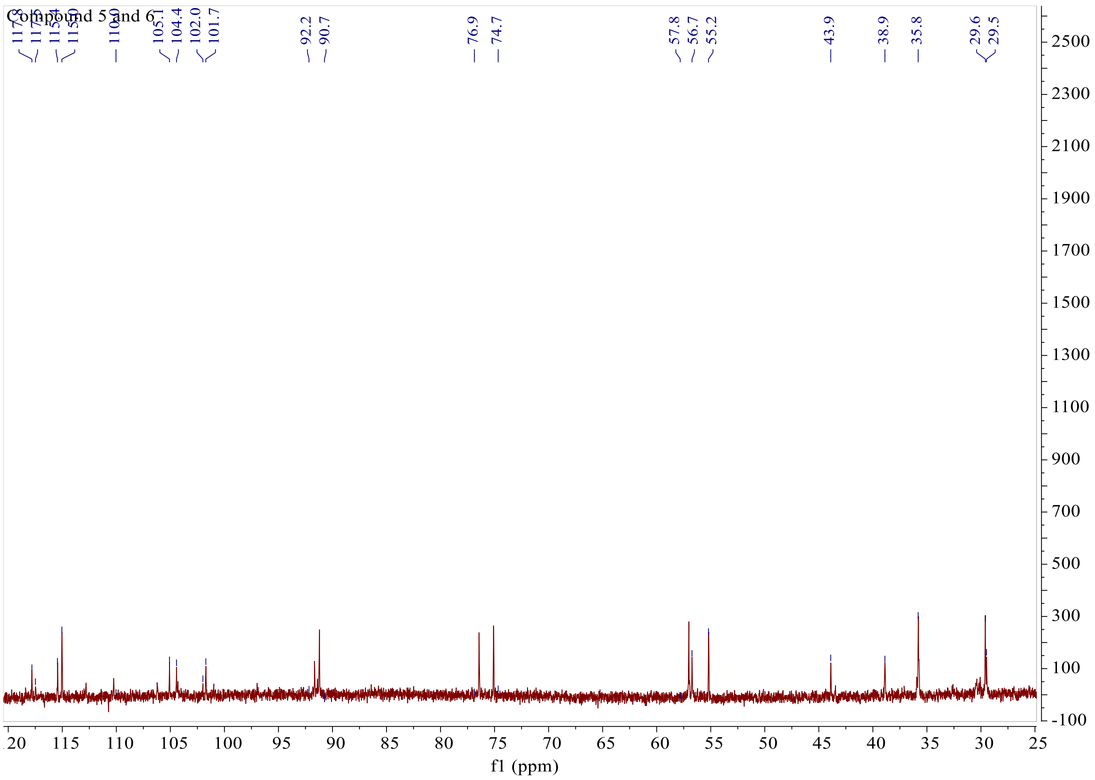

Figure S35-b ^{13}C-NMR spectrum of compound **5** and **6** (Pyridine-d_5, 125 MHz)

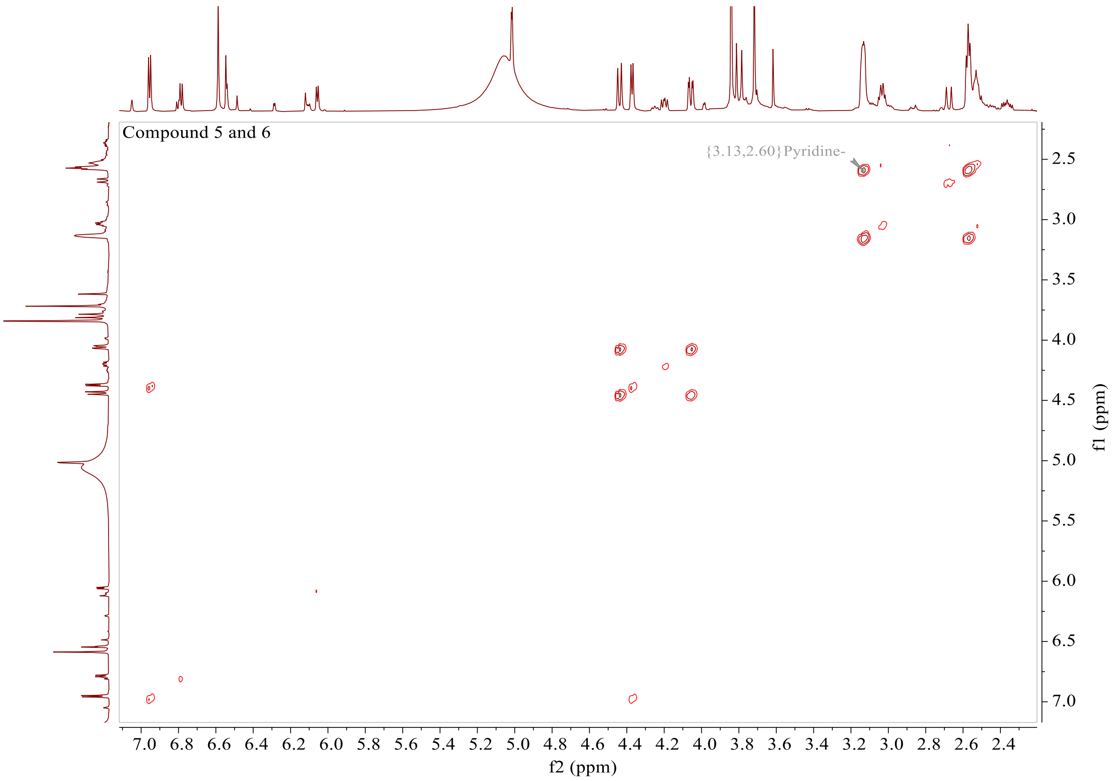

Figure S36 ^{1}H-^{1}HCOSY spectrum of compound **5** and **6** (Pyridine-d_5, 500 MHz)

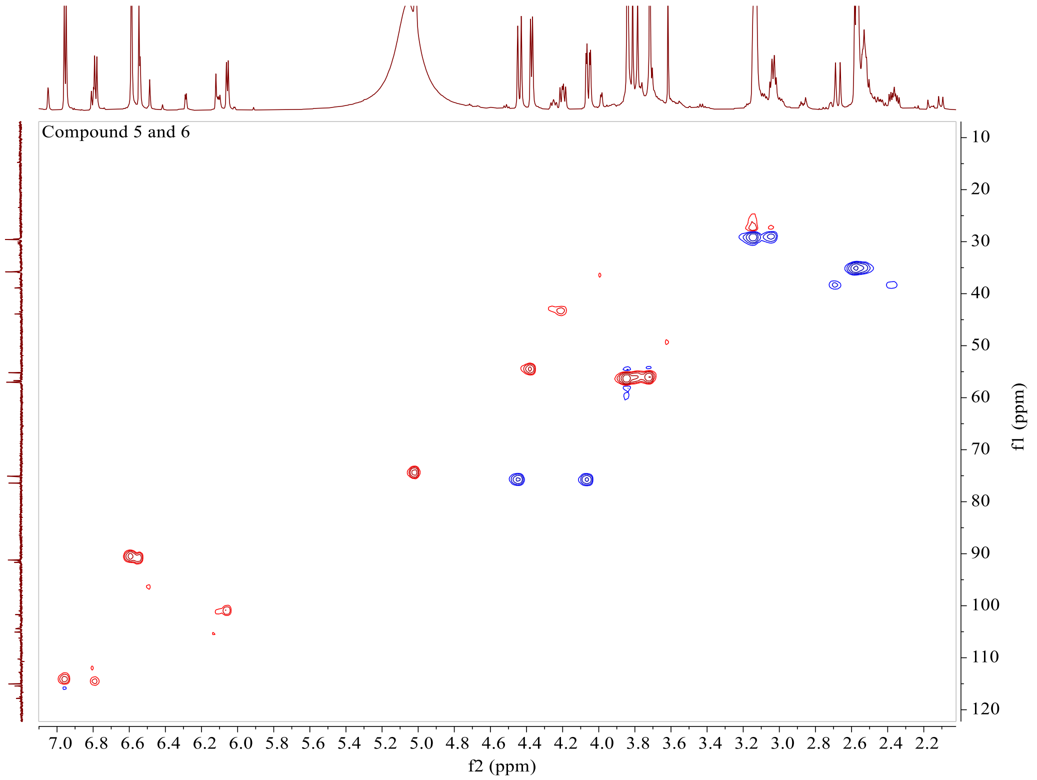

Figure S37 HSQC spectrum of compound **5** and **6** (Pyridine-*d*5, 500 MHz)

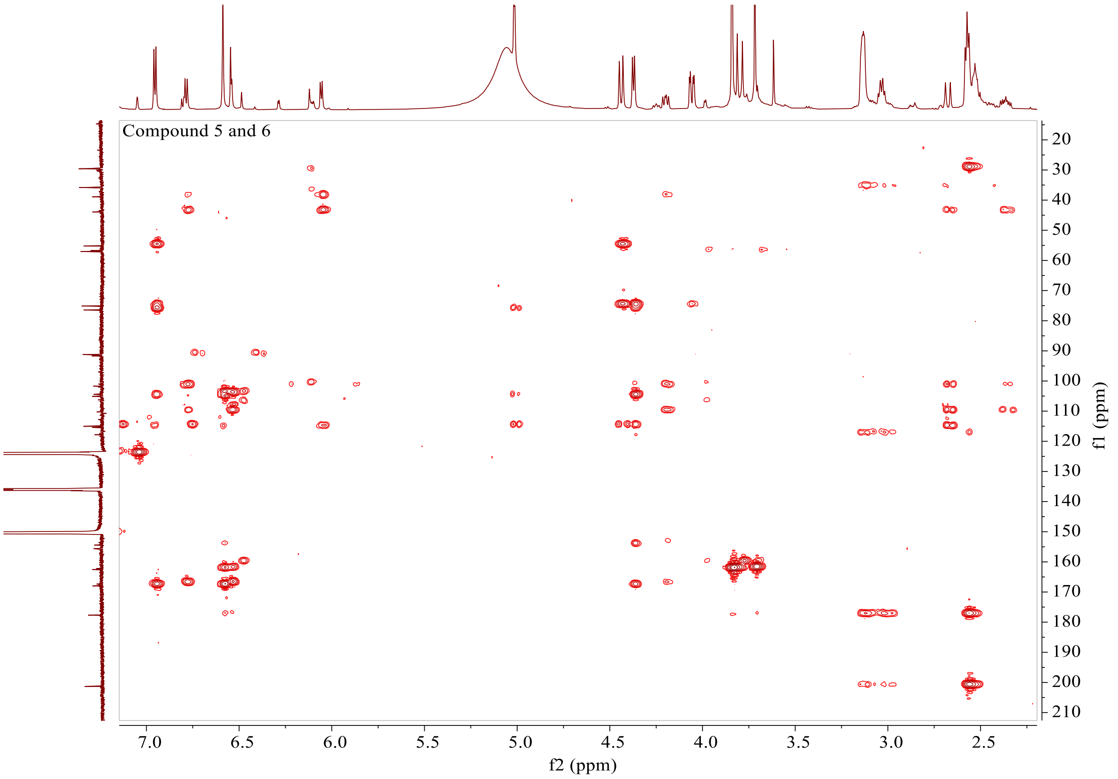

Figure S38 HMBC spectrum of compound **5** and **6** (Pyridine-*d*5, 500 MHz)

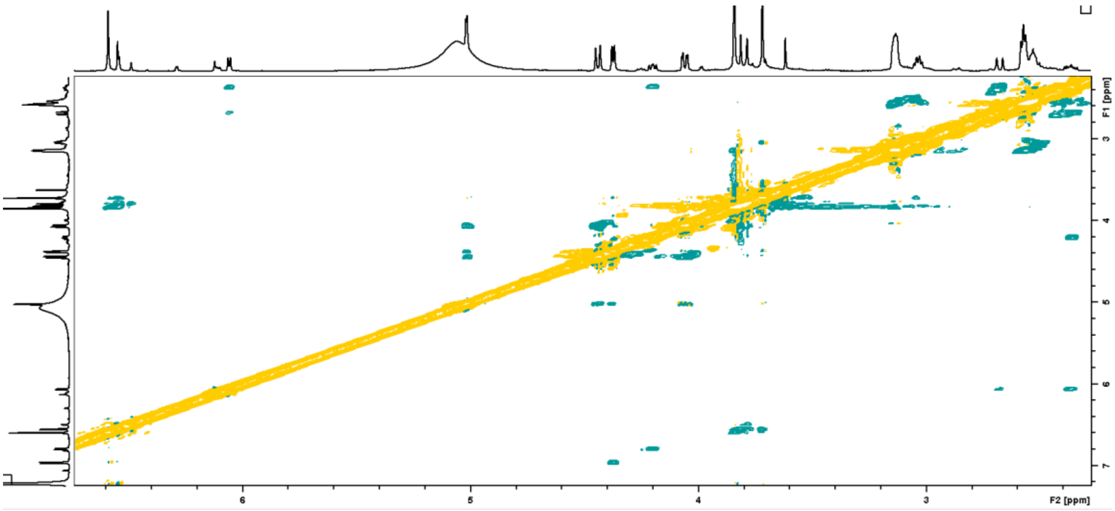

Figure S39 ROESY spectrum of compound **5** and **6** (Pyridine-d_5, 500 MHz)

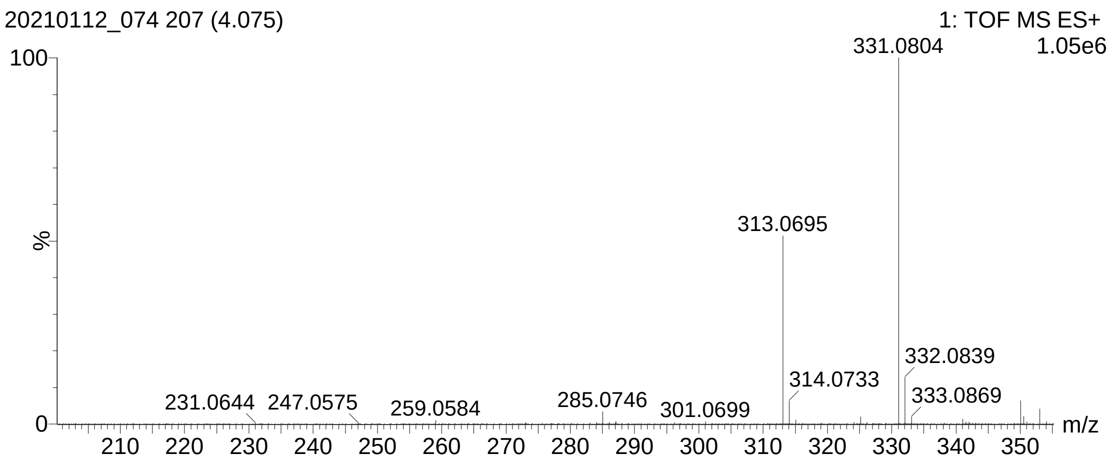

Figure S40 HR-ESI-MS spectrum of compound **5**

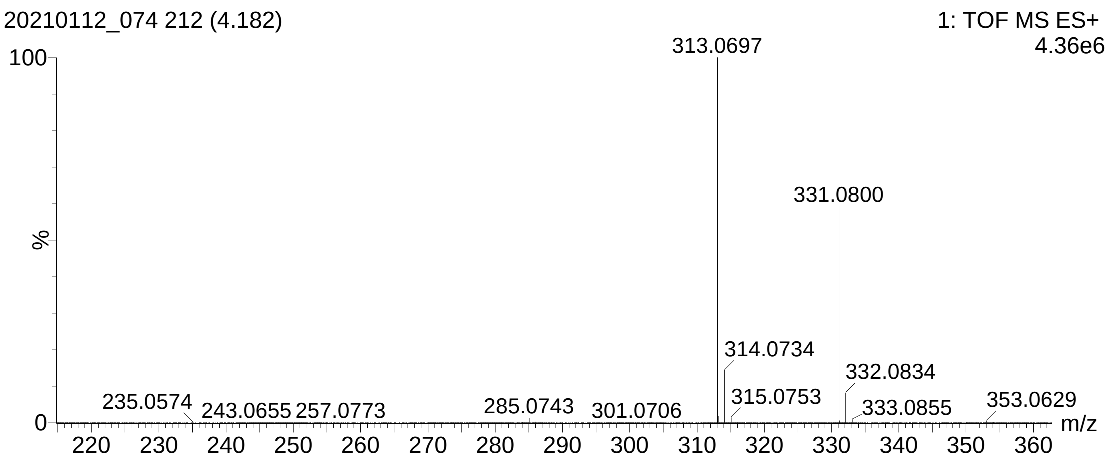

Figure S41 HR-ESI-MS spectrum of compound **6**

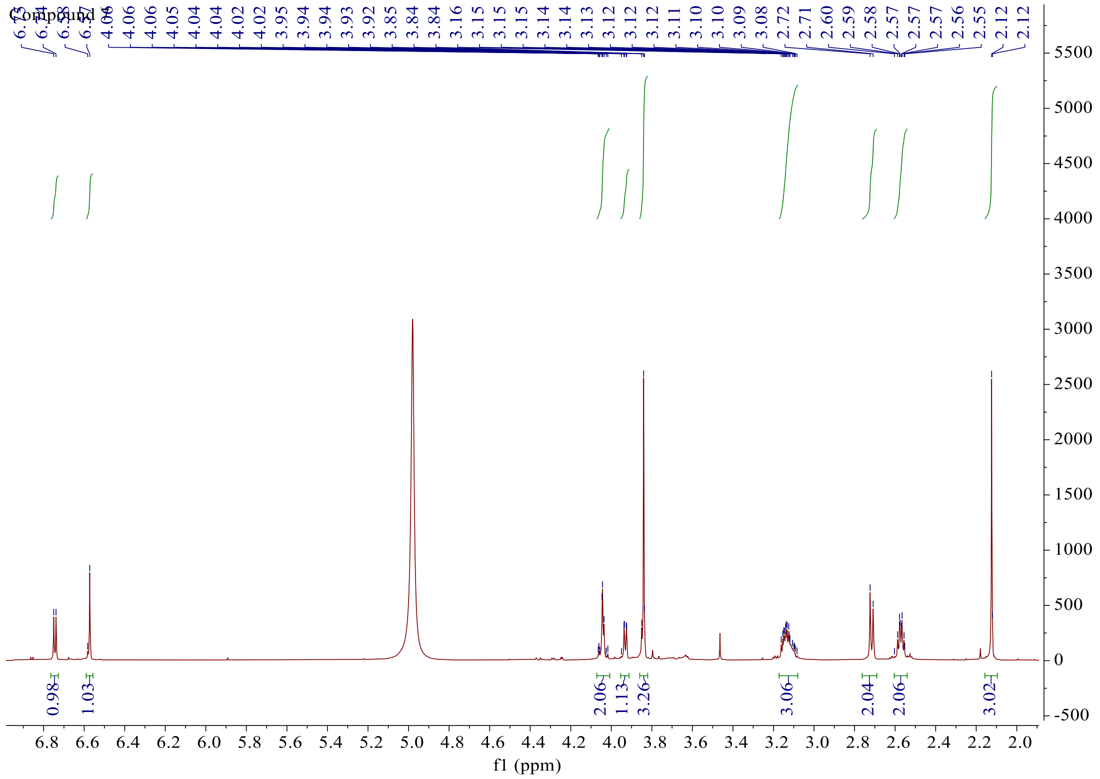

Figure S42 ^{1}H-NMR spectrum of compound **7** (Pyridine-d_5, 500MHz)

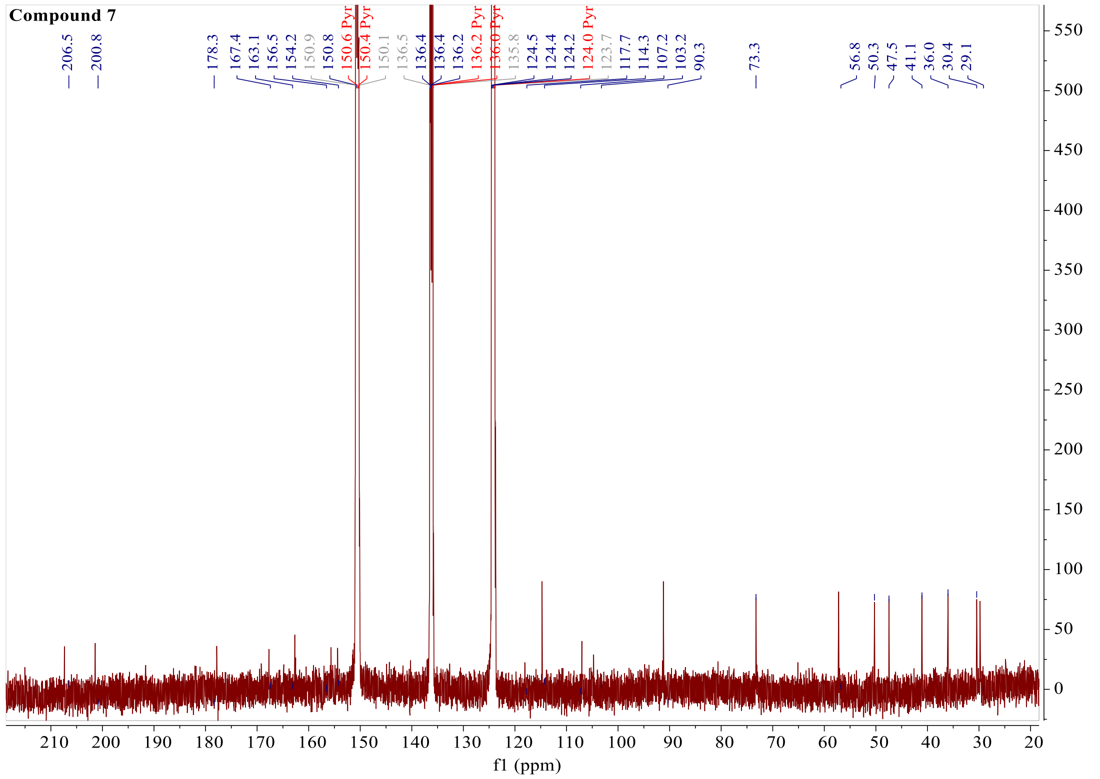

Figure S43^{13}C-NMR spectrum of compound **7** (Pyridine-d_5, 125 MHz)

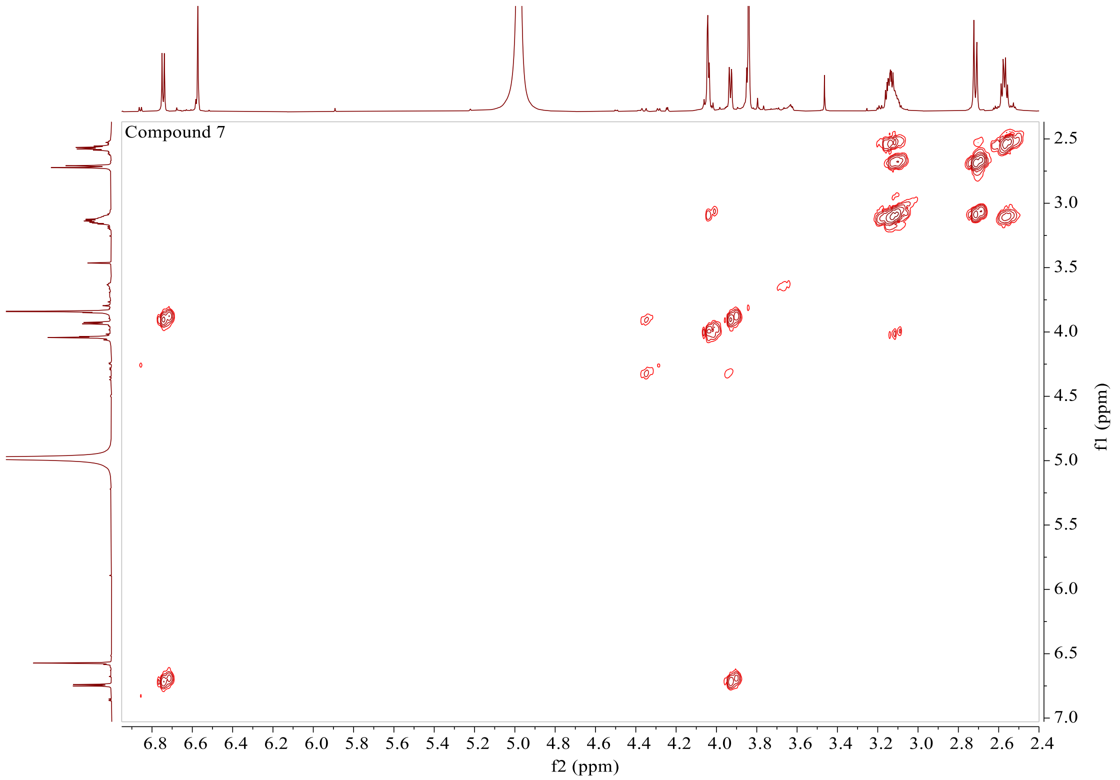

Figure S44 ^{1}H-^{1}HCOSY spectrum of compound **7** (Pyridine-d_5, 500 MHz)

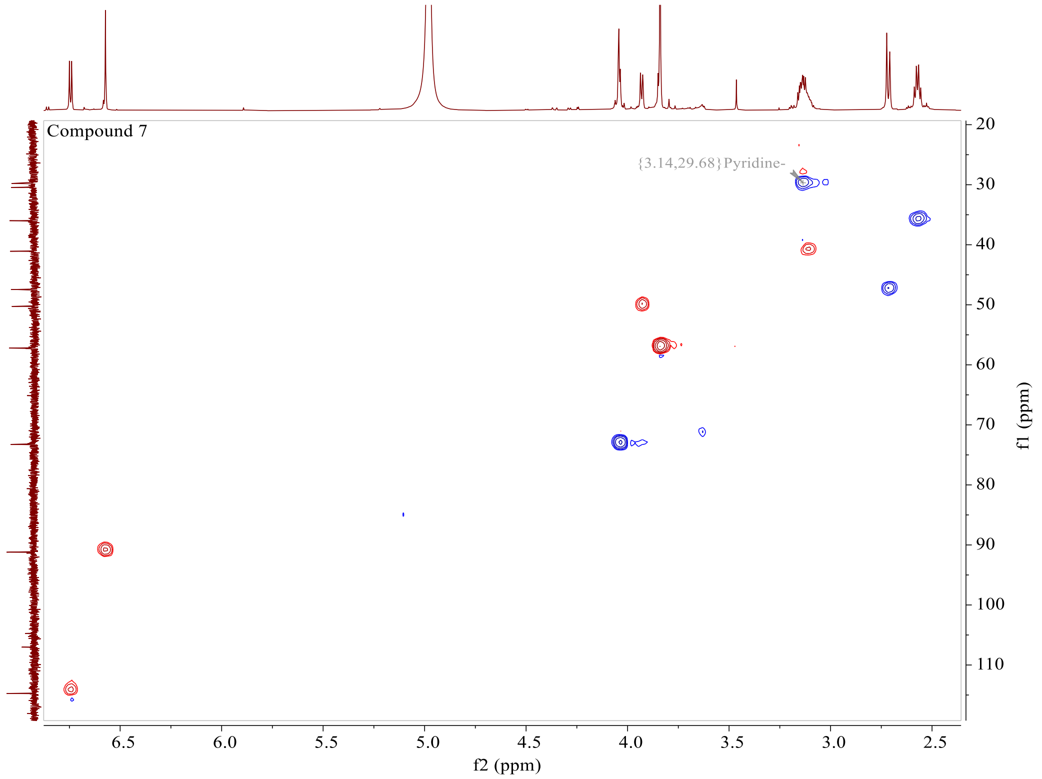

Figure S45 HSQC spectrum of compound **7** (Pyridine-d_5, 500 MHz)

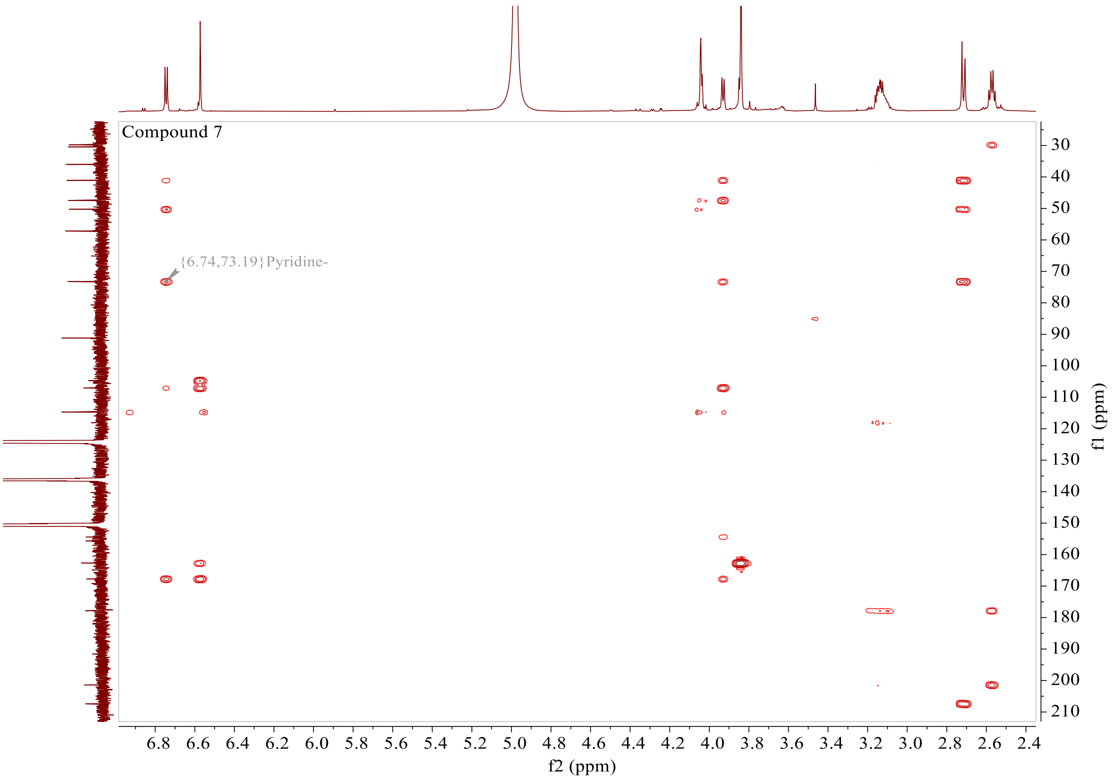

Figure S46 HMBC spectrum of compound **7** (Pyridine-d_5, 500 MHz)

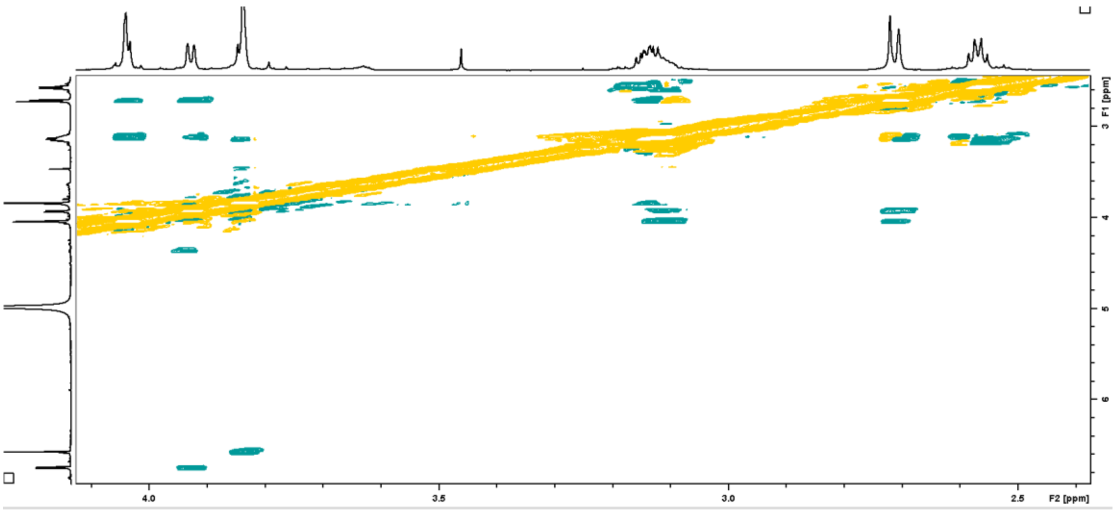

Figure S47 ROESY spectrum of compound **7** (Pyridine-d_5, 500 MHz)

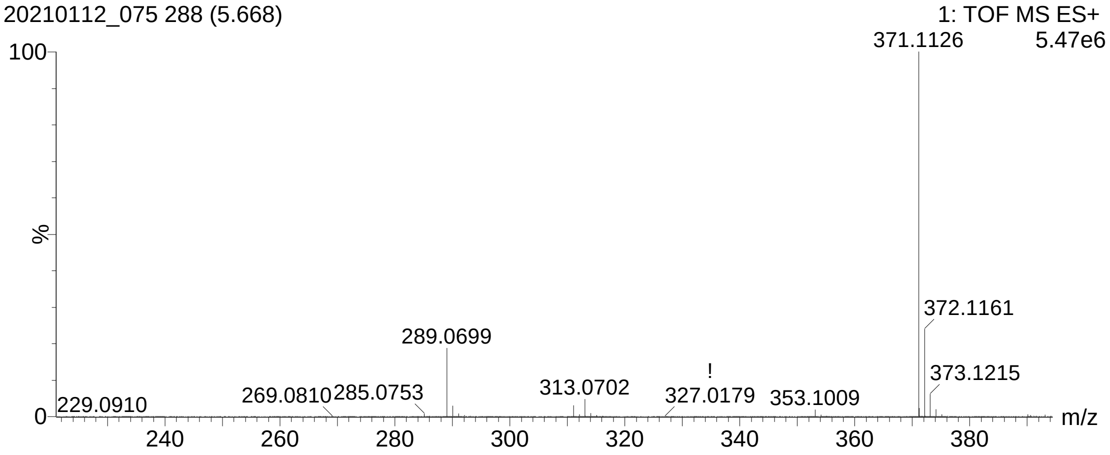

Figure S48 HR-ESI-MS spectrum of compound **7**
